# RotoMate: An open-source, 3D printed autosampler for use with benchtop nuclear magnetic resonance spectrometers

**DOI:** 10.1016/j.ohx.2021.e00211

**Published:** 2021-06-23

**Authors:** Marco Dyga, Christoph Oppel, Lukas J. Gooßen

**Affiliations:** Fakultät für Chemie und Biochemie, Ruhr-Universität Bochum, 44801 Bochum, Germany

**Keywords:** NMR spectroscopy, Benchtop NMR, Autosampler, 3D printing, Automation, Magritek Spinsolve

## Abstract

Benchtop nuclear magnetic resonance (NMR) spectrometers are versatile analytic instruments with low acquisition and operation cost. However, in the basic version, samples must be manually measured one after the other. We herein describe the open-source autosampler RotoMate that allows the automated operation of such instruments. The hardware is easily assembled from 3D-printed and inexpensive off-the-shelf parts, and is controlled by an *Arduino Uno*. A software package interlinks the operation of the autosampler with the software of the NMR spectrometer and the software for the processing of the spectra. Experiments for up to 30 samples can be inserted into an interactive sample list. The autosampler automatically inserts and ejects the samples, initiates measurements on the spectrometer according to parameters specified in the sample list, and interacts with a common NMR software in the processing and visualization of the obtained spectroscopic raw data. If an internal standard is present, conversions and yields of chemical reactions are automatically calculated, enabling e.g. the monitoring of reactions. The device was fitted to a *Magritek Spinsolve* instrument and can interact with a free academic version of ACD NMR software to process the spectra, but can likely be adapted to similar instruments and spectroscopy software packages.

## Graphical abstract

**Figure:**
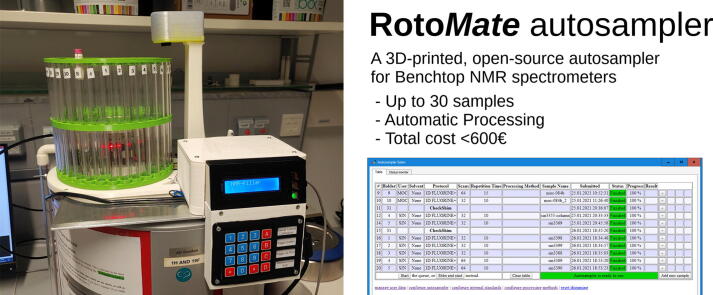

## Specifications table

**Table:** 

Hardware name	RotoMate Autosampler
Subject area	• Chemistry and Biochemistry
Hardware type	• Measuring physical properties and in-lab sensors • Biological sample handling and preparation
Open Source License	The software is published under the GNU General Public Licence v3. The hardware is published under CC-BY 4.0 International.
Cost of Hardware	€510
Source File Repository	https://doi.org/10.5281/zenodo.4627675

## 1 Hardware in context

Nuclear magnetic resonance (NMR) spectroscopy is one of the most powerful and versatile analytical methods available today. NMR allows rapid analysis of homogeneous solutions, providing information about the local chemical environment, connectivity, and other properties of NMR-active nuclei in the sample [1]. Therefore, NMR spectroscopy is routinely applied for structure elucidation and characterization of organic compounds, and has furthermore found a plethora of uses in the fields of chemistry, biochemistry, and materials science. NMR spectroscopy is an inherently quantitative analytical method, as the integral of a signal is proportional to the number of corresponding nuclei [2]. Therefore, quantitative NMR spectroscopy (qNMR) is routinely applied in the analysis of natural products, drugs, and pharmaceuticals [3]. High-quality NMR spectra of all compounds are one of the prerequisites for publishing new chemical substances. However, the use of high-field NMR spectrometers is restricted to larger companies and universities due to their high acquisition and operational costs and the logistics required to keep the helium-cooled superconductive magnets running.

Recently, advances in materials chemistry have led to the development of “benchtop” NMR spectrometers. They have high-performance permanent magnets rather than helium-cooled superconductive magnets, which dramatically lowers the acquisition and maintenance cost – especially when purchased without any expensive accessories [4]. Their resolution is markedly lower than that of traditional NMR spectrometers, but is still sufficient for many applications such as quality control [5], forensic analysis [6], on-line reaction monitoring [7], and academic teaching [8]. In contrast to chromatographic methods, analysis of samples by NMR has the key advantage that the samples stay inside glass tubes and the spectra are obtained contact-free. Thus, the spectrometer does not get contaminated by the chemicals inside samples, and there are no chromatographic phases that need to be replaced regularly or must be exchanged after analysing a corrosive or toxic sample. This further reduces operation cost. Since there are no moving parts, the maintenance only consists of regularly running shim routines, and this does not require trained personnel.

The limiting factor in the operation of the instrument is that the shimming routines have to be started manually, and that every sample must be manually inserted into the spectrometer, each time typing in the details of the desired experiment. In our laboratories, we routinely use *Magritek Spinsolve* benchtop NMRs for the analysis of reaction mixtures. When we purchased the instruments, no autosampler system was available, so that the students had to operate it manually. This turned out to be a time-consuming and unpopular task, since we sometimes analysed hundred samples per day, and every few minutes, a new sample had to be inserted. In order to relieve us from this repetitive task, we constructed RotoMate, a low-budget autosampler, and programmed a software to control it. It has been in service for several years and has greatly simplified the operation of the instrument.

Meanwhile, commercial autosamplers have become available, but RotoMate still compares favourably. It has space for 30 queued samples, whereas the leading commercial autosampler can only handle 20 samples. In contrast the proprietary instrument, the software further processes the spectra and is even able to calculate relative contents, conversions, or yields, if an internal standard is provided. Still, the cost of building a RotoMate is only a small fraction of the price for a proprietary autosampler.

## 2 Hardware description

**Fig. 1:**
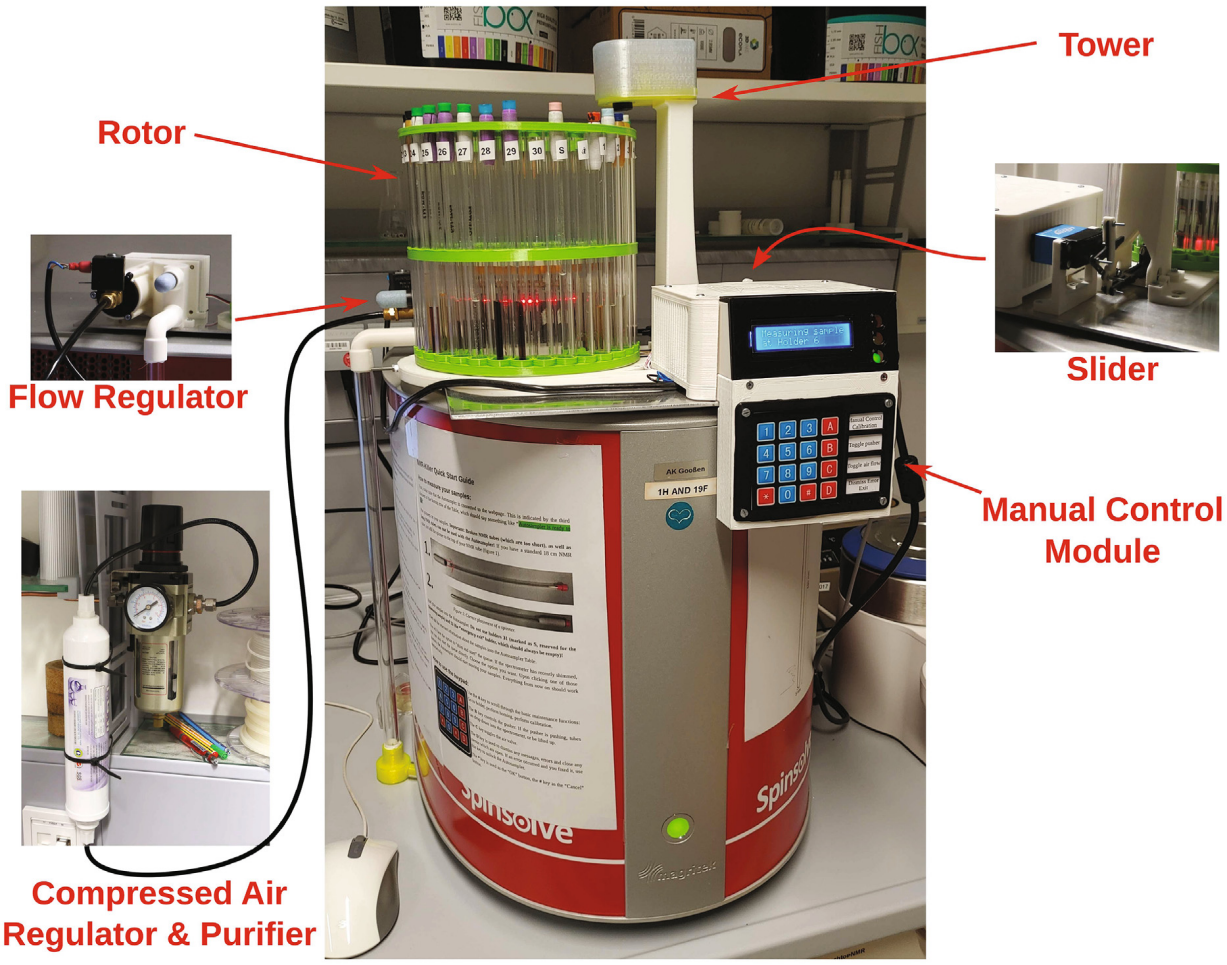
Overview of the RotoMate autosampler mounted on a Magritek Spinsolve 43 MHz spectrometer. For a detailed description, see below.

The RotoMate autosampler (Fig. 1) is a robot which automates the process of inserting and ejecting samples into or from a benchtop NMR spectrometer, e.g. from the *Magritek Spinsolve* series of spectrometers. Its largest part is the “rotor”, which holds the NMR samples and is driven by a stepper motor, moving the next sample to the centre hole of the spectrometer. A trapdoor system, hereafter referred to as “slider”, usually blocks the hole through which the samples are lowered into the spectrometer. It is opened via a lever mechanism driven by a servo motor, allowing sample tubes positioned above it in the rotor to be lowered into the spectrometer. The sample tubes are moved up and down by modulating a stream of compressed air with the help of the “flow regulator”. For safety reasons, this process is monitored with light barriers. The presence of a sample is checked by moving it up in a stream of compressed air towards a light barrier installed at the “tower” above the trapdoor. The “manual control module” allows the operator to control the device without connection to a PC. Detailed descriptions of the functions of each part can be found in the build instructions (Section 5, “*Build Instructions*”).

**Fig. 2:**
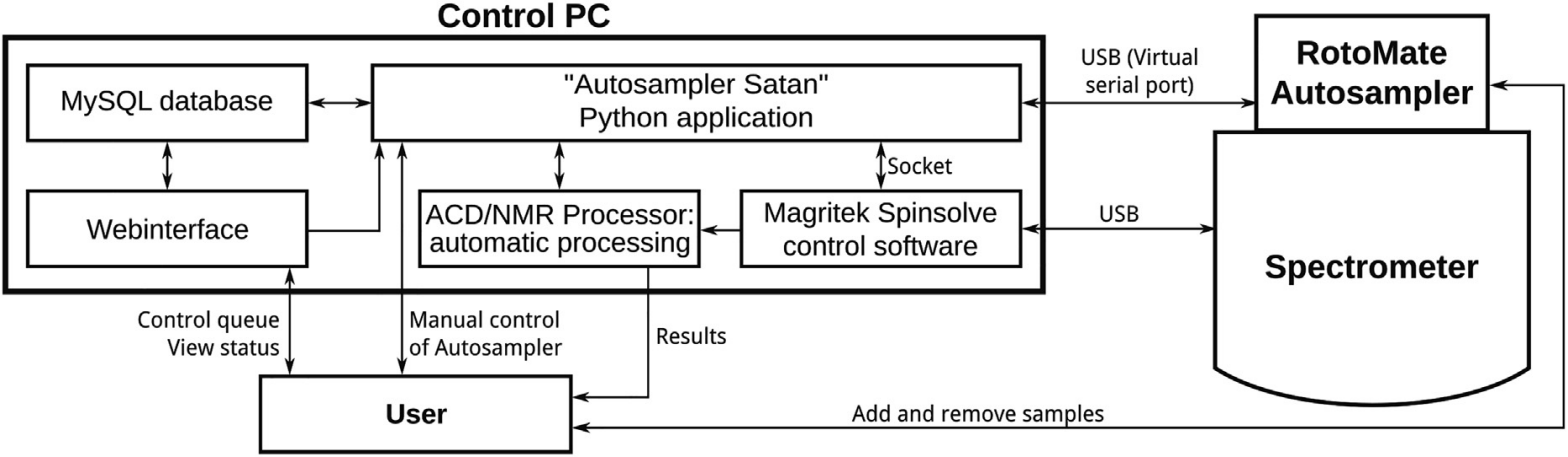
Interplay between the software and hardware components of the RotoMate autosampler, Spinsolve spectrometer, and user.

All subunits are controlled by an *Arduino Uno* microcontroller, which is connected to a PC via USB. The *Arduino* continuously sends the current status of the autosampler to the PC. The software that controls the NMR instrument must be installed on this PC (in our case the *Spinsolve* software package). Similar to most other software packages for analytic instruments, this NMR program can be conFig.d to accept commands and send status messages, in this case via a socket. This is described in detail in the manual of the spectrometer. A PHP-capable webserver and a MySQL database must also be installed. Adding, editing, and removing samples, as well as starting and stopping the queue, are handled by a webinterface, which also allows to remotely monitor the status of the autosampler and the currently running measurement with any browser. The sample queue is stored in the MySQL database along with the settings and other parameters. A python software with access to the virtual serial port of the autosampler, the socket of the *Spinsolve* software, and the MySQL database, continuously runs in the background and coordinates all components. The python software also provides a graphical user interface (GUI), allowing to manually control the autosampler and access the webinterface with an integrated browser. If a queue is started, the python script will 1) read the parameters for the next sample from the MySQL database, 2) send a command to the autosampler to insert the sample, 3) wait until the autosampler reports successful insertion, and if no error is encountered, 4) start the measurement in the *Spinsolve* software, 5) wait until the spectrometer reports that the measurement is done without error, 6.) then send a command to the autosampler to return the sample to the rotor. This process is repeated until all samples have been measured or an error occurs (See Fig. 2).

As an optional feature, automatic processing of spectra is implemented in the RotoMate’s software. It is set up to interact with *ACD NMR Processor*, which was available as a free academic edition. The automatic sample processing includes baseline correction, picking a reference peak, automatic integration against an internal standard, and generation of a PDF and JDX [9] file. The commands are sent to *ACD* via a macro file. Due to the open-source nature of the RotoMate, it should be possible to adapt it to any other NMR processing software that is capable of running pre-defined macros.

Since the RotoMate is fully open-source in both hardware and software, it should also be possible to adapt our design to other models of benchtop NMR spectrometers. This would mainly involve adjusting the mounting bracket, the size of the spinners, and the connection to the compressed air system. In addition, the measurement methods (^19^F, ^1^H, Shim), as well as the communication of the Python software with the spectrometer would also have to be reimplemented for that particular model of spectrometer.

Overall, the RotoMate autosampler simplifies NMR analysis as follows:

• its web interface allows to insert an entire list of spectroscopic experiments
• up to 30 different samples can be placed in the spectrometer
• the spectroscopic measurements are automatically performed for all samples
• the spectra are automatically processed
• the amounts of specified components against an internal standard can be optionally calculated

## 3 Design files

A detailed list of CAD files can be found in the Bill of Materials; a detailed list of source code files can be found in the GitHub repositories listed below.

### Design files summary

**Table:** 

Designation	File type	Open source license	Location of the file
CAD files	CAD files	CC-BY 4.0	https://doi.org/10.5281/zenodo.4627675
Template for drilling holes	Fig.	CC-BY 4.0	https://doi.org/10.5281/zenodo.4627675
Labelling for Keypad	Fig.	CC-BY 4.0	https://doi.org/10.5281/zenodo.4627675
Schematic	Fig. & Fritzing schematic	CC-BY 4.0	https://doi.org/10.5281/zenodo.4627675
Spinners Tutorial Video	Video	CC-BY 4.0	https://doi.org/10.5281/zenodo.4627675
Rotor Calibration Video	Video	CC-BY 4.0	https://doi.org/10.5281/zenodo.4627675
Slider Calibration Video	Video	CC-BY 4.0	https://doi.org/10.5281/zenodo.4627675
Air Calibration Video	Video	CC-BY 4.0	https://doi.org/10.5281/zenodo.4627675
Rotor Alignment Video	Video	CC-BY 4.0	https://doi.org/10.5281/zenodo.4627675
Pressure Video	Video	CC-BY 4.0	https://doi.org/10.5281/zenodo.4627675
Pictures	Images	CC-BY 4.0	https://doi.org/10.5281/zenodo.4627675
nmr_autosampler_arduino	Firmware	GPLv3	https://github.com/marcodyga/nmr_autosampler_arduino
			https://doi.org/10.5281/zenodo.4627720
nmr_autosampler_python	Software	GPLv3	https://github.com/marcodyga/nmr_autosampler_python
			https://doi.org/10.5281/zenodo.4947822
nmr_autosampler_webapp	Software	GPLv3	https://github.com/marcodyga/nmr_autosampler_webapp
			https://doi.org/10.5281/zenodo.4947809

*CAD files:* The CAD files for the autosampler in STL format and as Autodesk Inventor part files (IPT).

*Template for drilling holes:* A template to be printed on an A3 paper sheet, which allows to mark the positions of the mounting holes that need to be drilled into the aluminium baseplate (Section 5.1.1, “*Aluminium baseplate with funnel*”).

*Labelling for Keypad:* A PDF file which can be printed out and attached as a legend to the keypad (Section 5.1.5, “*Electronics*”).

*Schematic:* Shows the wiring of the autosampler. It is provided as a PDF, as well as a Fritzing [10] file for interactive viewing.

*Spinners Tutorial Video:* A video which explains how post process the 3D-printed spinners so that they stick to the sample tubes and easily slide within the rotor and the instrument tubings (Section 5.1.9, “*Spinners”*).

*Rotor Calibration Video:* A video tutorial for the calibration of the rotor (Section 5.4.1, “*Calibration of the rotor*”).
*Slider Calibration Video:* A video tutorial for the calibration of the slider (Section 5.4.2, “*Calibration of the slider*”).
*Air Calibration Video:* A video tutorial for the calibration of the airflow regulator (Section 5.4.3, “*Calibration of the airflow regulator*”).
*Rotor Alignment Video:* A video showing the alignment of the rotor (Section 5.3.2, “*Fine-tuning the air pressure, slider, and rotor placement*”).
*Pressure Video:* A video showing adjustment of the air pressure (Section 5.3.2, “*Fine-tuning the air pressure, slider, and rotor placement*”).
*Pictures:* A folder containing high-resolution versions of the images and Fig.s in this article.
*nmr_autosampler_arduino:* The Arduino [11] code for the autosampler.
*nmr_autosampler_python:* The Python code for the autosampler.
*nmr_autosampler_webapp:* The code for the web interface of the autosampler.

## 4 Bill of materials

A Bill of Materials is provided in the Supplementary Information. At the beginning of each subchapter in the hardware assembly instructions (Chapter 5.1), a list and photo of the parts used in this subchapter are given.

## 5 Build instructions

### 5.1 Hardware assembly

#### 5.1.1 Aluminium baseplate with funnel

The instructions below allow to mount RotoMate to the *Magritek Spinsolve* series of spectrometers. If the autosampler is to be used with a different model of spectrometer, several adjustments must be made. First, when drilling the holes into the aluminium plate, the three 5 mm holes must be replaced with an alternative pattern that allows screwing the baseplate to the instrument in a way that the centre hole is aligned with the sample hole of the spectrometer. Second, the “tube funnel” likely needs to be redesigned to match the correct sample height for the spectrometer.

**Fig. 3:**
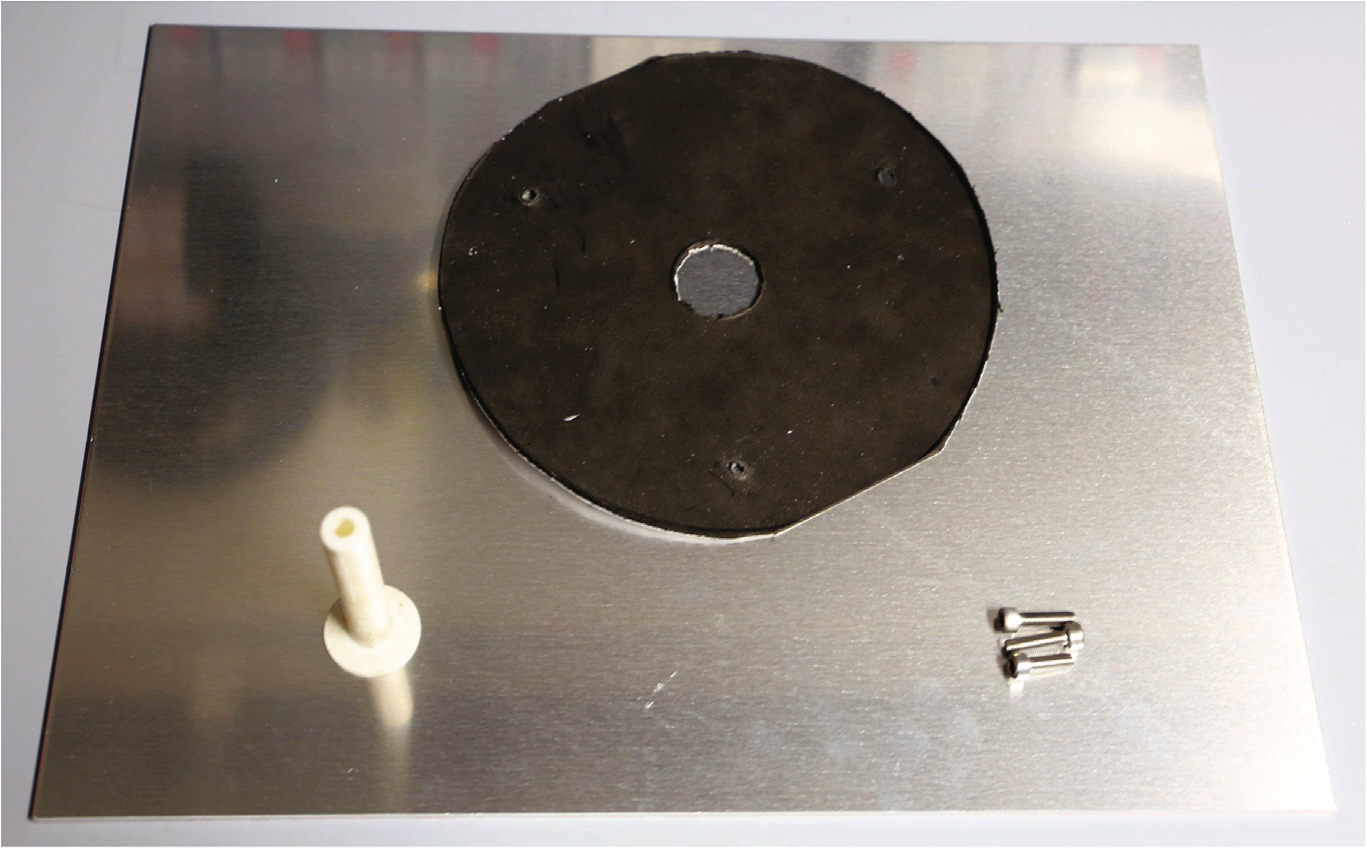
Parts required for the installation of the baseplate.

**Fig. 4:**
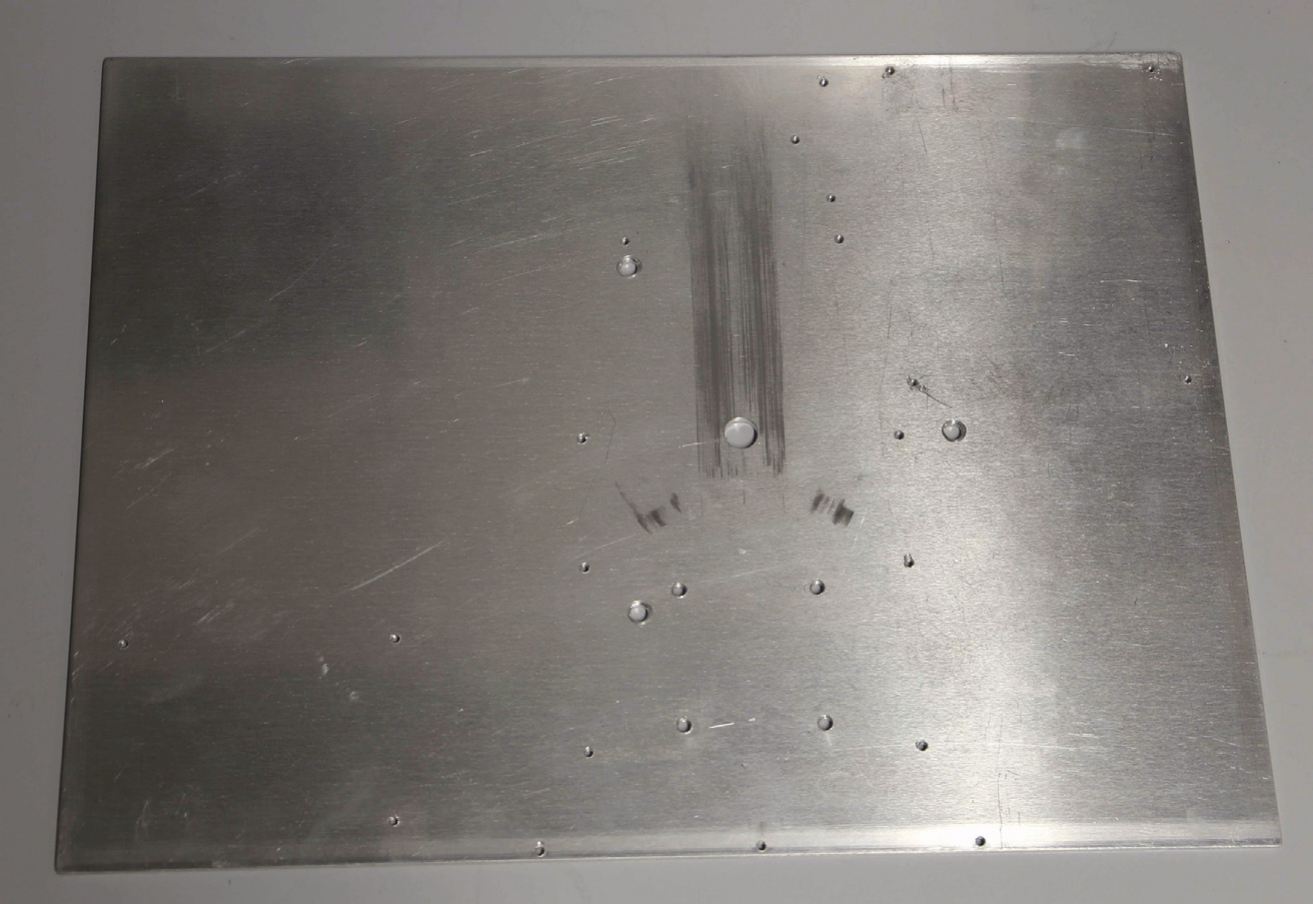
Aluminium plate with threaded holes.

An aluminium plate (300 × 400 × 3 mm) serves as the base for the autosampler. The parts required for the installation of the base plate are shown in Fig. 3, a list of parts is given in Table 1. To drill the mounting holes for the parts, print the template for drilling holes (Supplementary Information) on an A3 sheet, glue it to the aluminium plate, and use the centre punch to mark out the positions of the holes. Drill the holes with the corresponding core hole drill bits (2.5 mm for M3, 4.2 mm for M5 threads, Fig. 4). Then, cut the threads using taps. The three 5 mm holes are used to fasten the aluminium plate to the spectrometer with M5 bolts, and do not require any threading. Finally, the 12 mm hole in the centre of the aluminium plate is used to allow NMR samples to pass from the autosampler to the spectrometer.

**Table 1:** List of parts required for the installation of the baseplate.

Amount	Component	Type
1	Tube funnel	3D-printed part
1	Aluminium plate (40 cm × 30 cm × 3 mm)	Off-the-shelf part
1	Mousepad	Off-the-shelf part
3	M5 × 16 mm cylinder-head bolts (DIN 912)	Screws, Bolts, Nuts

**Fig. 5:**
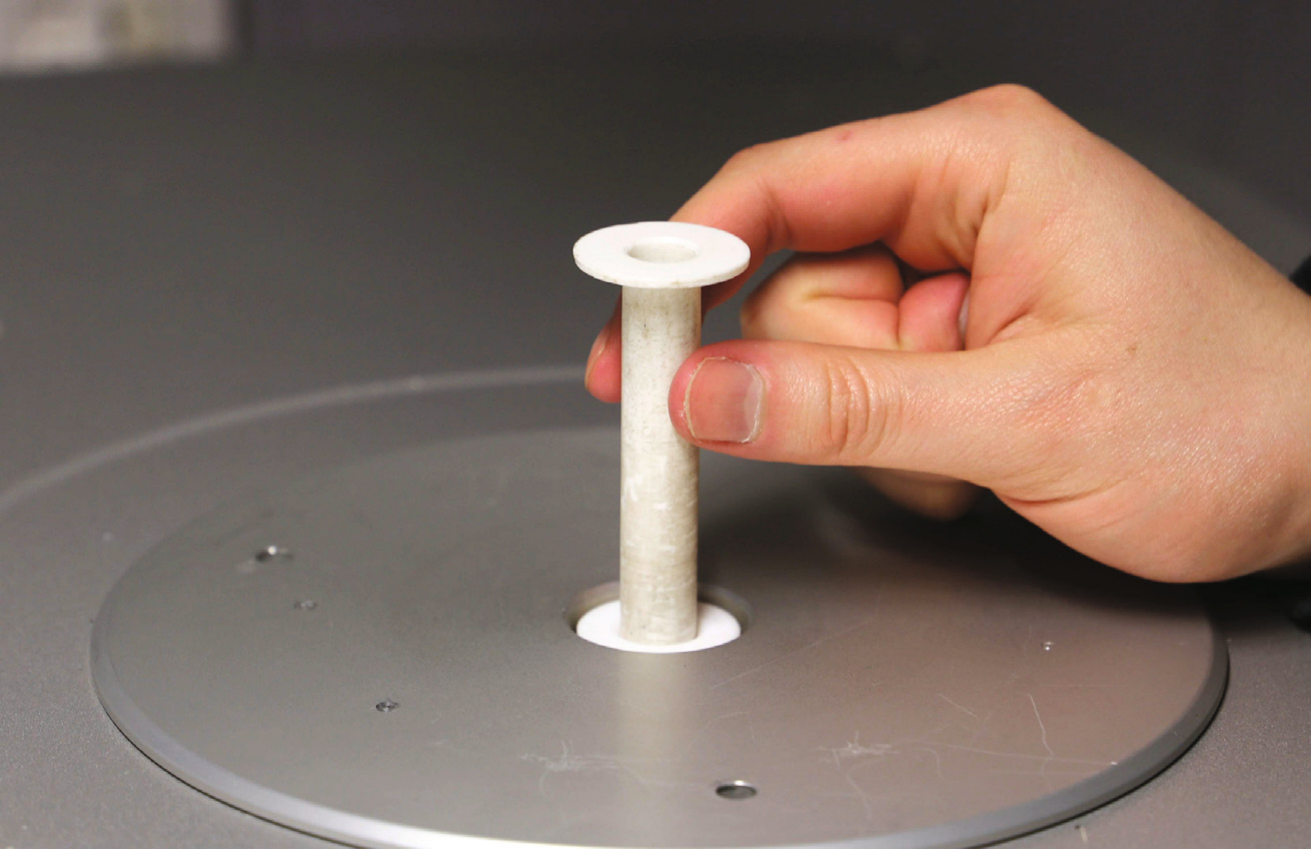
Inserting the tube funnel into the spectrometer.

To position the sample tubes at exactly the right height within the instrument, the “tube funnel” is required. After printing the tube funnel, make sure that the inner surface of the object is smooth to prevent samples from getting stuck inside of the machine. Smoothing the inner surface can be achieved using an adjustable hand reamer or by exposing the part to THF fumes in a closed container for several hours. When the tube funnel is sufficiently smoothed, insert it into the corresponding opening in the spectrometer (Fig. 5).

**Fig. 6:**
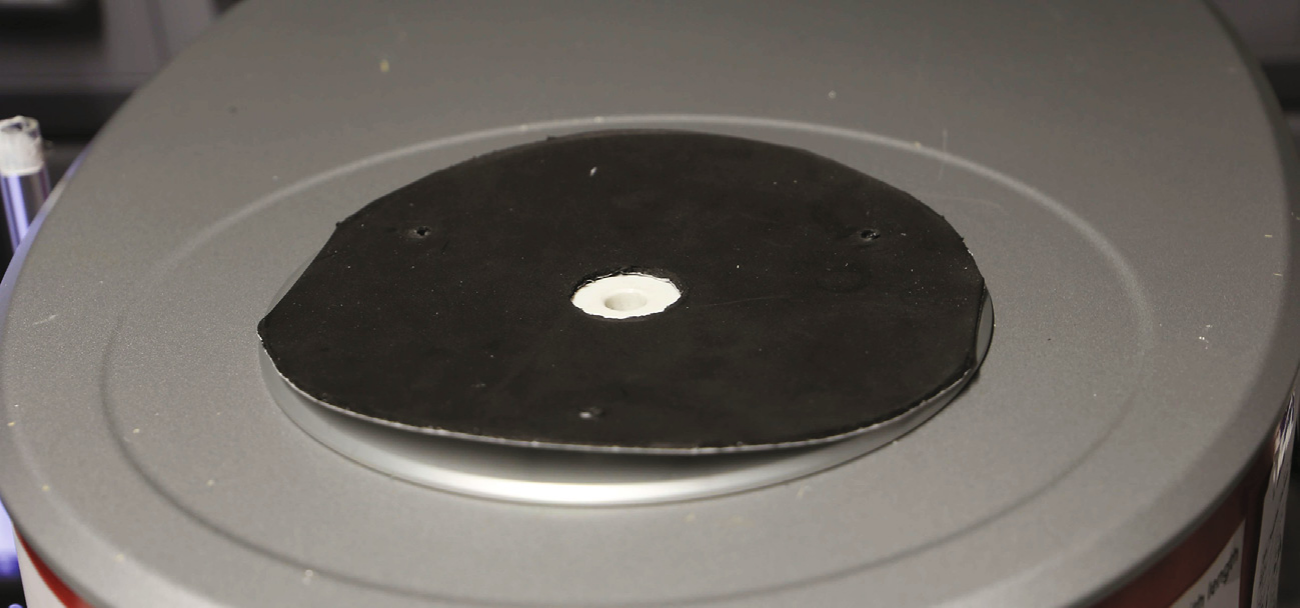
Placement of the rubber mat (mousepad) on top of the spectrometer.

Put a piece of rubber between the spectrometer and the baseplate, which contains a hole with a diameter of more than 12 mm and three holes for the mounting screws (Fig. 6). This will act as a gasket, as well as a buffer to allow some screws to slightly poke through the aluminium baseplate later on. As an example, a mousepad can be cut to shape and used for this purpose.

**Fig. 7:**
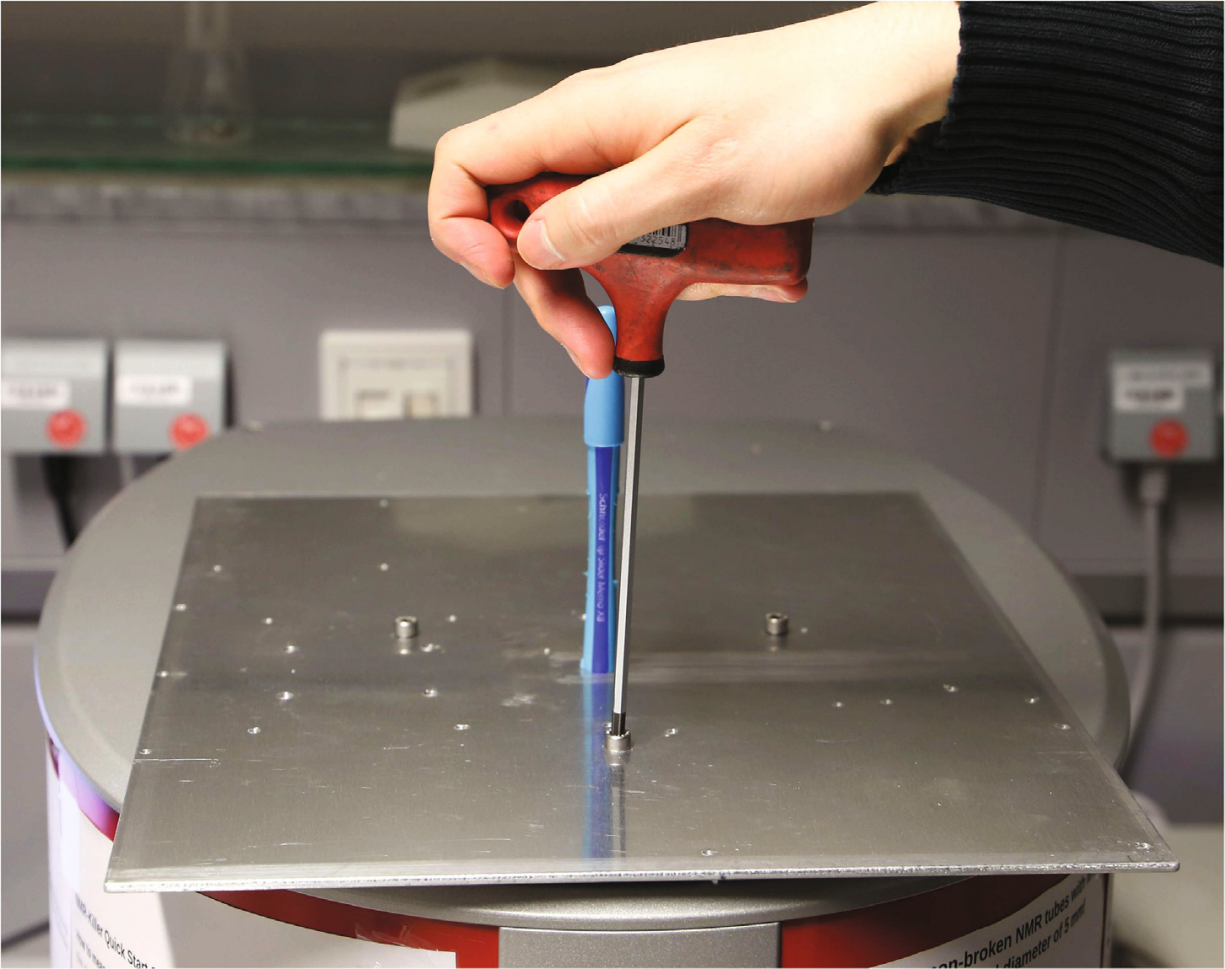
Mounting the aluminium baseplate on the spectrometer using a pen to ensure correct positioning.

Lightly insert the three bolts which connect the baseplate to the spectrometer. Make sure that the holes of the baseplate and the spectrometer are aligned. This is best achieved by inserting a rubber pen with a tapered end (e.g. *Schneider Slider Memo*) and moving it until a vertical position is achieved. With the pen still inserted, screw in the bolts as evenly as possible (Fig. 7).

#### 5.1.2 Insertion and ejection using compressed air

**Fig. 8:**
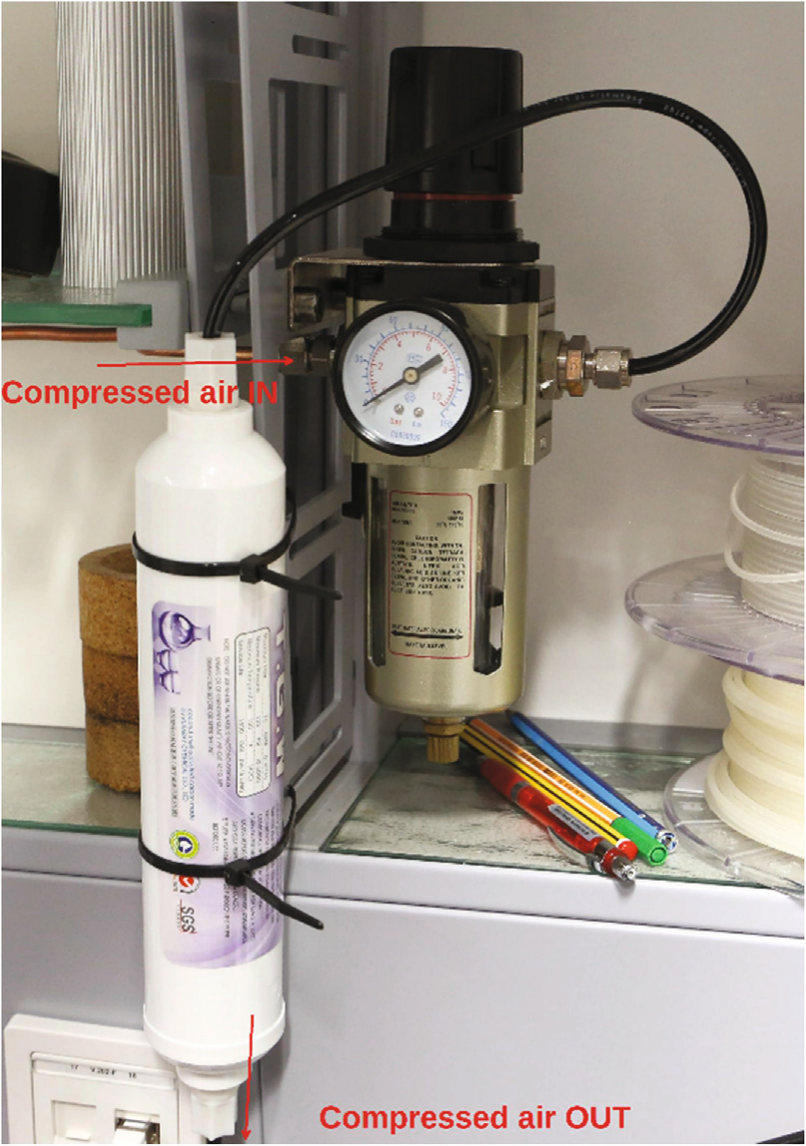
Regulation and purification of compressed air.

Samples are inserted into and ejected from the spectrometer using a stream of compressed air. The parts required for the assembly of the compressed air supply are listed in Table 2. In most laboratories, compressed air is available from a central supply; alternatively, a compressor can be used. Commonly, compressed air contains impurities, e.g. oil mist, which might cause issues if passed through the spectrometer. Therefore, the crude compressed air is first passed through a pressure regulator with a built-in oil trap, and then passed through an activated charcoal filter for further purification (Fig. 8). Assemble this system and, as a safety precaution, set the regulator to the lowest possible pressure.

**Table 2:** List of parts required for the assembly of the compressed air supply.

Amount	Component	Type
1	Plug	3D-printed part
1	Angle	3D-printed part
61 cm	PMMA or Polycarbonate pipe, OD = 15 mm, ID = 11 mm	Off-the-shelf part
2 m	Tubing for pressurized air (6 mm or 1/4″)	Off-the-shelf part
1	Pressure regulator with oil trap	Off-the-shelf part
1	Activated charcoal filter	Off-the-shelf part
1	O-Ring, 30 × 2.0 mm	Off-the-shelf part
–	PTFE tape	Off-the-shelf part

**Fig. 9:**
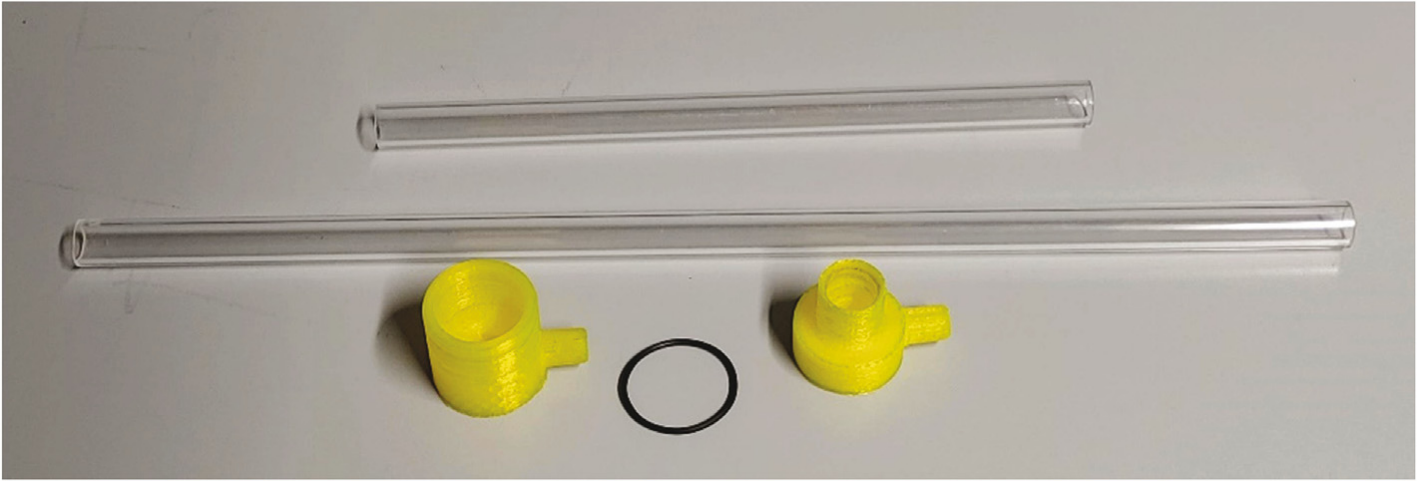
The parts used to direct compressed air into the spectrometer.

**Fig. 10:**
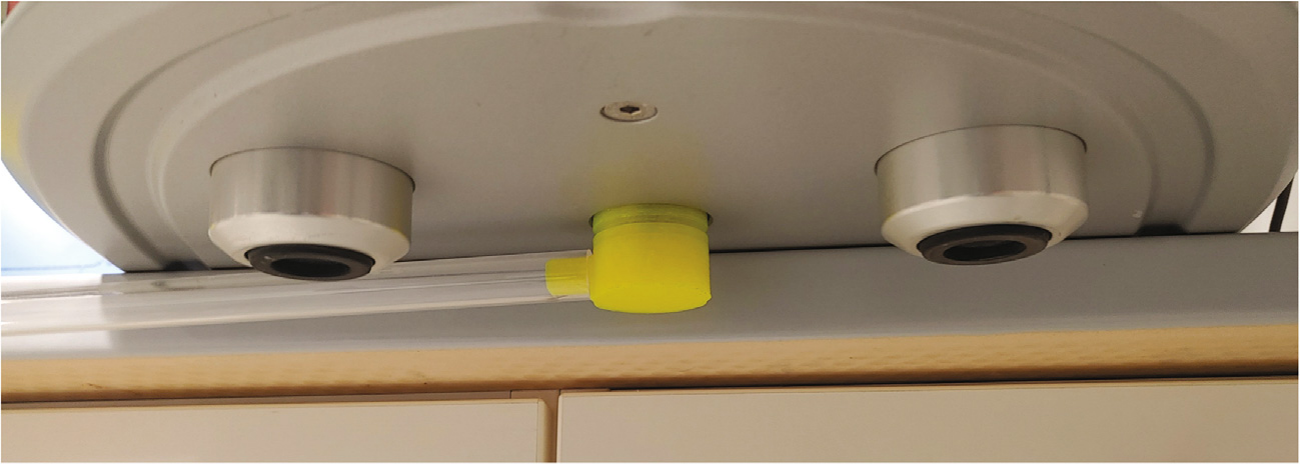
Insertion of the Plug into the spectrometer.

Subsequently, the purified stream of compressed air is passed through a 3D-printed flow regulator (see Section 5.1.3, “*Flow regulator”*) and two plastic tubes. The parts “Plug” and “Angle” are responsible to guide the compressed air into the spectrometer (Fig. 9). To attach the Plug to the spectrometer, add an O-ring (30 × 2.0 mm), lift up the spectrometer and insert the Plug into the hole at the bottom of the spectrometer (Fig. 10).

**Fig. 11:**
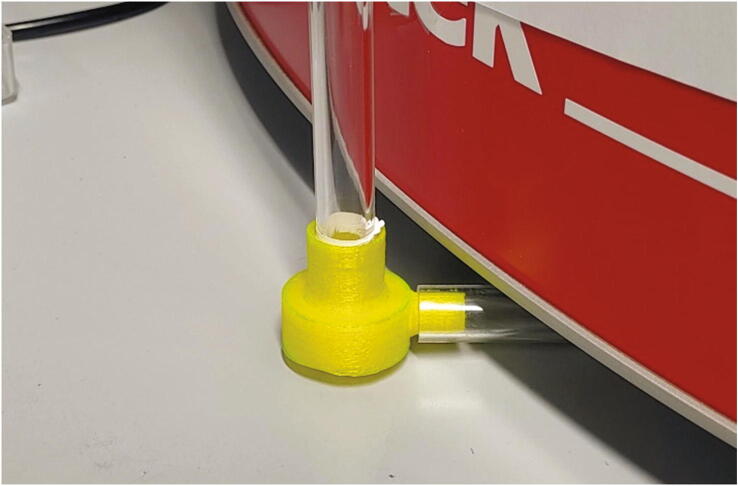
Angle with plastic tubes.

Two plastic pipes (PMMA, or polycarbonate for better chemical resistance) are used to guide compressed air into the spectrometer. From your plastic pipes, cut off one 21 cm for the horizontal and one 40 cm piece for the vertical connection. The 21 cm piece is connected to the Plug and Angle connectors, while the long 40 cm pipe is pushed into the Angle. To prevent air leaks, it may be necessary to tighten the joints with PTFE tape (See Fig. 11).

#### 5.1.3 Flow regulator

**Fig. 12:**
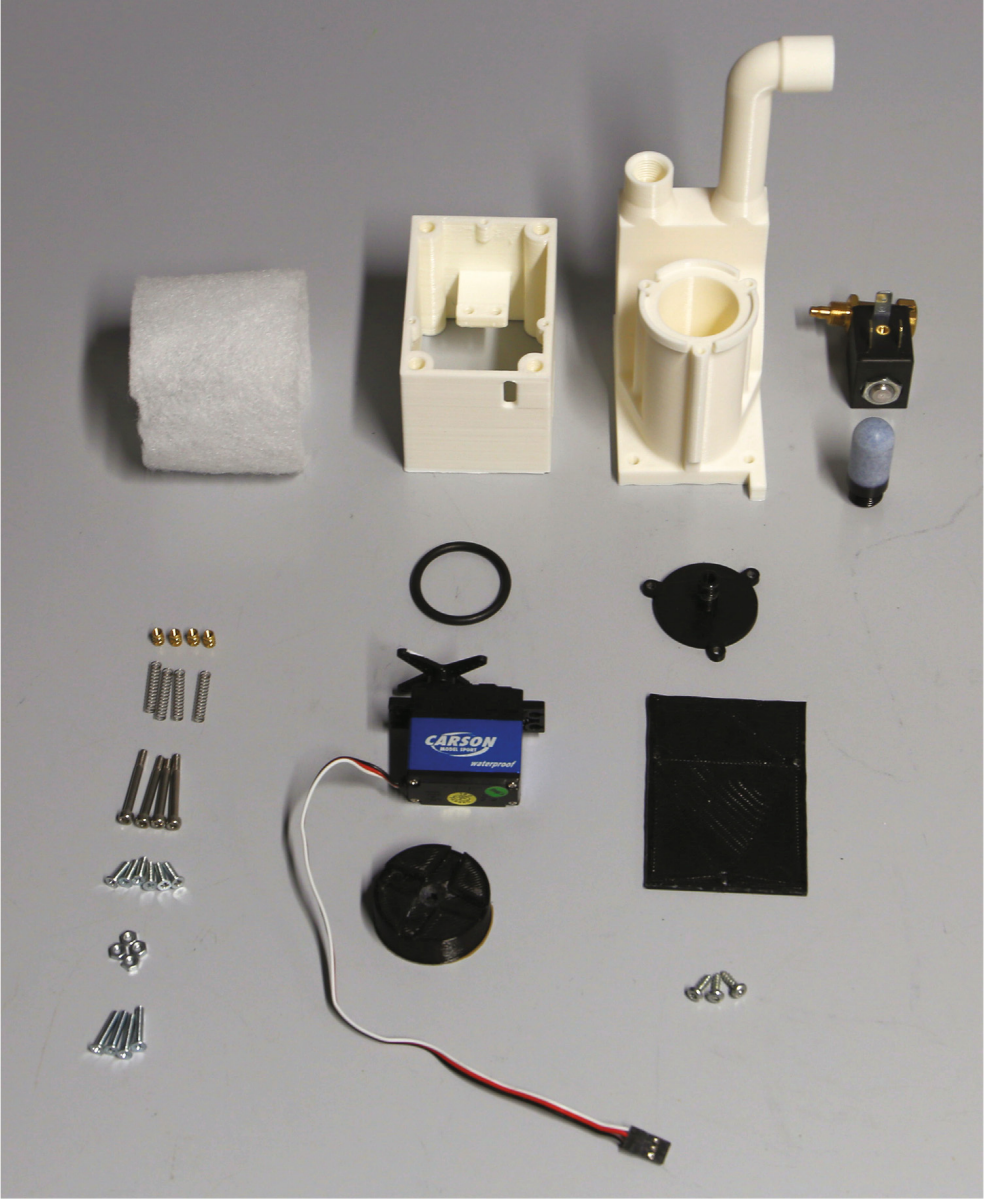
All parts for the flow regulator, excluding adhesive PTFE foil.

The parts required for the assembly of the flow regulator are shown in Fig. 12, a list of parts is given in Table 3. The flow regulator has two components: a solenoid valve and a servo-operated connector directing the gasflow. If the gasflow was only switched on and off by operating the solenoid valve, the samples would rapidly shoot out of the spectrometer, hit the sensor at high speed, and possibly break. Thus, the groove of the flow regulator’s “inner part” connects the pressurized air with an outlet tube in the resting state, so that all of the air is initially vented when the magnetic valve is switched on (Fig. 14). Then, the servo motor slowly turns the inner part, decreasing the fraction of air which is vented and increasing the amount of air passing through the spectrometer, thus gently raising the tube without smashing it. When the fraction of air passing through the spectrometer is decreased, the tube is gently lowered, and the solenoid valve is switched off only after the regulator has reached resting state with all of the air being diverted to the vent.

**Table 3:** List of parts required for the assembly of the compressed air supply.

Amount	Component	Type
1	Flow regulator – Inner part	3D-printed part
1	Flow regulator – Outer part	3D-printed part
1	Flow regulator – Lid	3D-printed part
1	Flow regulator – Casing	3D-printed part
1	Flow regulator – Backplate	3D-printed part
1	Adhesive PTFE foil	Off-the-shelf part
1	Screw-on silencer (1/4″ thread)	Off-the-shelf part
1	Solenoid valve, MARCO EV130 (12 V version)	Off-the-shelf part
1	Servo motor, Carson CS-13	Off-the-shelf part
–	Filter fleece	Off-the-shelf part
1	O-Ring, 30 × 3.5 mm	Off-the-shelf part
4	Compression springs, VD-052F	Off-the-shelf part
4	M3 threaded heat inserts	Screws, Bolts, Nuts
4	M3 × 30 mm cylinder-head bolts (DIN 912)	Screws, Bolts, Nuts
6	M3 × 10 mm countersunk bolts (DIN 965)	Screws, Bolts, Nuts
4	M3 × 16 mm countersunk bolts (DIN 965)	Screws, Bolts, Nuts
4	M3 nuts	Screws, Bolts, Nuts
3	3 × 10 mm flat-head wood screws	Screws, Bolts, Nuts

The “inner part” and “outer part” of the flow regulator are pushed towards each other by springs. This not only ensures smooth turning at minimal drag, it is also a safety mechanism to prevent build-up of pressure inside of the pneumatic system if it gets clogged somewhere. In such an event, the parts separate, releasing the overpressure. The unpleasant hissing noise of the gasflow is efficiently repressed by a screw-on silencer in combination with an internal filter fleece muffler.

**Fig. 13:**
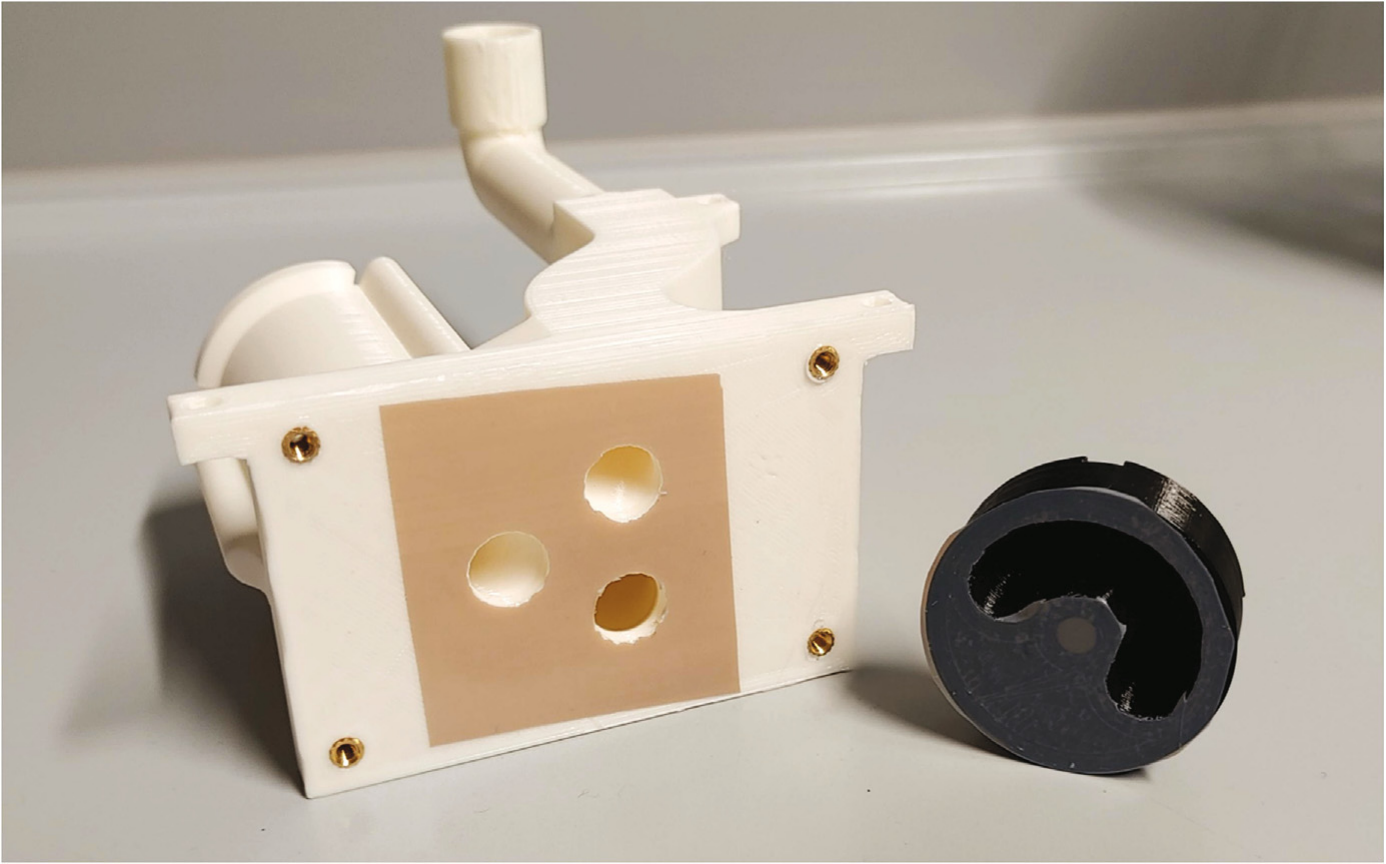
Inner and outer parts of the flow regulator with heated inserts & adhesive PTFE foil.

To assemble the flow regulator, start by inserting M3 heated inserts to the flow regulator’s “outer part”. Then, add adhesive PTFE foil to the corresponding sides of inner and outer parts (Fig. 13). The PTFE foil serves as a gasket and decreases friction between the two parts.

**Fig. 14:**
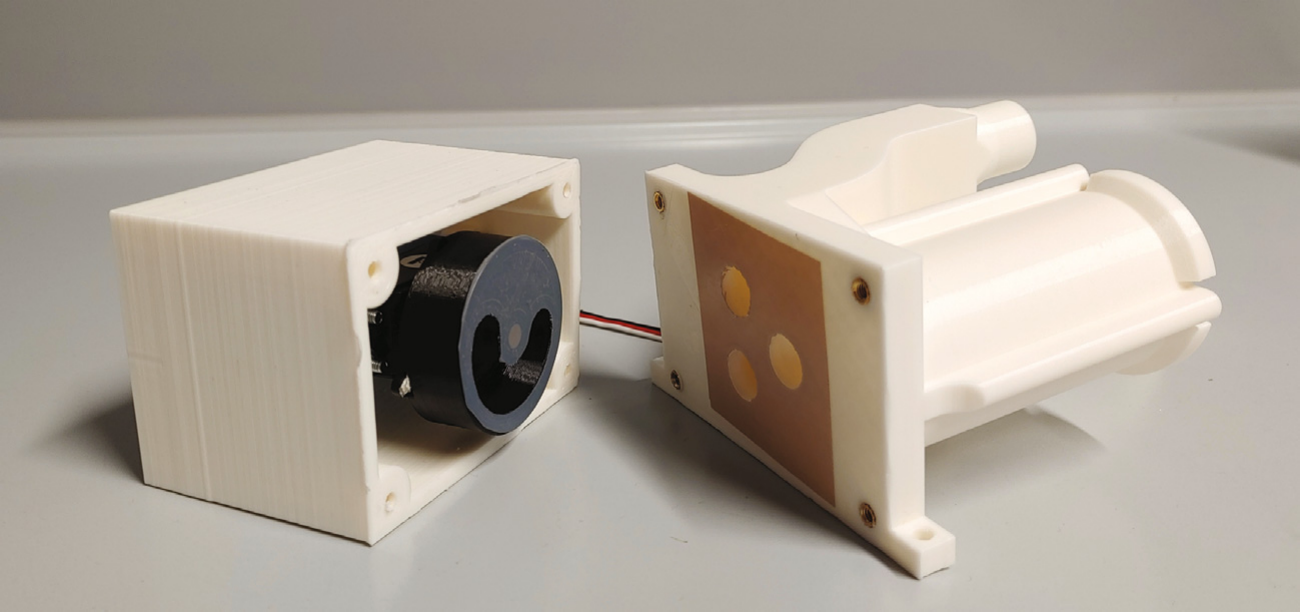
The air intake is connected to the spectrometer.

**Fig. 15:**
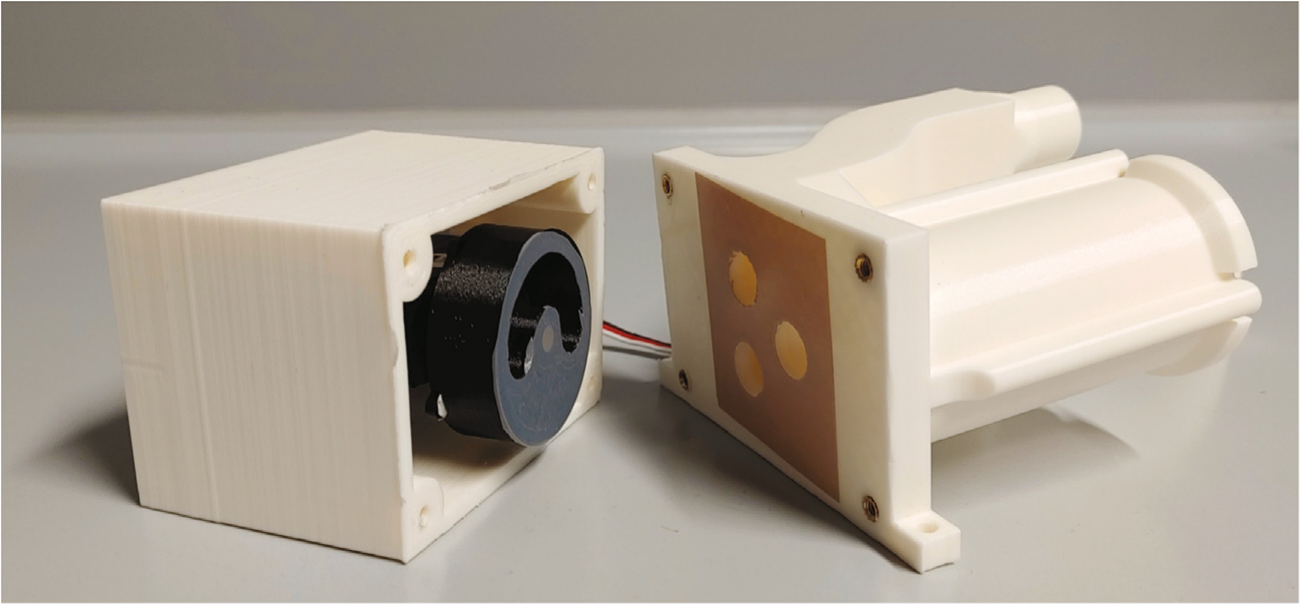
The air intake is connected to the vent hole.

Mount the servo motor in the flow regulator’s casing using M3 × 10 mm bolts and nuts, add the cross-shaped drive arm to the servo and mount the inner part of the flow regulator on top. Turn the servo as far as possible, and make sure that the inner part only connects the air intake with one of the holes (Fig. 14). Turning the servo should slowly disconnect the air intake from one hole, while simultaneously connecting the air intake to the other hole, i.e. the inner part must be able turn clockwise from the position shown in Fig. 14 to the position shown in Fig. 15.

**Fig. 16:**
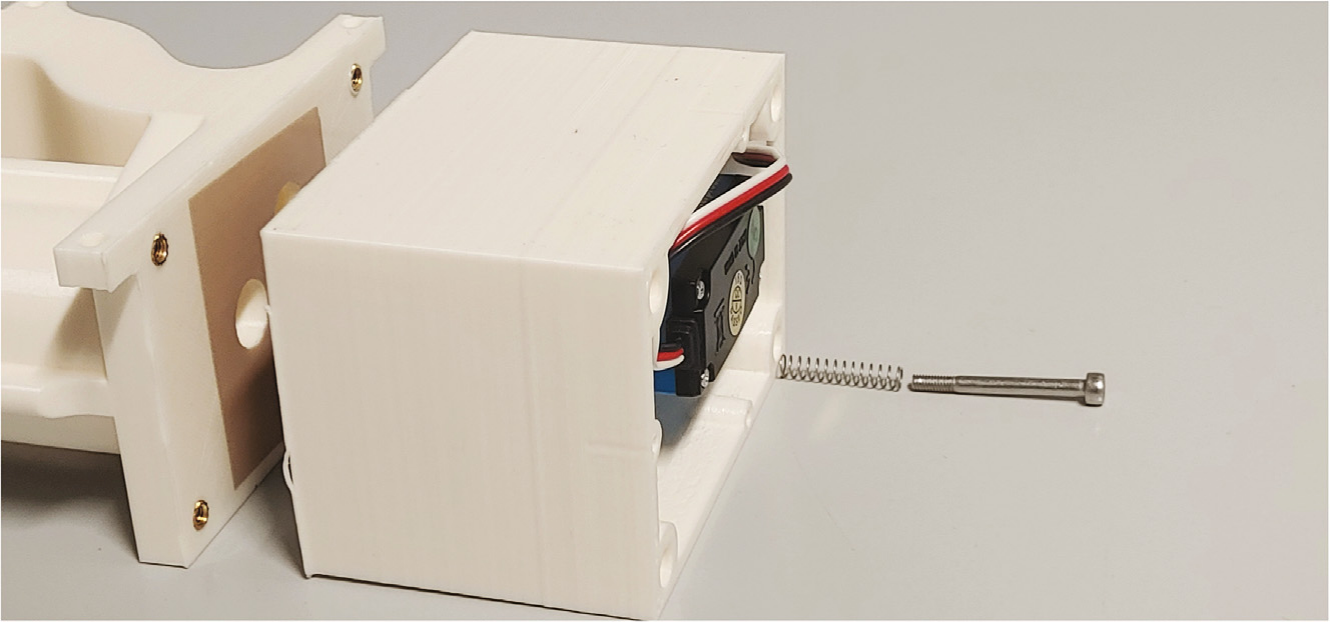
Adding the spring and screw to the casing of the flow regulator.

Finally, add compressing springs onto four M3x30 mm screws and add them to the four holes in the casing. Now, use an Allen key to screw them into the M3 heated inserts of the outer part of the flow regulator (Fig. 16). Make sure that the springs are sufficiently compressed to ensure a tight fit but will immediately give way in the case of an internal overpressure. Test if the parts can be pulled apart for at least 2 mm by hand applying moderate force.

**Fig. 17:**
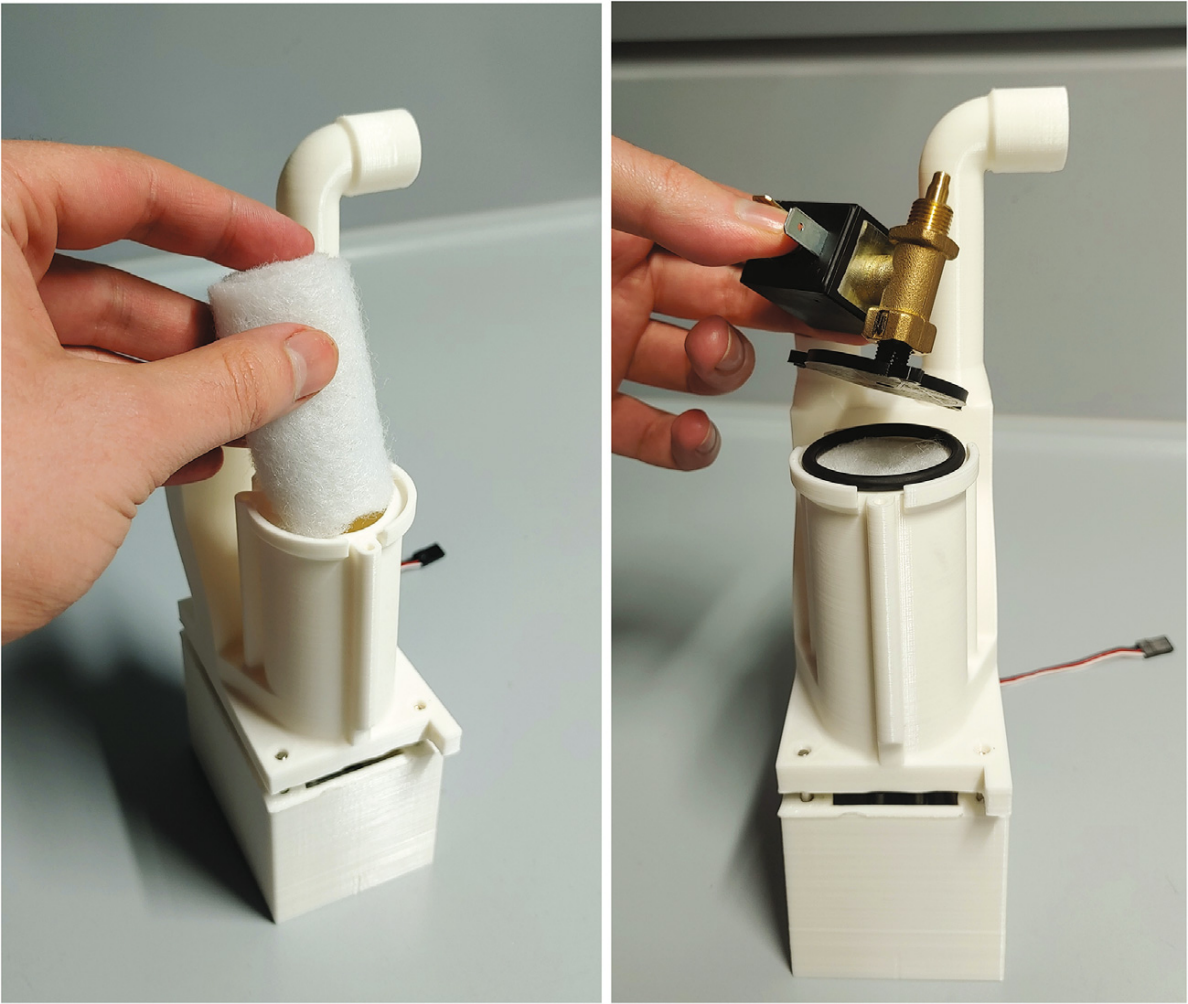
Assembly of the muffler and magnetic valve.

Next, roll up some filter fleece and add it into the chamber of the outer part of the flow regulator as a muffler. Add an O-ring (30 × 3.5 mm) in front of the big chamber. Connect the magnetic valve to the compressed air supply (Section 5.1.2, “*Insertion and ejection using compressed air*”) and the flow regulator’s lid. Then, use three M3 × 10 mm screws to close the chamber (Fig. 17). To further reduce noise, screw the screw-on silencer into the venting tube of the flow regulator.

**Fig. 18:**
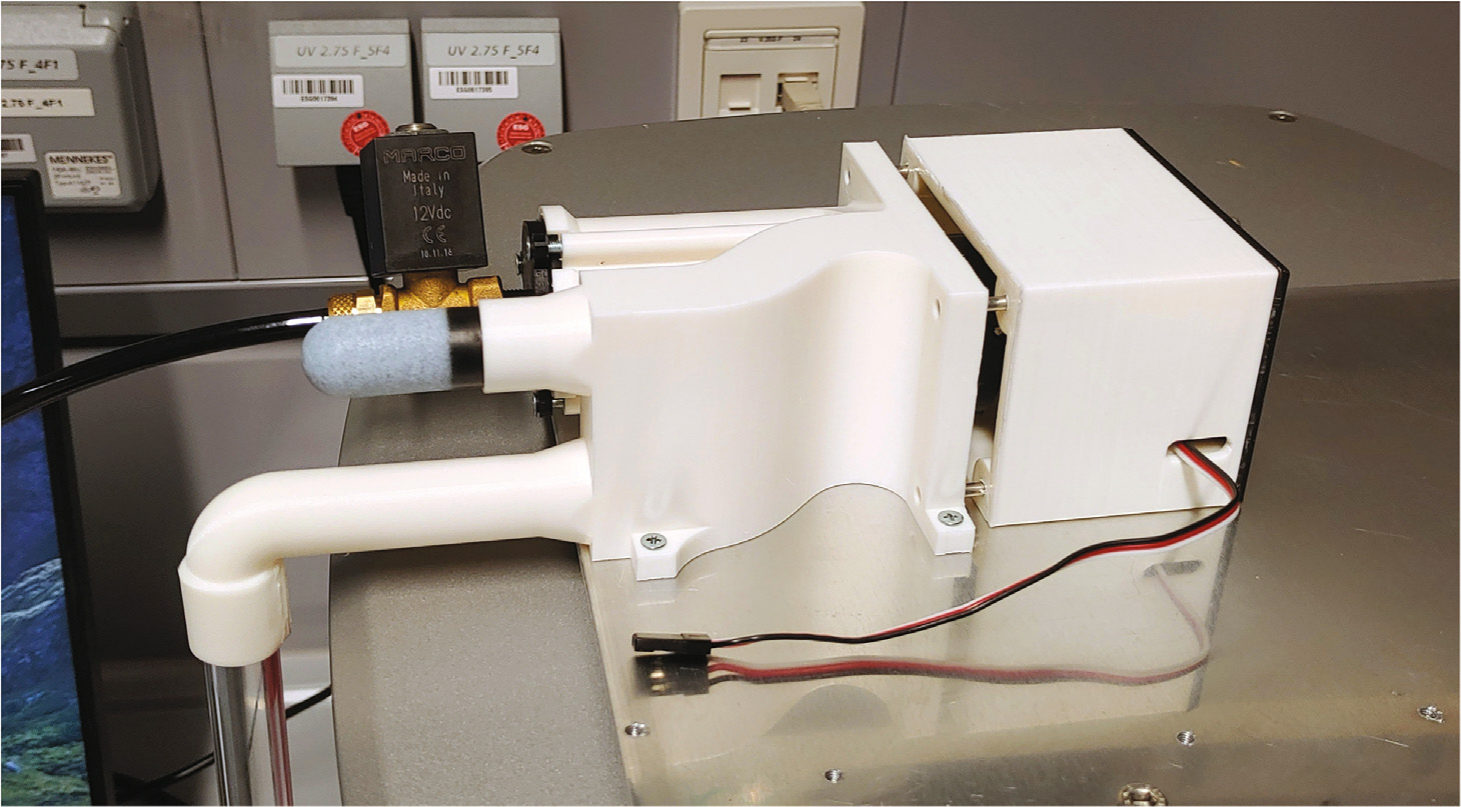
Fully assembled flow regulator.

Finally, add some PTFE tape to the plastic tube connecting to the bottom of the spectrometer, and push the flow regulator onto the tube. Then, use three M3 × 10 mm screws to mount the flow regulator to the aluminium baseplate, and close the assembly by screwing the backplate onto the casing using 3 × 10 mm wood screws (Fig. 18).

#### 5.1.4 Main motor holder, tower, and servo holder

**Fig. 19:**
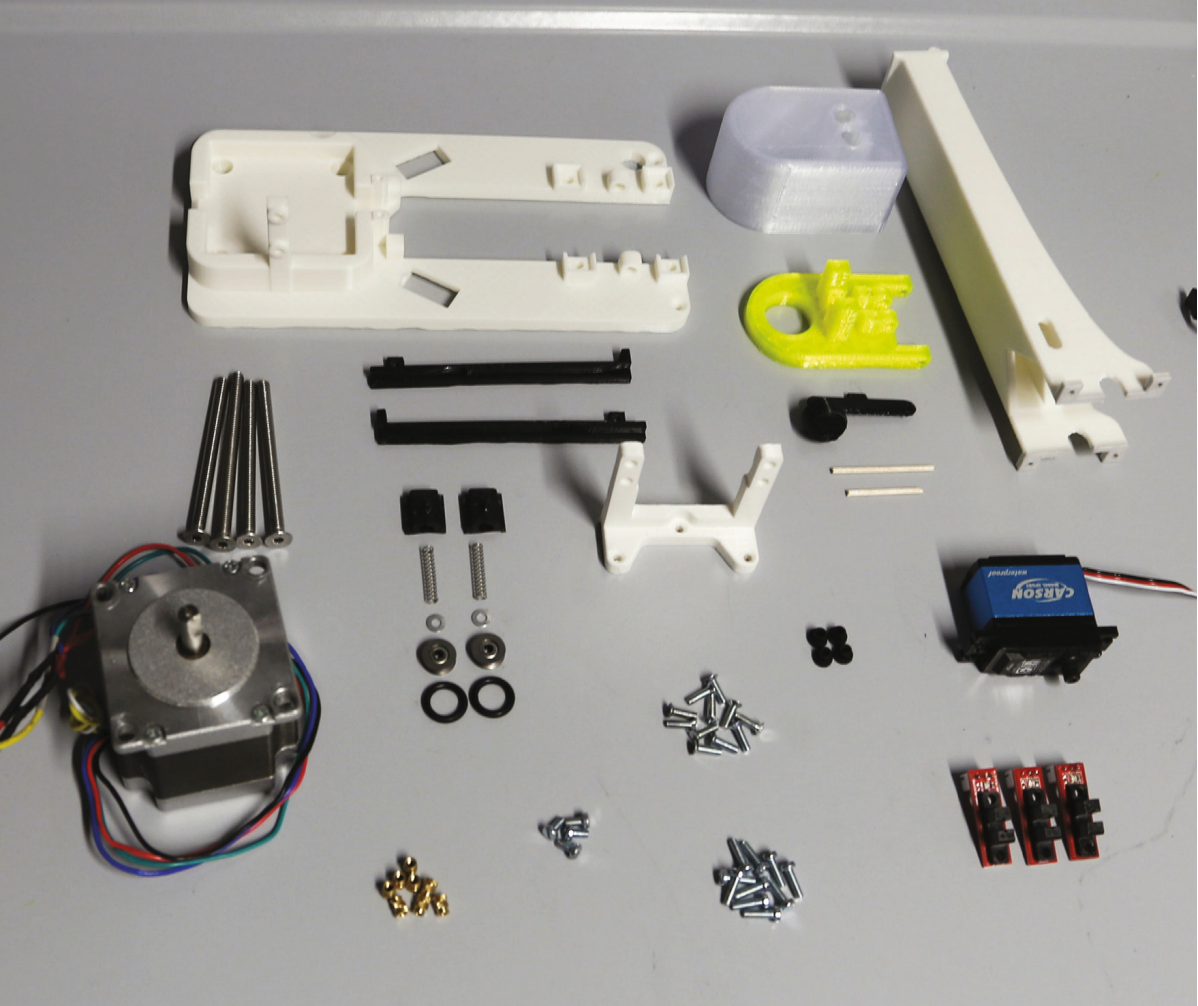
All parts required for this step, excluding wires.

**Fig. 20:**
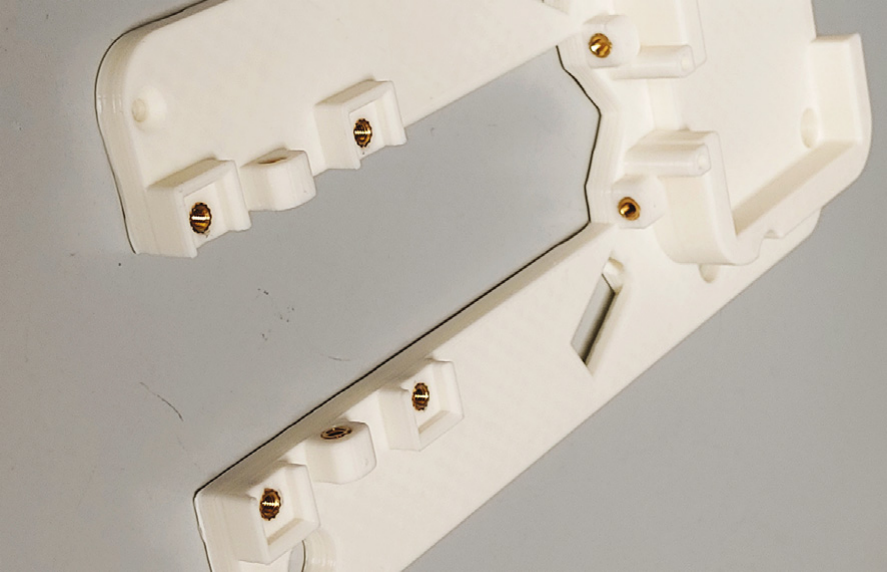
Threaded heat inserts of the main motor holder.

The main motor holder is the central part of the autosampler, holding in place the stepper motor and most mechanical components. The parts required for the assembly of the main motor holder are shown in Fig. 19 a list of parts is given in Table 4. For its assembly, melt the M3 threaded inserts into the eight holes with the help of a soldering iron as shown in Fig. 20. Next, mount the optical switches to the main motor holder using M3x10 mm cylinder-head screws, and mount the “guide rails” to the main motor holder using M3 × 10 mm cylinder-head screws.

**Table 4:** List of parts required for the assembly of the main motor holder, tower, and servo holder.

Amount	Component	Type
1	Main motor holder	3D-printed part
1	Guide rail (left)	3D-printed part
1	Guide rail (right)	3D-printed part
2	Spring blocks	3D-printed part
1	Tower – Shelf	3D-printed part
1	Tower – Lid	3D-printed part
1	Tower – Endstop	3D-printed part
1	Tower – Body	3D-printed part
1	Servo holder	3D-printed part
1	Stepper motor NEMA 23 × 56 mm, 4-wire	Off-the-shelf part
1	Servo motor, Carson CS-13	Off-the-shelf part
3	Optical endstop switch modules, TCST2103	Off-the-shelf part
2	Compression springs, VD-052F	Off-the-shelf part
2	Ball bearings, 3 × 12 × 4 mm with V-groove	Off-the-shelf part
2	O-Rings, 10 × 2.5 mm	Off-the-shelf part
1	Wooden stick	Off-the-shelf part
8	M3 threaded heat inserts	Screws, Bolts, Nuts
4	M3 × 6 mm cylinder-head bolts (DIN 84)	Screws, Bolts, Nuts
10	M3 × 10 mm cylinder-head bolts (DIN 84)	Screws, Bolts, Nuts
11	M3 × 10 mm countersunk bolts (DIN 965)	Screws, Bolts, Nuts
4	M5 × 70 mm countersunk bolts (DIN 965)	Screws, Bolts, Nuts
2	M3 washers	Screws, Bolts, Nuts

**Fig. 21:**
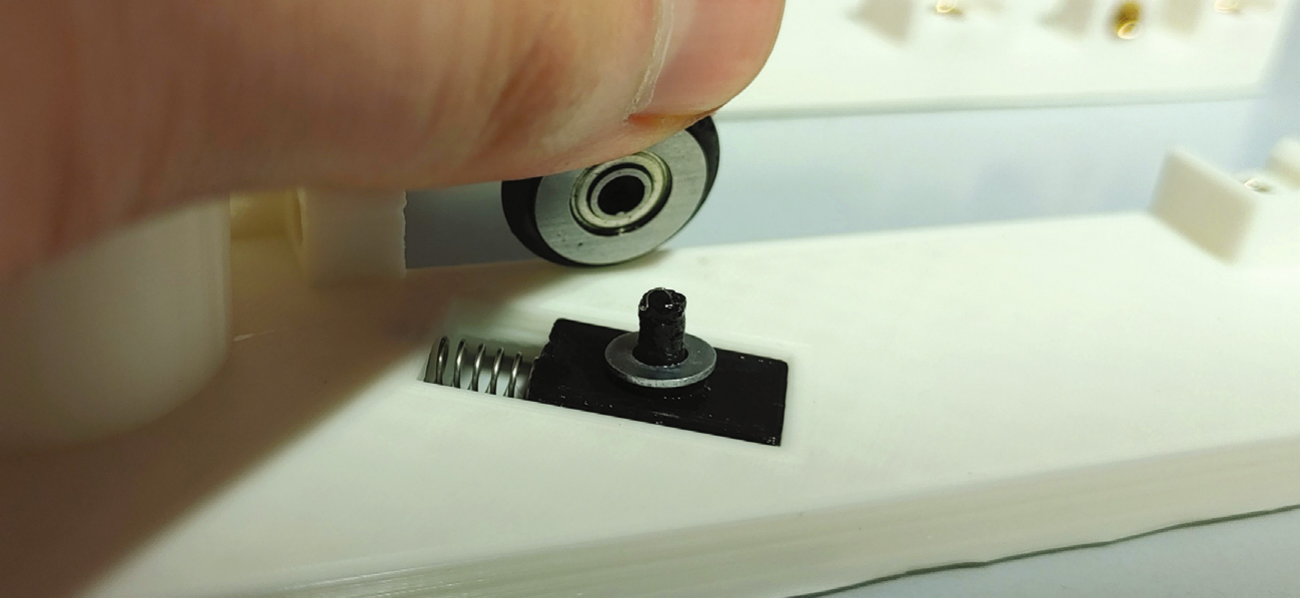
Assembly of a spring block.

The “spring blocks” ensure that the tubes of the rotor are aligned with the hole in the aluminium baseplate (see Sections 5.1.1, “*Aluminium baseplate with funnel*” and 5.1.7, “*Rotor*”). To assemble the spring block, add compression springs into the corresponding holes, then place the blocks into the corresponding slots of the main motor holder. Add a washer on top of the spring block. Finally, fit the O-rings (10 × 2.5 mm) onto the ball bearings and plug the ball bearings onto the spring blocks (Fig. 21).

**Fig. 22:**
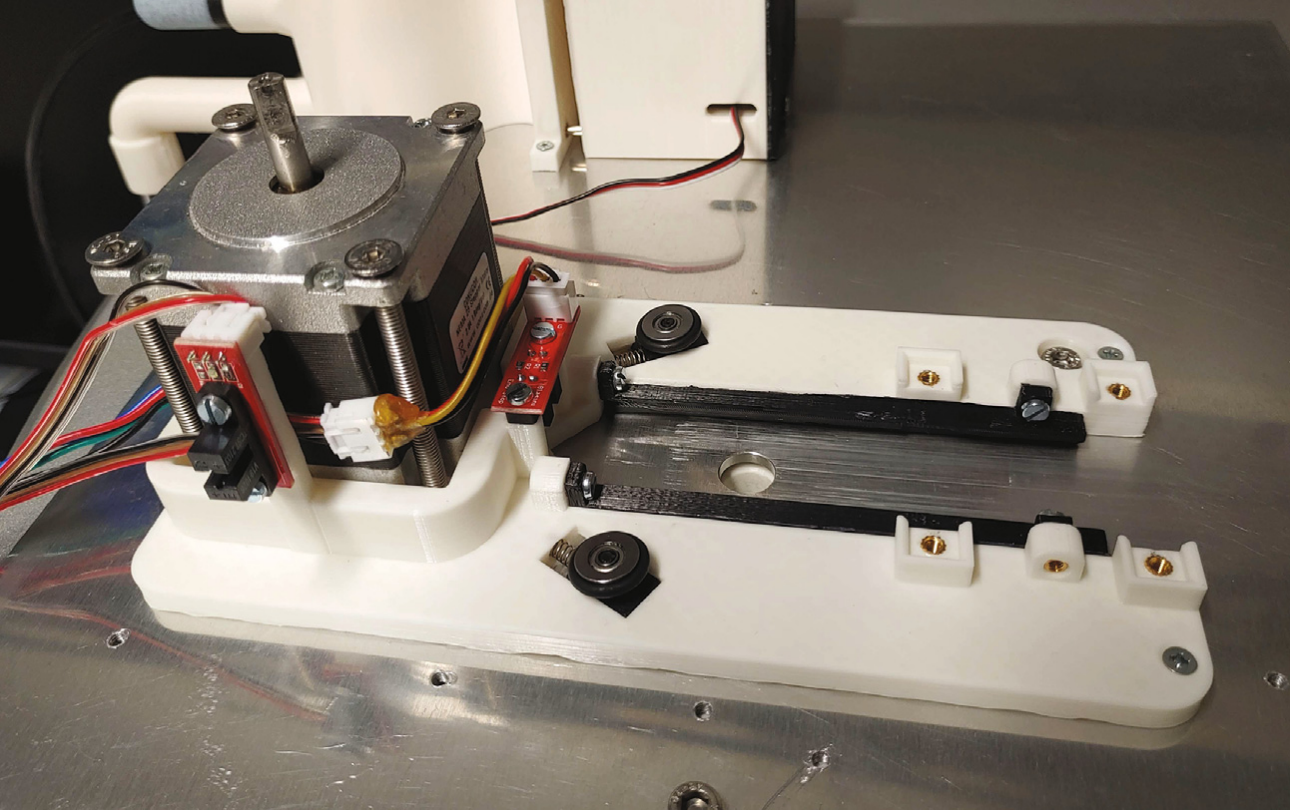
The assembled and mounted main motor holder.

Now, the main motor holder can be mounted onto the aluminium baseplate. Place the stepper motor into its slot and screw it in place using four M5x70 mm bolts. Then, add and fasten two M3x10 mm countersunk bolts at the other end of the main motor holder (Fig. 22).

**Fig. 23:**
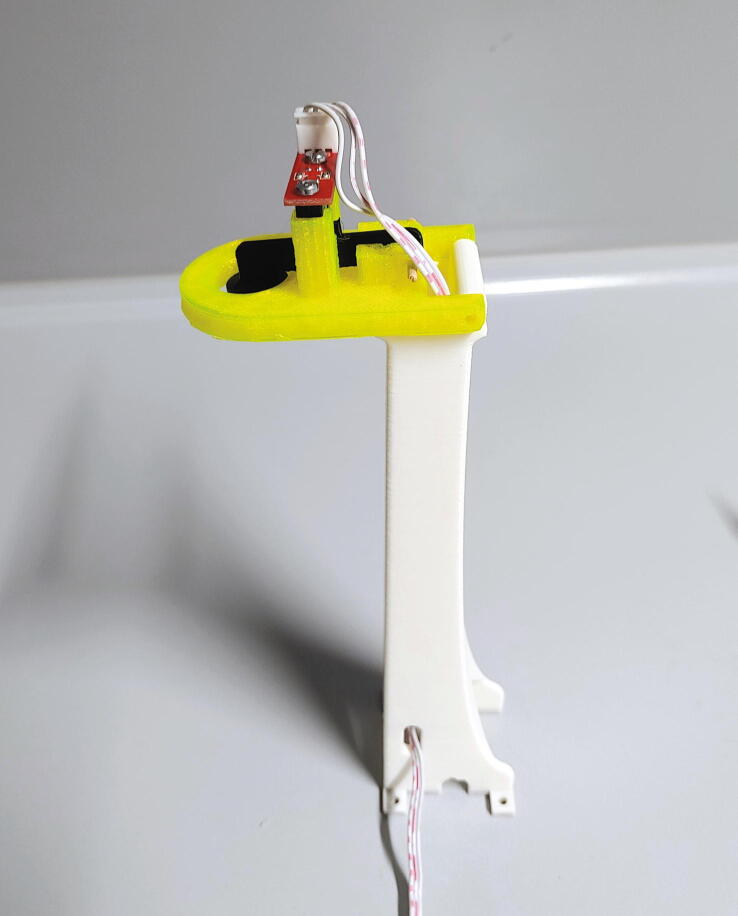
The tower after assembly.

The next part to assemble is the “tower”. The tower contains a switch which detects whether a sample is currently being lifted up above the centre hole by compressed air. To assemble this part, first mount the optical switch on the “tower shelf” using M3x10 mm cylinder-head screws. Then, connect the part “tower endstop” to the tower’s shelf via a pivot. As an example, part of a wooden cotton swab can be used. The shelf is then connected to the “tower body”, also using a pivot, and the wires of the optical switch are passed through the hole in the tower body. The ability to fold back the entire upper part of the tower simplifies maintenance and troubleshooting: the currently measured position is easily accessible and the rotor can be removed without unscrewing the tower. Mount the tower to the motor holder using four M3 × 10 mm cylinder-head bolts (Fig. 23).

**Fig. 24:**
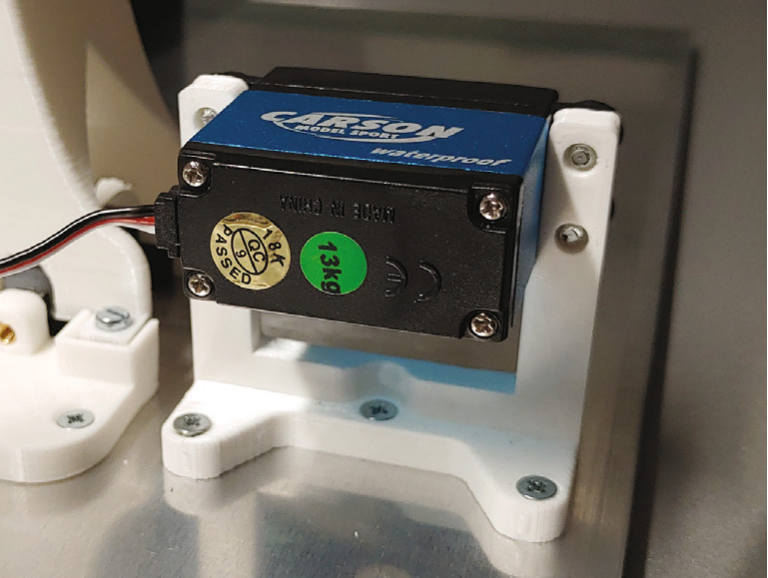
The servo holder and the slider servo.

Finally, mount the “servo holder” on the aluminium baseplate using three M3 × 10 mm countersunk bolts, and mount the servo motor using four M3 × 10 mm countersunk bolts (Fig. 24).

#### 5.1.5 Electronics

**Fig. 25:**
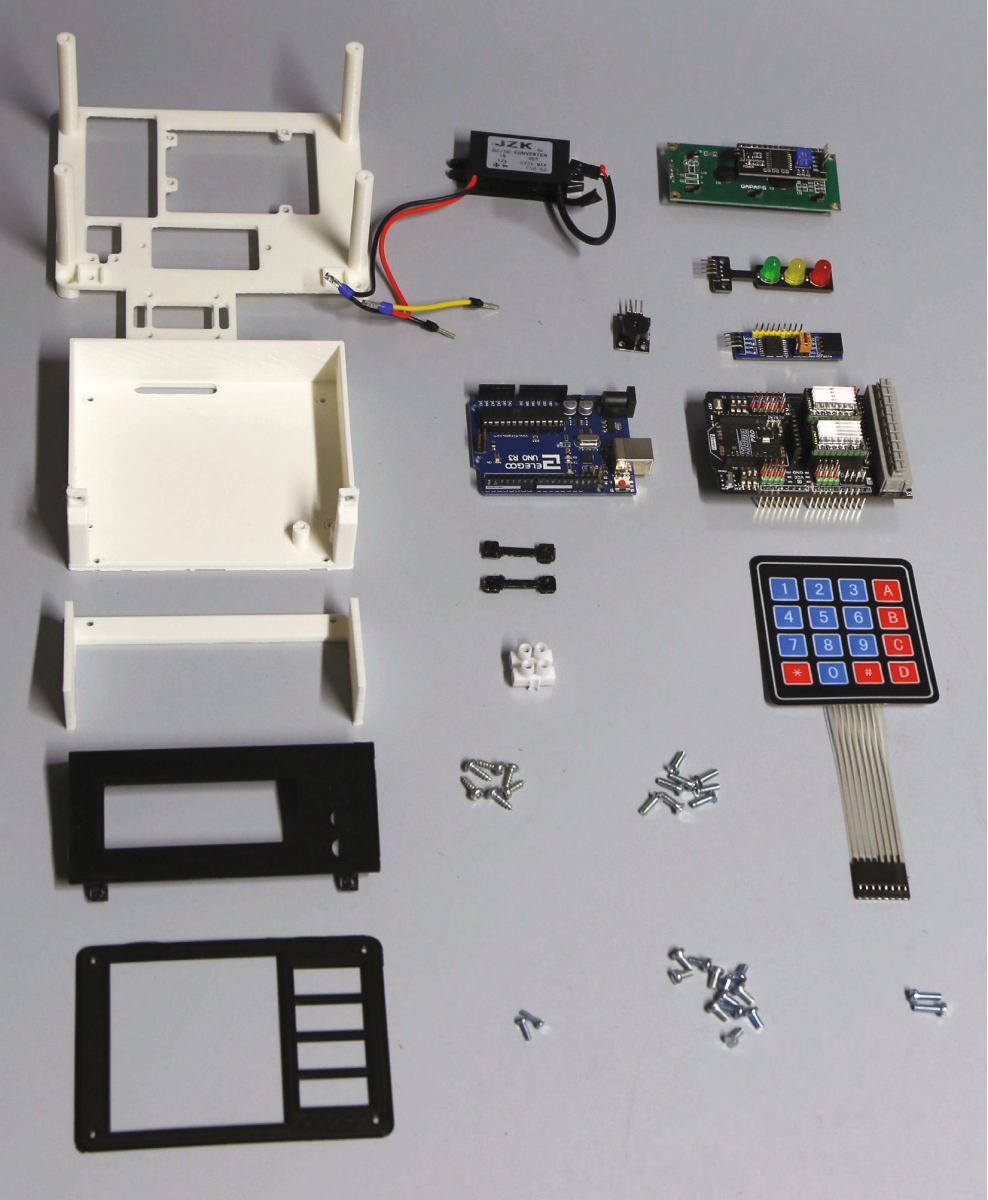
Parts required for the assembly of the electronics, excluding wires and electronics lid.

The parts required for the assembly of the electronics are shown in Fig. 25, a list of parts is given in Table 5.

**Table 5:** List of parts required for the assembly of the electronics.

Amount	Component	Type
1	Electronics holder – Base plate	3D-printed part
2	Electronics holder – Clip for shift register	3D-printed part
1	Electronics holder – Keypad holder	3D-printed part
1	Electronics holder – Display holder	3D-printed part
1	Electronics holder – Display holder side panel	3D-printed part
1	Electronics holder – Mounting bracket	3D-printed part
1	Electronics Lid	3D-printed part
1	Sliding ring	3D-printed part
1	Cable duct	3D-printed part
1	Limiter block	3D-printed part
1	Arduino Uno R3	Off-the-shelf part
1	Motor shield, DFRobot DRV8825	Off-the-shelf part
1	Shift register module, PCF8574T	Off-the-shelf part
1	Active buzzer module	Off-the-shelf part
1	LED traffic light module	Off-the-shelf part
1	LCD Display, LCM1602 with I2C bus	Off-the-shelf part
1	Matrix keypad (4x4)	Off-the-shelf part
1	Diode (12 V)	Off-the-shelf part
2	Lustre terminals	Off-the-shelf part
1	DC/DC Converter (12 V to 5 V), 15 W	Off-the-shelf part
1	DC plug 2.5 × 5.5 mm	Off-the-shelf part
1	Ball bearing, 3 × 12 × 4 mm with V-groove	Off-the-shelf part
1	O-Ring, 10 × 2.5 mm	Off-the-shelf part
10	Electric wire ferrules	Off-the-shelf part
1	Heat-shrink tube set	Off-the-shelf part
2	Blade receptacles, 6.3 mm	Off-the-shelf part
1	Set of Dupont Jumper Connectors (optional)	Off-the-shelf part
1	M3 threaded heat insert	Screws, Bolts, Nuts
2	M2 × 6 mm cylinder-head bolts (DIN 84)	Screws, Bolts, Nuts
10	3 × 10 mm flat-head wood screws	Screws, Bolts, Nuts
12	M3 × 6 mm cylinder-head bolts (DIN 84)	Screws, Bolts, Nuts
2	M3 × 10 mm cylinder-head bolts (DIN 84)	Screws, Bolts, Nuts
16	M3 × 10 mm countersunk bolts (DIN 965)	Screws, Bolts, Nuts
2	M3 nuts	Screws, Bolts, Nuts
1	M3 washer	Screws, Bolts, Nuts
1	M3 threaded rod	Screws, Bolts, Nuts

**Fig. 26:**
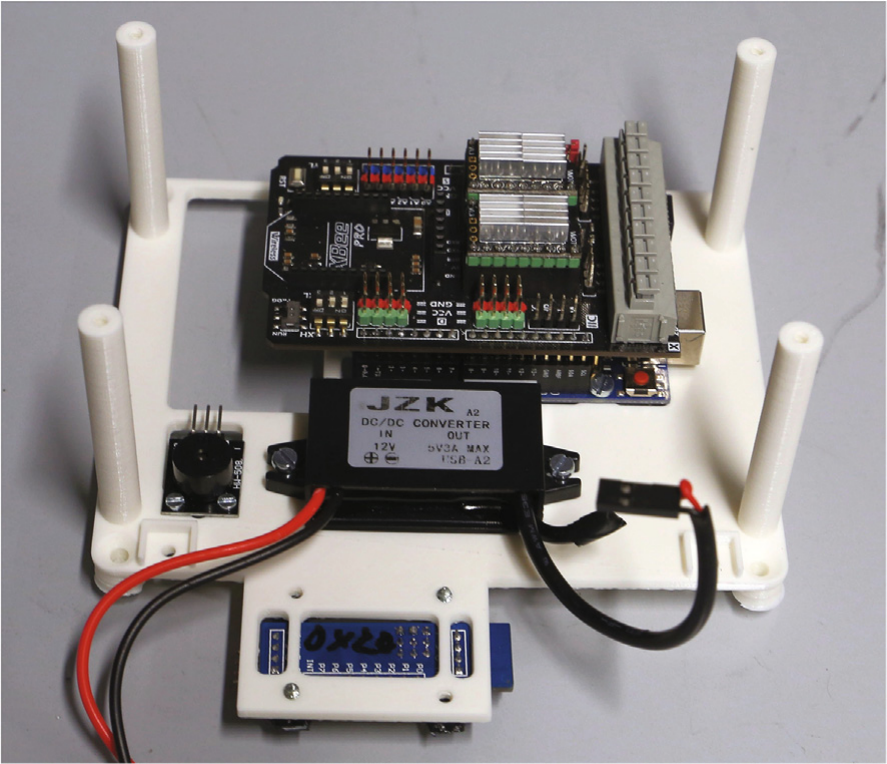
Electronics holder base with mounted electronic parts.

Place the Arduino Uno, the step-down transformer, and the active buzzer module on the “electronics holder base” and fasten them using M3 × 6 mm, M3 × 10 mm, and M2 × 6 mm bolts, respectively. Place the PCF8574T shift register module on the bottom side of the base and hold it in place using the “clips for shift register” and 3x10 mm wood screws. Add the DRV8825 motor shield on top of the Arduino. Place the base at the corresponding position on the aluminium baseplate, but do not fasten it yet (Fig. 26).

On top of the DRV8825 motor shield, all free pins are accessible as jumper jacks. According to the schematic shown in the Supplementary Information, connect the optical switches and servo motors to these jumper jacks. Next, connect the magnetic valve and the stepper motor to the motor shield. In the case of the magnetic valve, add blade receptacles to connect the wire to the valve, and add a freewheel diode as shown the Supplementary Information to protect the electronics from high-voltage spikes caused by the inductive load of the magnetic valve.

**Fig. 27:**
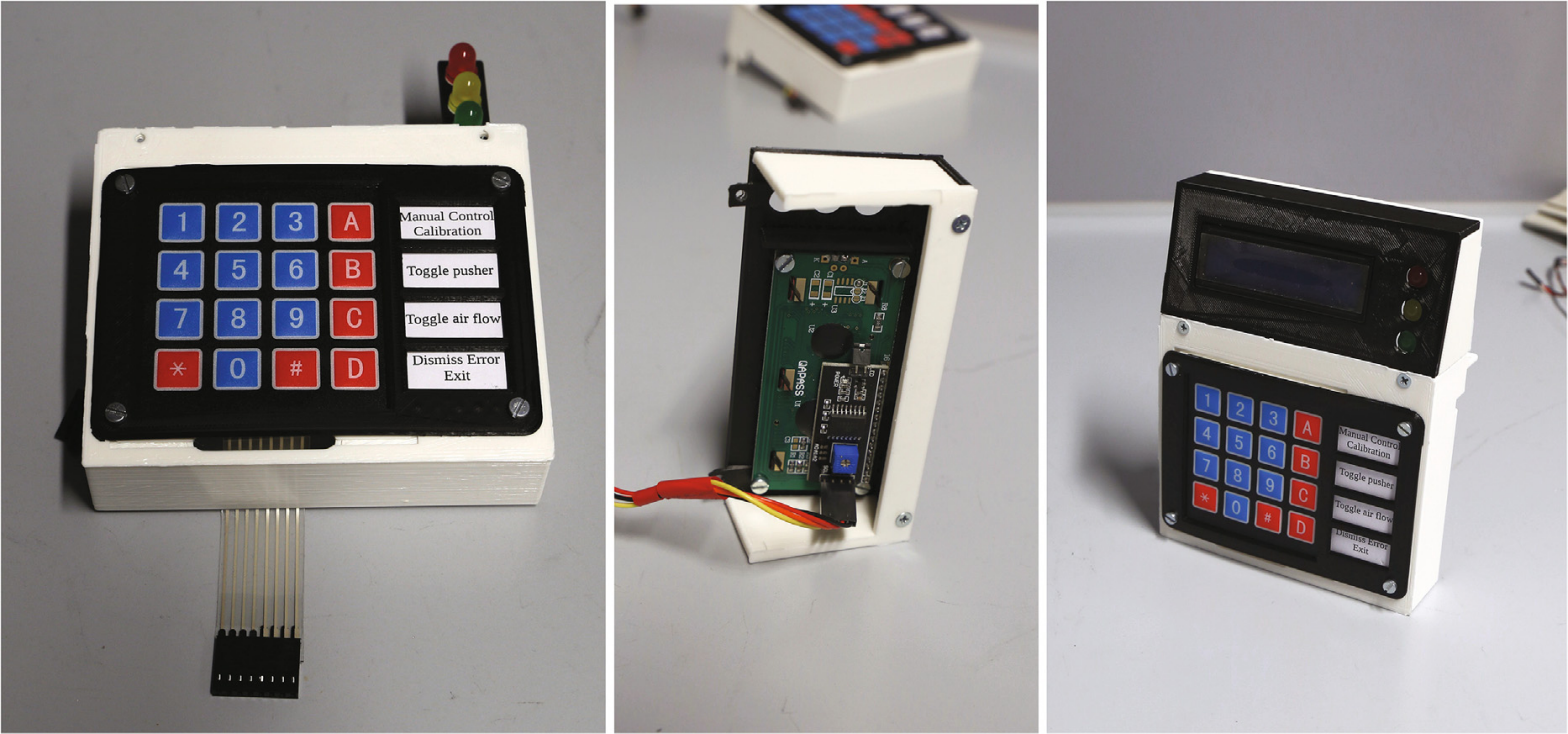
Keypad holder (left), display holder (center), and fully assembled manual control module (right).

Next, assemble the display and keypad. The keypad is placed on the part “keypad holder” together with a sheet of paper serving as a label (see Supplementary Information). The part “mounting bracket” is then placed on top and fastened using M3 × 6 mm cylinder-head screws. On the other side of the keypad holder, the LED traffic light module is added in the corresponding position and screwed down using 3 × 10 mm wood screws. Now, place the LCM1602 LCD display module on the part “display holder”, and fasten it using four M3 × 6 mm cylinder-head screws. Use a four-pin jumper connector to bend the pins of the I2C module of the LCD display to a vertical position, then place the display holder on the keypad holder and fasten it using 3 × 10 mm wood screws. Add the part “display holder side panel” and fasten it using M3 × 10 mm countersunk screws. Wire up the two I2C modules, the keypad, and the LED traffic light module according to the schematic in the Supplementary Information. Pay close attention to the correct wiring of the VCC, GND, SDA, and SCL pins, as the order of these pins will differ between the motor shield and both I2C modules. Optionally, to simplify maintenance, the wires may be grouped in appropriate 3- and 4-pin Dupont connectors. Place the keypad holder into the corresponding slots on the electronic holder base and fasten it using M3 × 10 mm countersunk screws. The assembly of the manual control module is illustrated in Fig. 27. Now, screw down the base onto the aluminium baseplate to keep it in place.

**Fig. 28:**
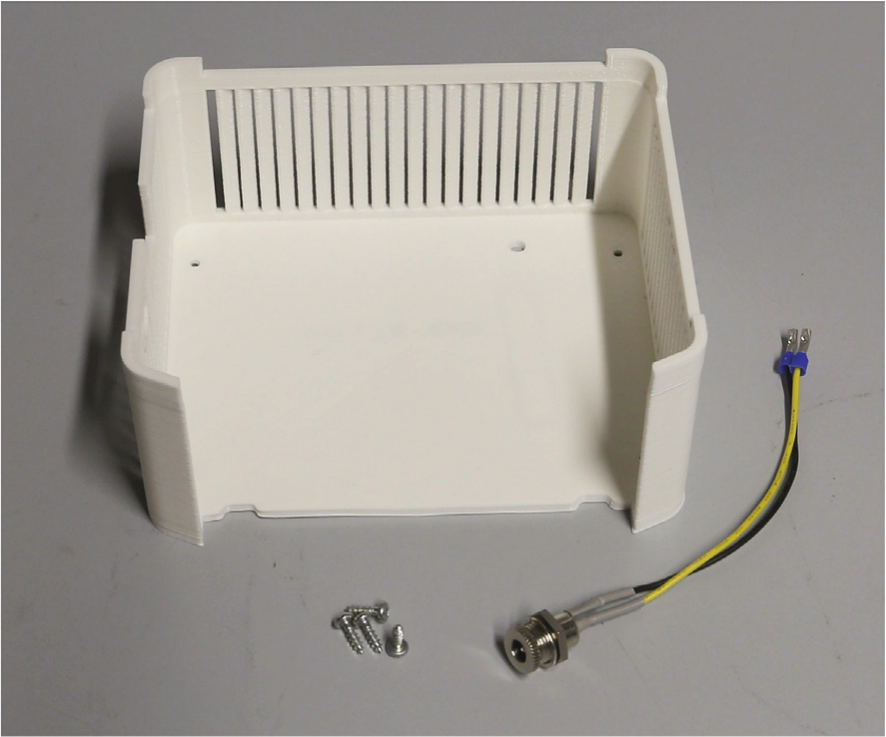
Parts required for the assembly of the electronics lid.

**Fig. 29:**
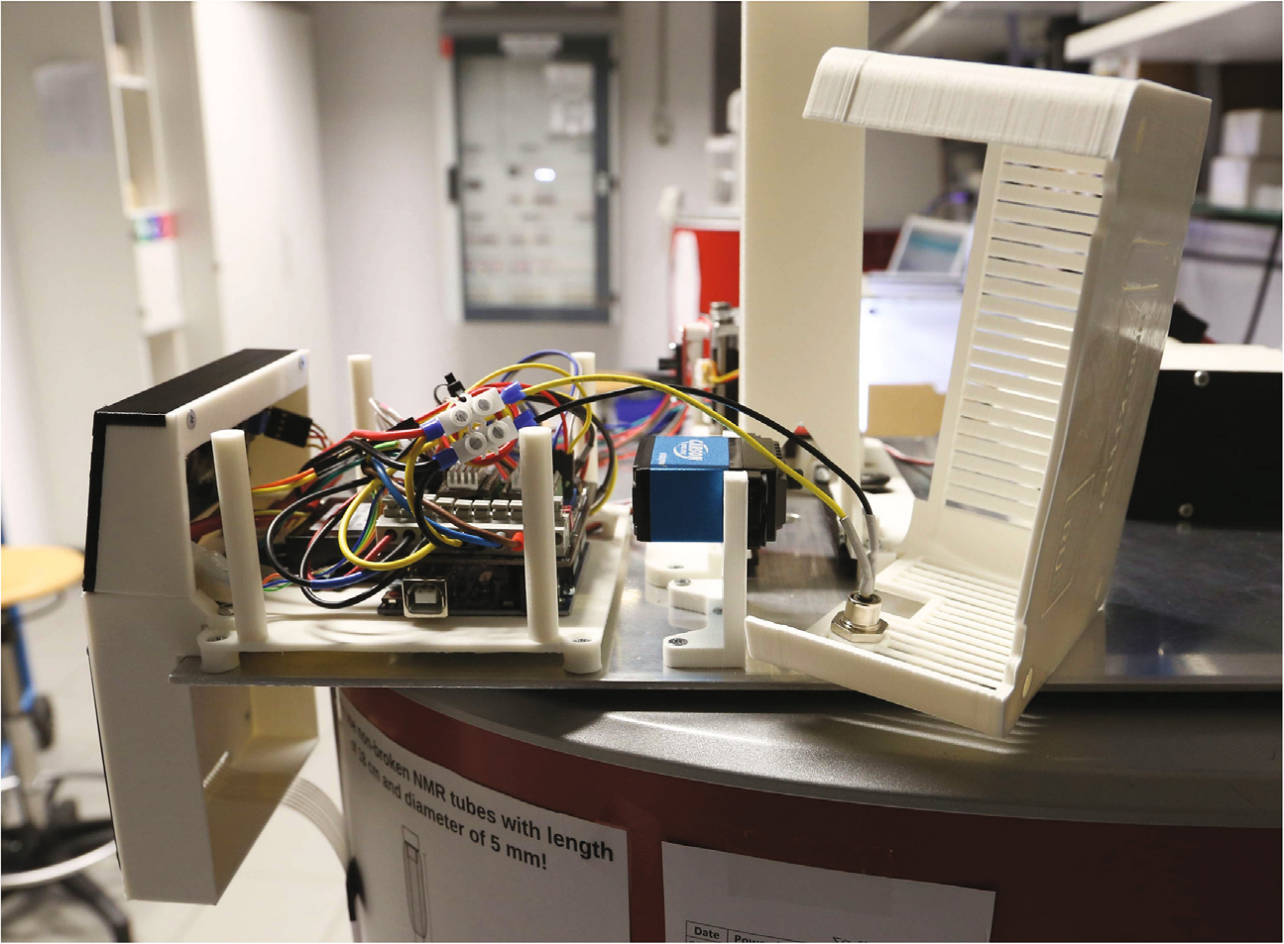
The fully assembled electronics of the autosampler.

The final part of the electronic assembly is the lid (Fig. 28). First, solder the wires to the DC power jack and isolate the exposed terminals using heat-shrink tubing. Screw the DC power jack into the round opening in the side of the lid. Connect the 12 V DC and ground terminals from the power jack with the corresponding contacts of the step-down transformer, as well as with the motor shield, according to the schematic in the Supplementary Information. Finally, connect the 5 V DC output of the step-down transformer directly to the + 5 V and GND terminals on the Arduino Uno. The fully assembled electronics of the autosampler are shown in Fig. 29.

**Fig. 30:**
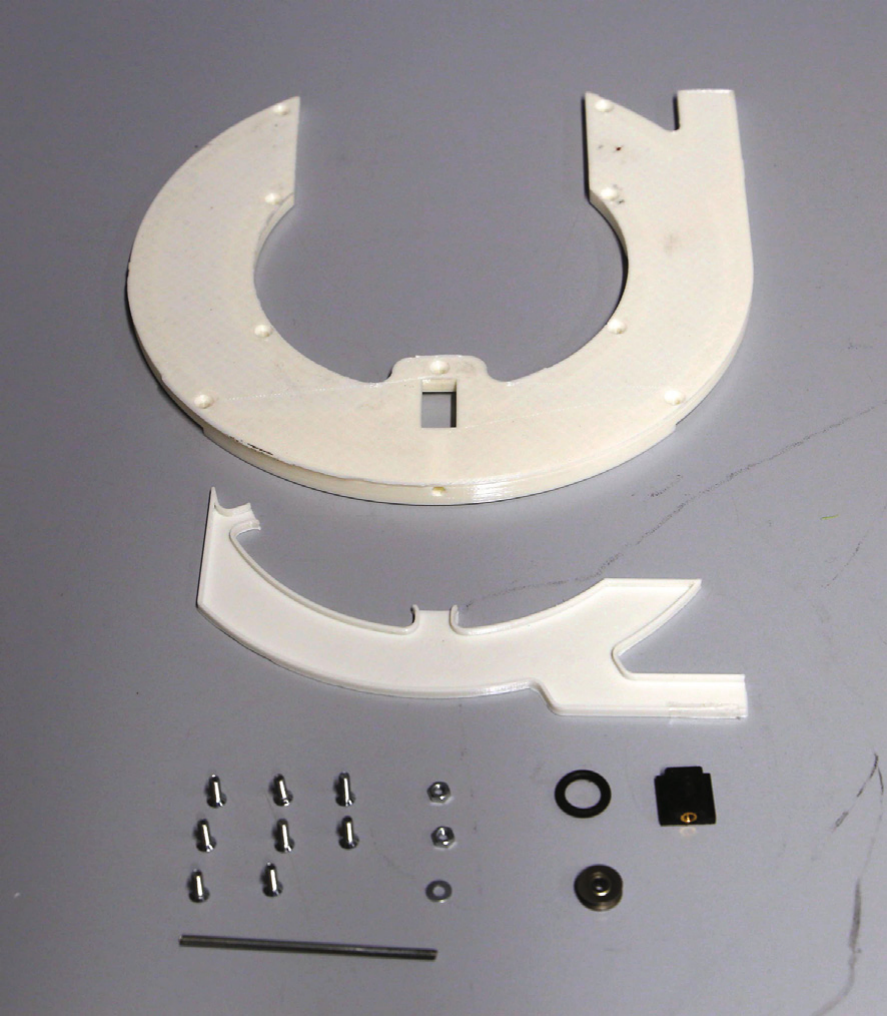
The parts required for cable management and the assembly of the sliding ring.

**Fig. 31:**
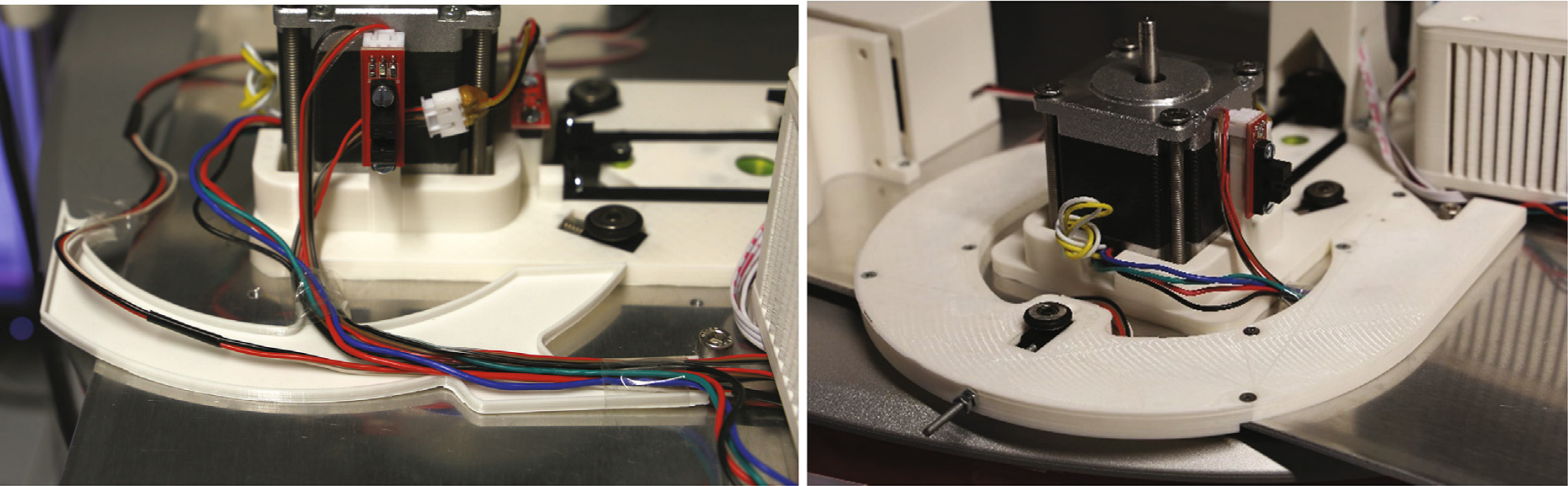
Cable management. It can be helpful to use tape to hold the wires in place.

The wiring of the autosampler runs underneath the “sliding ring”, on which the samples are moving (Fig. 30). Use the “cable duct” to guide the wires for the air servo, stepper motor, and the two optical switches next to the stepper motor (Fig. 31). Assemble the limiter block by adding an M3 threaded insert into the corresponding hole and assembling the parts as shown in 5.1.4, “*Main motor holder, tower, and servo holder*”, and add it to the sliding ring. Place the sliding ring carefully on top of the cable duct without pinching any wires, and screw it into the aluminium baseplate using eight M3x10 mm countersunk screws.

Finally, add the lid of the electronics holder on top and fasten it to the base using 3x10 mm wood screws.

#### 5.1.6 Slider

**Fig. 32:**
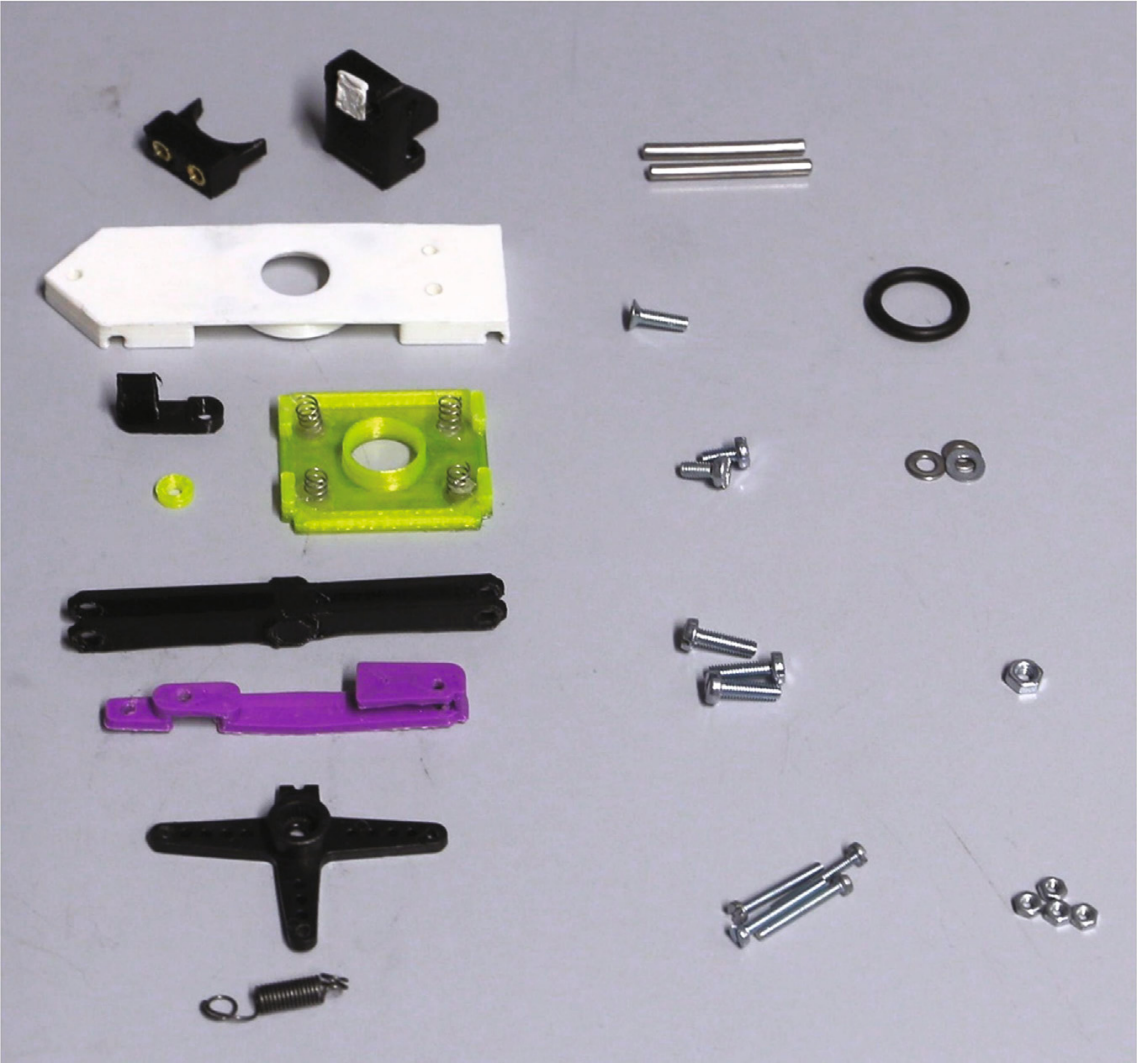
The parts required to assemble the slider.

**Fig. 33:**
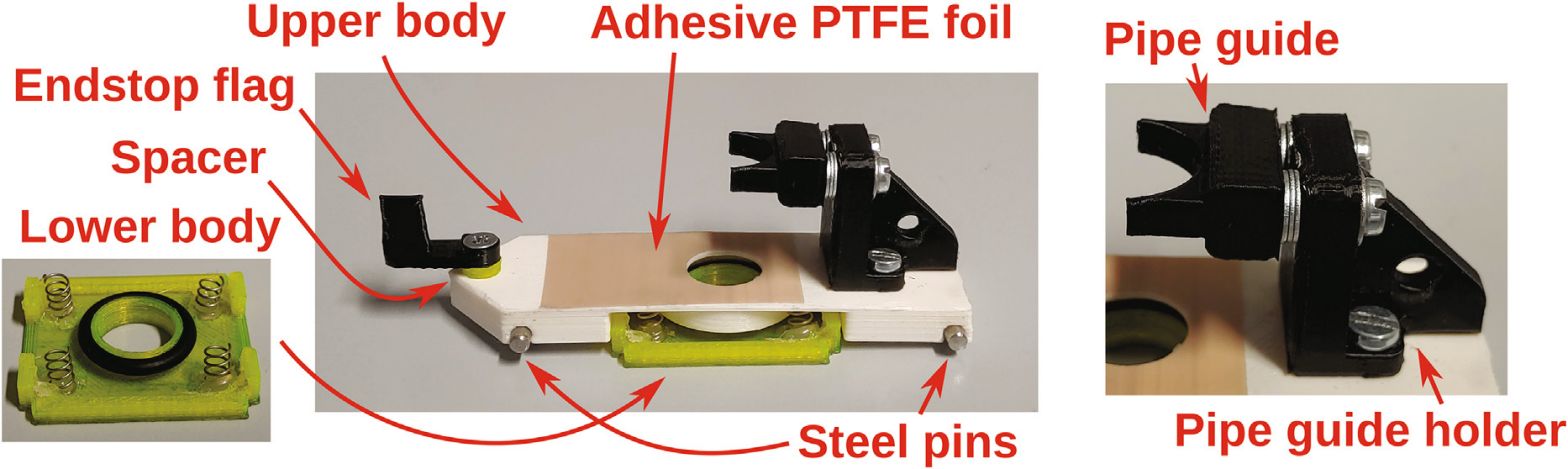
The fully assembled slider.

The slider (Fig. 33) is responsible for opening and closing access to the spectrometer cavity. It precisely aligns the rotor with the other parts of the assembly and holds it in place during the process of inserting or ejecting a sample. The parts required for the assembly of the slider are shown in Fig. 32, a list of parts is given in Table 6. To assemble the slider, start by cutting two compression springs in half. Use hot glue to attach the springs to the corresponding slots of the slider’s lower body. Add an O-ring (13 × 2.5 mm) on the corresponding groove in the lower body.

**Table 6:** List of parts required for the assembly of the slider.

Amount	Component	Type
1	Slider – Lower body	3D-printed part
1	Slider – Upper body	3D-printed part
1	Slider – Pipe guide holder	3D-printed part
1	Slider – Pipe guide	3D-printed part
1	Slider – Spacer	3D-printed part
1	Slider – Endstop Flag	3D-printed part
1	Pulling dampener	3D-printed part
1	Drive shaft	3D-printed part
2	Steel pins 30 × 3 mm	Off-the-shelf part
4	Compression springs, VD-052F	Off-the-shelf part
1	Tension spring, RZ-045E-02I	Off-the-shelf part
1	O-Ring, 13 × 2.5 mm	Off-the-shelf part
1	Cross-shaped drive arm (included with the Carson CS-13 servo motor)	Off-the-shelf part
2	M3 threaded heat inserts	Screws, Bolts, Nuts
1	M3 × 10 mm countersunk bolt (DIN 965)	Screws, Bolts, Nuts
2	M3 × 6 mm cylinder-head bolts (DIN 84)	Screws, Bolts, Nuts
3	M3 × 10 mm cylinder-head bolts (DIN 84)	Screws, Bolts, Nuts
4	M2 × 10 mm cylinder-head bolts (DIN 84)	Screws, Bolts, Nuts
3	M3 washers	Screws, Bolts, Nuts
1	M3 nut	Screws, Bolts, Nuts
4	M2 nuts	Screws, Bolts, Nuts

Next, take the upper body of the slider. It is important to print the upper body with 100% infill, as this part requires high structural rigidity. Insert the 3 × 30 mm steel pins into the two corresponding slots using a rubber mallet. At the tip of the upper body, mount the spacer and endstop flag using M3 × 10 mm countersunk bolts.

The pins will interact with the corresponding rails of the main motor holder to set the maximum height of the slider’s upper body, while the springs will hold the upper body at that maximum position. Due to the shape of the rails on the main motor holder, this results in raising and lowering the slider depending on its current position: The slider will be raised when a sample should be passed through, preventing a gap through which air could escape and therefore making sure that the sample can be lifted reliably with minimal airflow. It is automatically lowered upon retraction so that the rotor can turn freely without friction between rotor and slider.

On the other end of the upper body, mount the “pipe guide holder” using two M3x6 mm cylinder-head bolts. Add two M3 threaded inserts into the two corresponding holes of the part “pipe guide”. Place several M3 washers on the screws in between the pipe guide holder and the pipe guide, and bolt down the pipe guide using two M3 × 10 mm cylinder-head screws, using a washer on each screw. These two screws will later be used to fine-tune the alignment of the rotor with respect to the other parts of the autosampler.

Optionally, adding some adhesive PTFE foil on the top of the upper part of the slider may improve the seal between slider and rotor, improving air flow.

Now, the length of the springs mounted in the slider’s lower body must be adjusted. Use side cutters to shorten the springs until they have just enough force to fully lift the slider when inserted in the corresponding rails in the main motor holder.

**Fig. 34:**
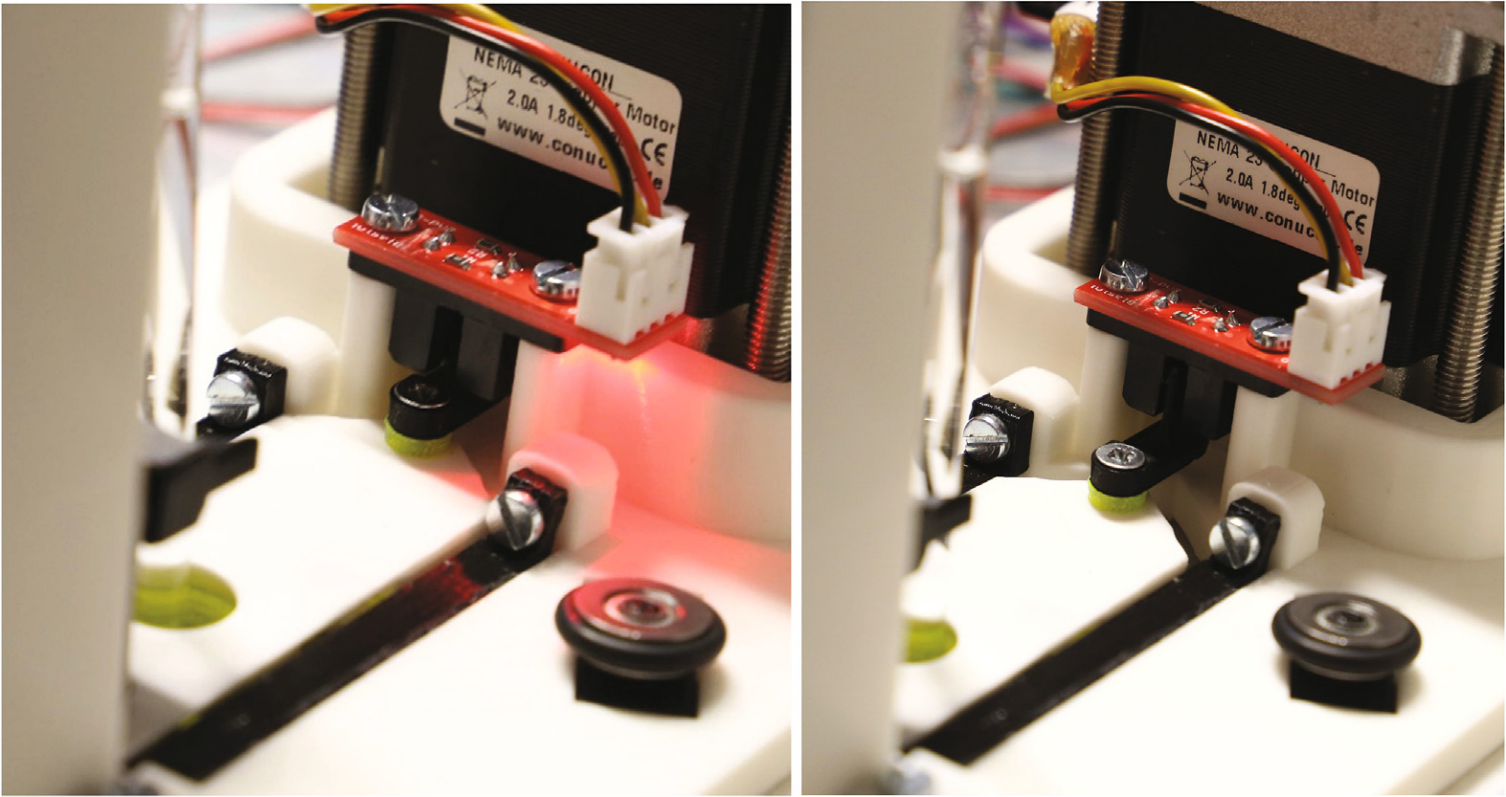
If an NMR tube is inserted, the red LED is only allowed to turn on if the slider is on its end stop position. When pulled back, the red LED must always stay off. (For interpretation of the references to colour in this Fig. legend, the reader is referred to the web version of this article.)

The slider’s “endstop flag” is an important safety mechanism, as it will detect whether the slider is able to correctly move in each direction. Thus, its correct operation must be tested. Turn on the Arduino (to supply power) and watch the red LED of the optical switch. When moving the slider forwards and backwards, the flag should pass through the optical switch. During this time, the red LED should turn off completely. Next, simulate that an NMR tube is not properly inserted or ejected by hovering an empty NMR tube over the spectrometer (see Fig. 34). Try to move the slider and ensure that the red LED never turns on. If the red LED ever turns on during this test, the size of the endstop flag must be adjusted.

**Fig. 35:**
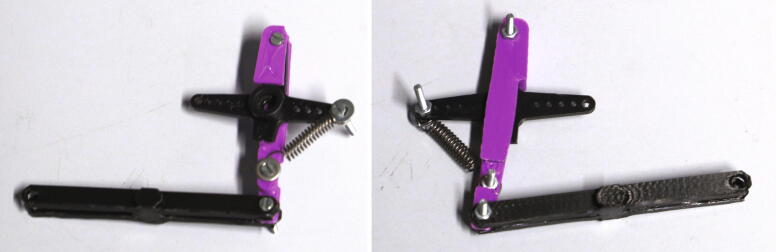
Pushing mechanism.

Finally, the connection between the slider and the servo motor must be assembled. Cut off one side of the servo’s cross-shaped drive arm as shown in Fig. 32 and drill out the three outermost holes to a diameter of 2 mm. Add the part “pulling dampener” and hold it in place using an M2 bolt and nut. Mount the tension spring according to Fig. 35 using two M2 bolts, washers, and nuts. Finally, attach the part “drive shaft” to the pulling dampener using a M2 bolt and nut. Connect the drive shaft to the slider’s pipe guide holder and fasten it using a M3 cylinder-head bolt and nut. This mechanism is another important safety feature, as it allows the servo motor to apply full force only during pushing, when it needs to correctly align the rotor. When pulling, however, the applied force is limited by the tension spring. Thus, if an NMR tube is stuck in the slider, this mechanism prevents breakage of glass.

Do not connect the mechanism to the servo motor yet: since the correct start- and endpoints are not yet known to the software, the servo motor might destroy the pulling dampener on first startup.

#### 5.1.7 Rotor

**Fig. 36:**
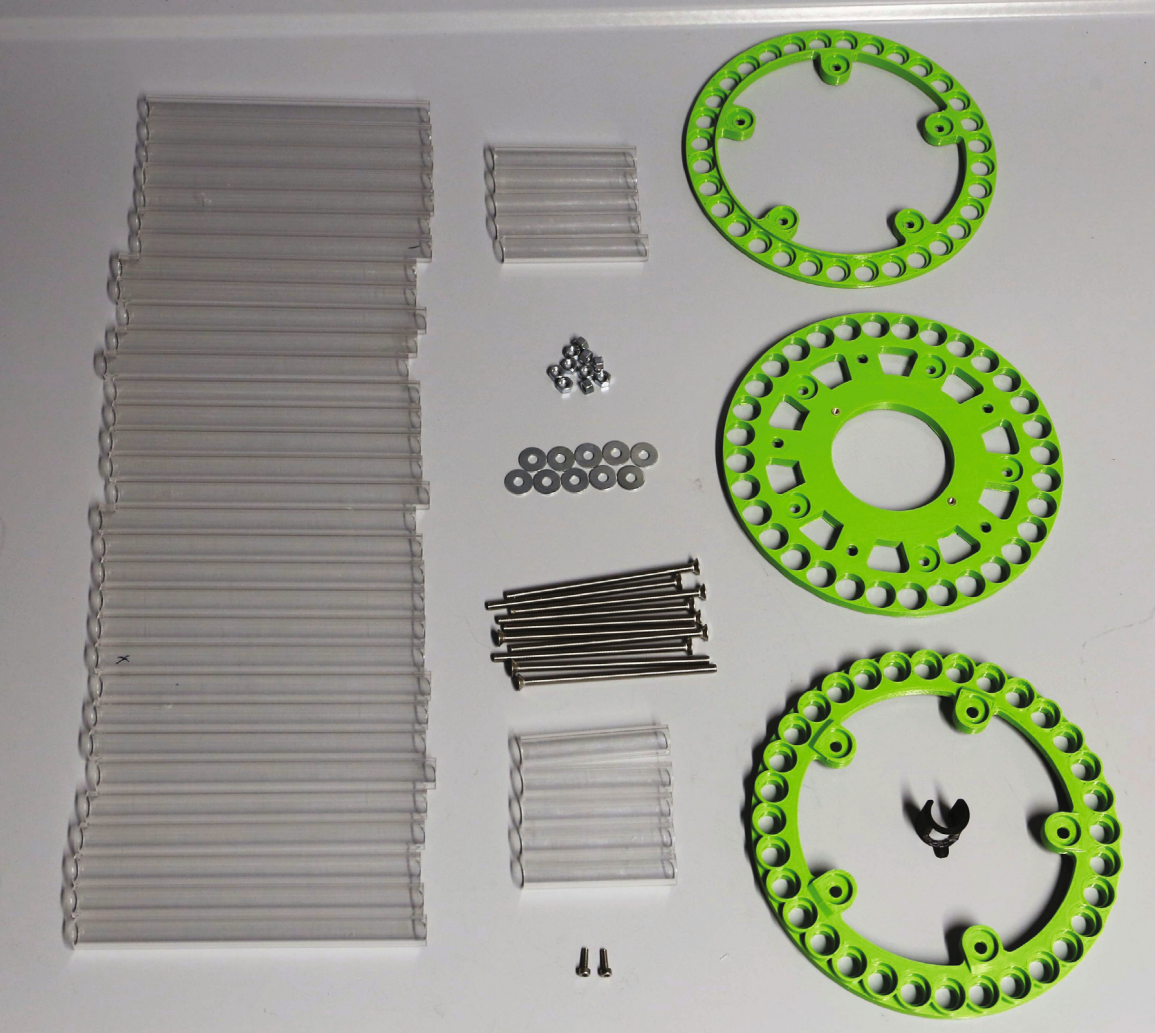
The parts required to assemble the rotor.

For the rotor, cut plastic tubes of the following lengths: 161 mm (32 pieces), 80.5 mm (5 pieces), and 72.5 mm (5 pieces).The parts required for the assembly of the rotor are shown in Fig. 36, a list of parts is given in Table 7.

**Table 7:** List of parts required for the assembly of the slider.

Amount	Component	Type
1	Rotor – Lower Ring	3D-printed part
1	Rotor – Middle Ring	3D-printed part
1	Rotor – Upper Ring	3D-printed part
1	Flag holder	3D-printed part
6 m	PMMA or Polycarbonate pipe, OD = 15 mm, ID = 11 mm	Off-the-shelf part
2	M3 threaded heat inserts	Screws, Bolts, Nuts
2	M3 × 10 mm cylinder-head bolts (DIN 912)	Screws, Bolts, Nuts
10	M5 × 100 mm countersunk bolts (DIN 965)	Screws, Bolts, Nuts
10	M5 nuts	Screws, Bolts, Nuts
12	M5 washers, d = 15 mm	Screws, Bolts, Nuts

It is recommended to print the rotor rings from a different colour than that used for the slider, as this will help to distinguish interfering parts during the final adjustment when looking from above (Section 5.3.2, “*Fine-tuning the air pressure, slider, and rotor placement*”).

**Fig. 37:**
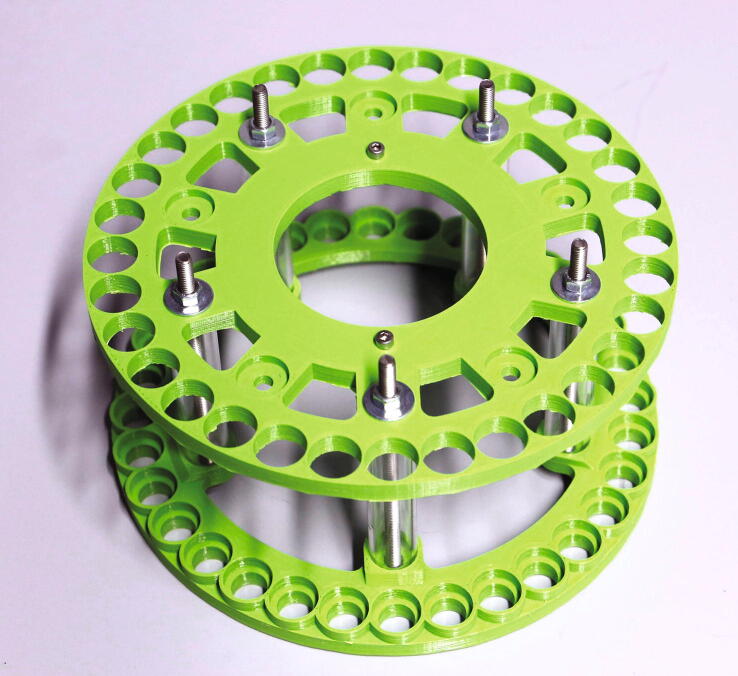
First step in the assembly of the rotor.

To assemble the rotor, start by adding the two M3 threaded inserts into the corresponding holes in the middle ring. Then, connect the lower and middle rings with the 72.5 mm plastic tubes and use the bolts, washers, and nuts to hold them in place (Fig. 37). Pay attention to the alignment of the rotor parts: there is only one correct position in which both the five-membered inner rings and the 32-membered outer rings overlap.

Make sure that the bottom surface of the lower ring of the rotor is as even as possible. If there are spots which are higher or lower compared to the rest of the ring, these bumps can be corrected by adding additional washers into one or two spokes, in between the plastic tube and the ring.

**Fig. 38:**
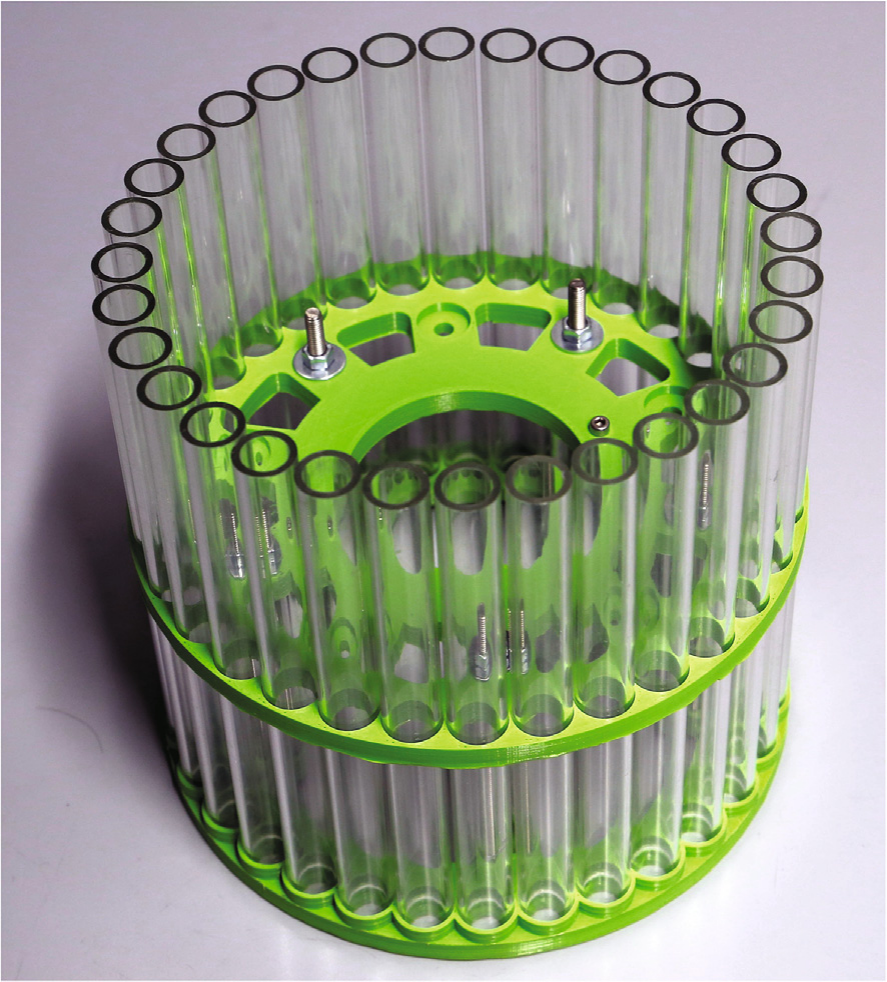
Second step in the assembly of the rotor.

Continue by adding the 161 mm plastic tubes into the corresponding holes (Fig. 38).

**Fig. 39:**
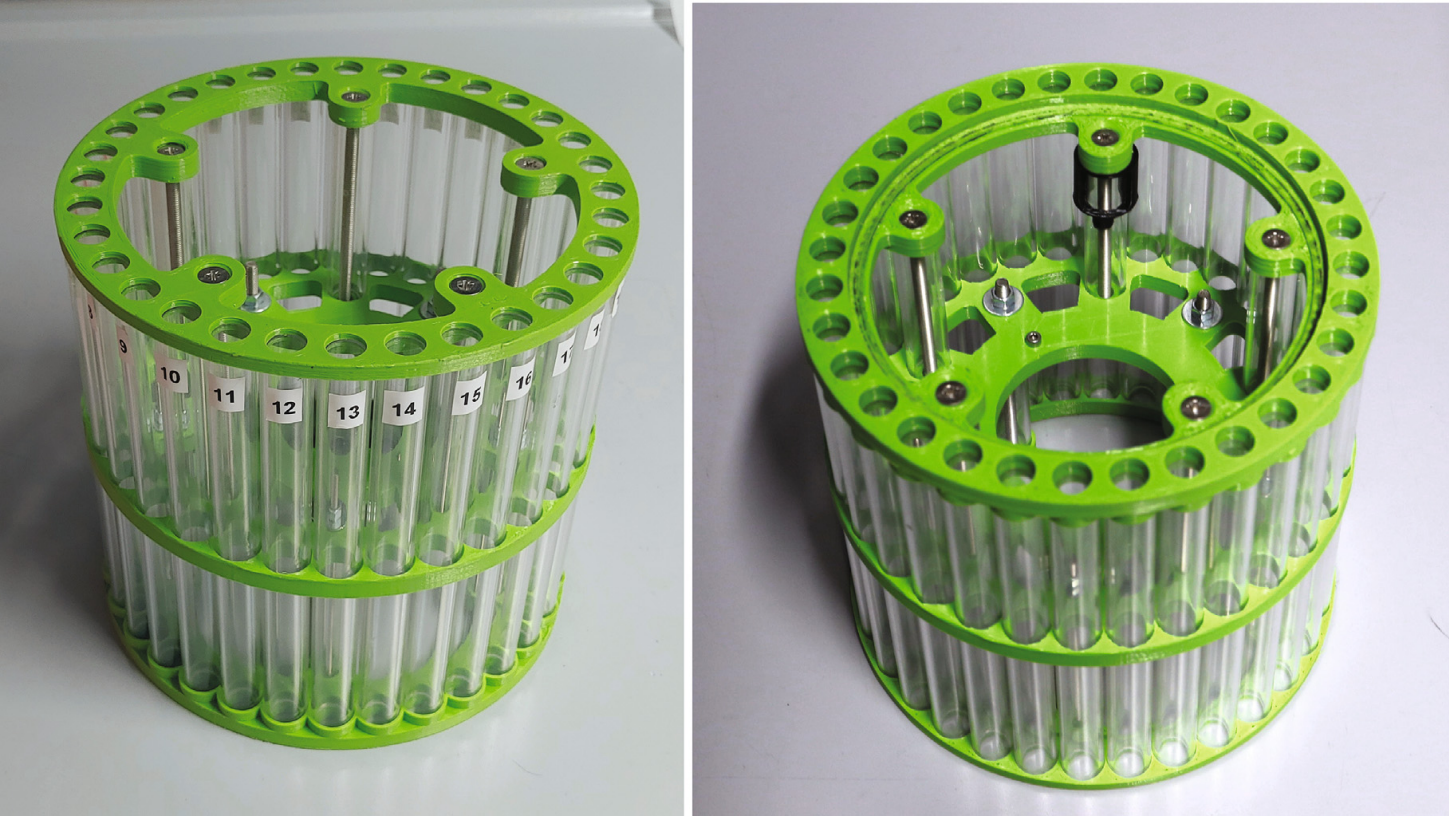
Fully assembled rotor, viewed from the top (left) and bottom (right). The number labels are attached upon first operation (Sections 5.1.7 and 5.3.1.1, "*Rotor*").

Next, add the 80.5 mm plastic tubes into the corresponding holes and add the upper ring on top of the assembly. Insert all tubes into the corresponding holes and use the remaining M5 bolts, washers, and nuts to hold the assembly together. In contrast to the bottom side of the rotor, the top side does not need to be level (See Fig. 39).

Finally, screw the M3 × 10 mm cylinder-head bolts into the bottom side of the middle ring. Then, clip the part “flag holder” to one of the bottom spokes. This part will serve to define the zero-point of the rotor. If the part is too loose, use hot glue to glue it in place.

#### 5.1.8 Connection of the rotor to the stepper motor

**Fig. 40:**
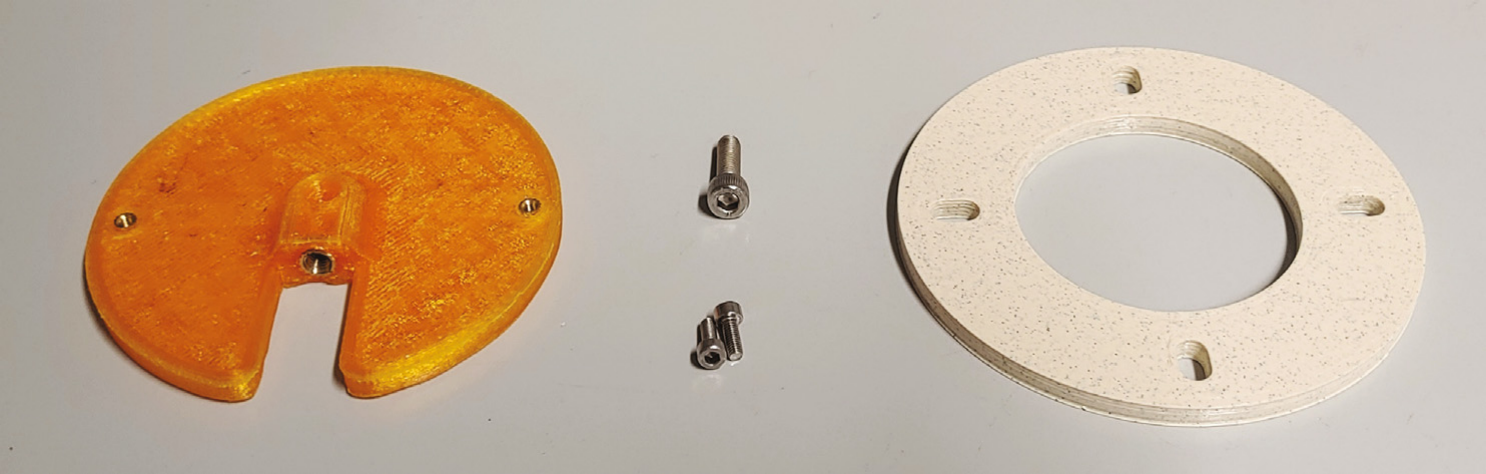
Parts required to connect the rotor to the stepper motor.

One of the main problems with using a 3D-printed rotor is the inherent imprecision of large 3D-printed parts. Due to thermal contraction upon cooling of the print, there will always be some warping in any object printed with an FDM-type 3D printer. In the case of the rotor, this means that the rings will be somewhat elliptical. To counteract this problem, the part shown in this section allows the rotor to horizontally move several millimeters in each direction. When inserting or ejecting a sample, the spring blocks and the slider will then precisely align the tube of the rotor with the spectrometer. The parts required for the connection of the rotor to the stepper motor are shown in Fig. 40, a list of parts is given in Table 8.

**Table 8:** List of parts required for the connection of the rotor to the stepper motor.

Amount	Component	Type
1	Motor flange	3D-printed part
1	Clutch ring	3D-printed part
2	M3 threaded heat inserts	Screws, Bolts, Nuts
1	M5 threaded heat insert	Screws, Bolts, Nuts
2	M3 × 10 mm cylinder-head bolts (DIN 912)	Screws, Bolts, Nuts
10	M5 × 20 mm cylinder-head bolts (DIN 912)	Screws, Bolts, Nuts

**Fig. 41:**
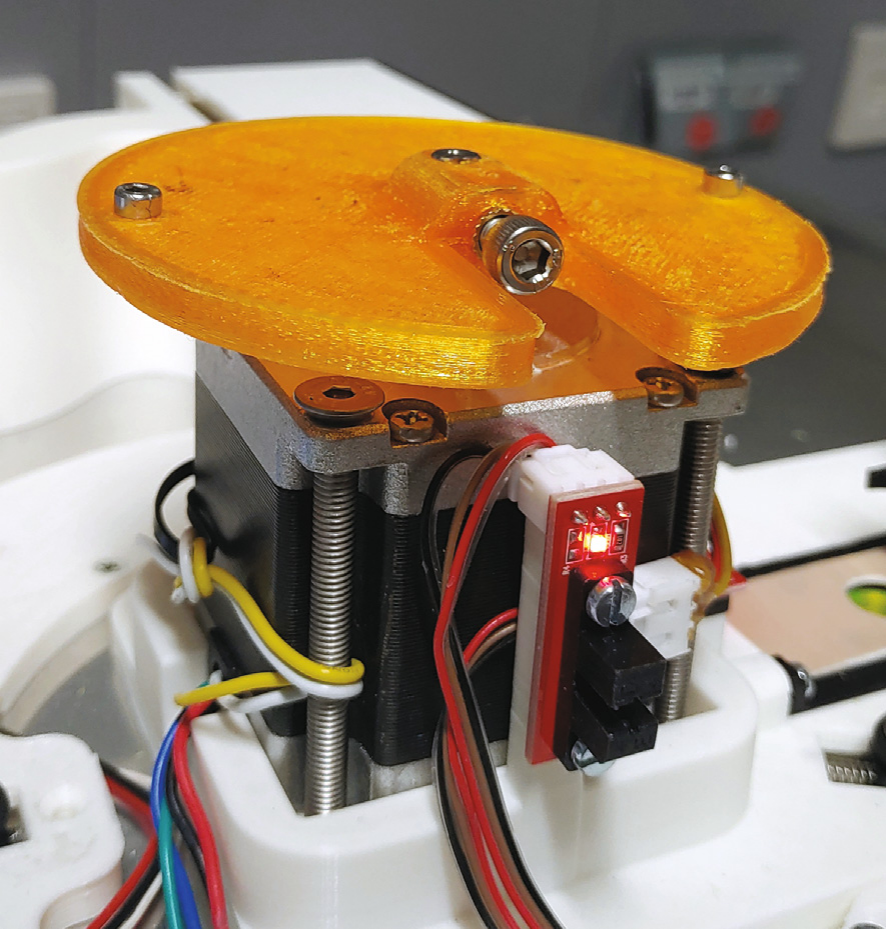
The motor flange mounted on the motor shaft.

**Fig. 42:**
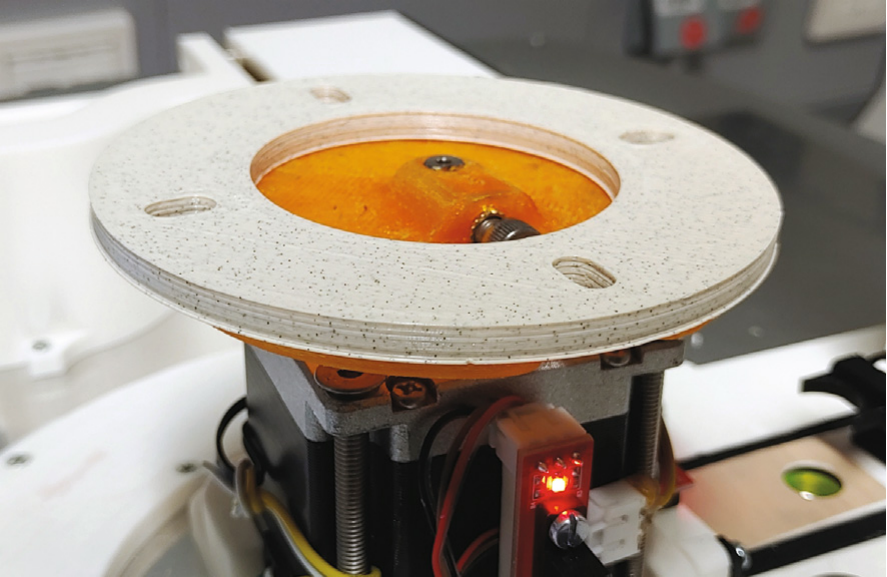
Clutch ring added on top of the motor flange.

To assemble the system, start by adding the M3 and M5 threaded inserts into the corresponding holes in the “motor flange”. This part should be printed from PETG instead of PLA, as PLA has a very low glass transition temperature, which might result in loosening of the M5 threaded insert due to the heat of the motor during operation. Screw the M3x10 mm cylinder-head bolts into the upper side of the motor flange. Place the motor flange onto the stepper motor shaft and use the M5 bolt to hold it in place (Fig. 41). Then, place the “clutch ring” on top of the motor flange (Fig. 42).

The rotor can then be mounted on top of the clutch ring by placing it such that the cylinder-head bolts of the rotor are inserted into the free slots of the clutch ring.

**Fig. 43:**
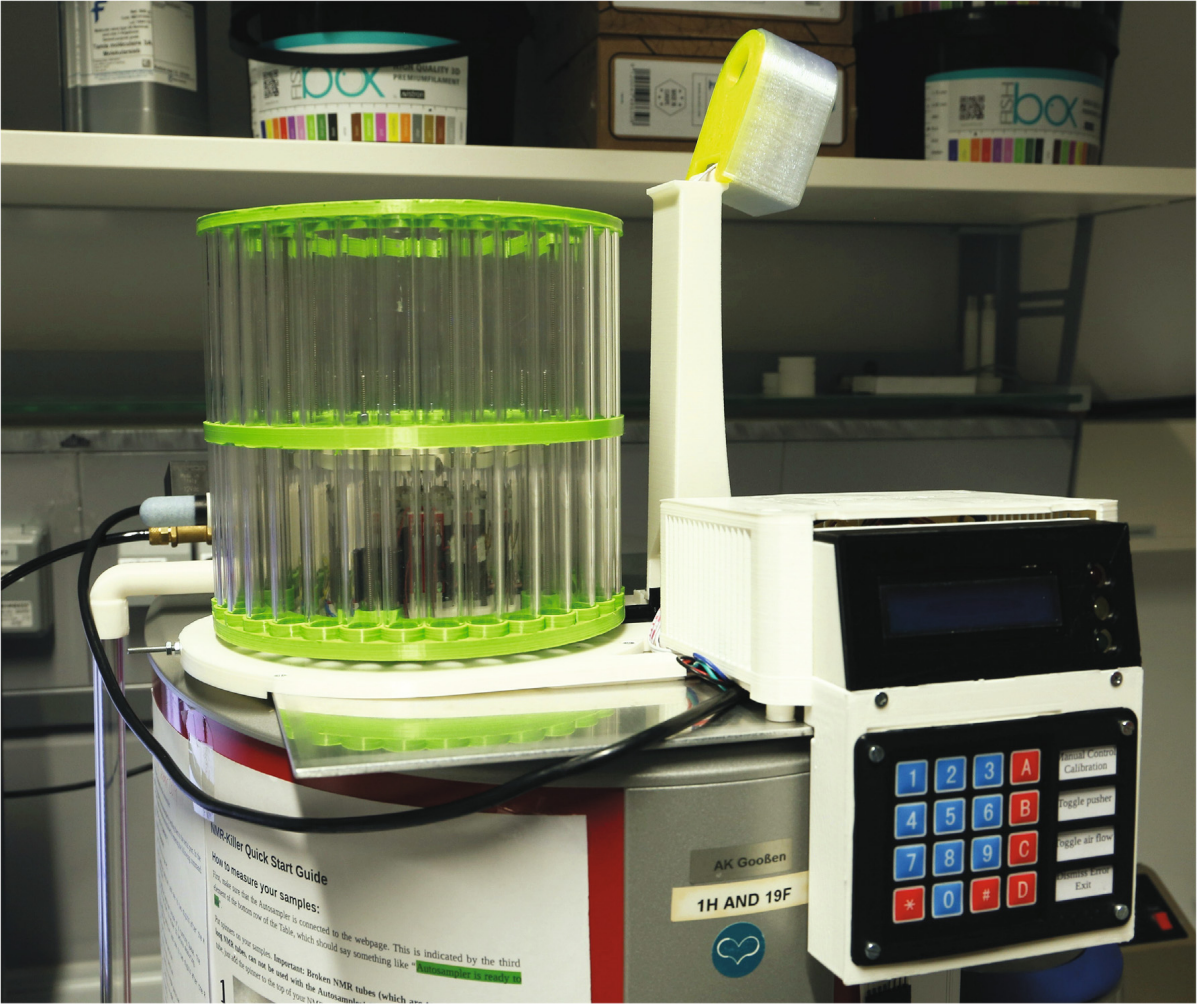
Fully assembled autosampler.

Lastly, set the height of the rotor so that the gap between the sliding ring and the rotor is around 1 mm. To do this, dismount the rotor and clutch ring, loosen the M5 bolt keeping the motor flange in place, and adjust the height of the motor flange (See Fig. 43).

#### 5.1.9 Spinners

**Fig. 44:**
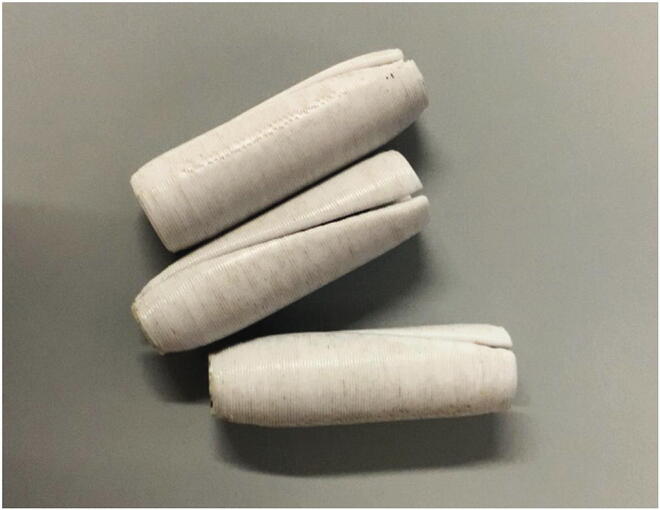
Three spinners.

The “spinner” (Fig. 44) is added onto an NMR tube which is to be measured and ensures that it stays in the correct position (Section 6.1, “*Sample preparation and measurement*”). While it does not actually spin during the measurement, we still decided to call it “spinner” due to its similarity to the spinners found in high-field NMR spectrometers. The spinners are 3D printed from PLA, but because 3D printing does not reach the required precision, they must be post-processed. We strongly recommend against using spinners which haven’t been post-processed, since they may have irregular shapes which could lead to local stress on glass NMR tubes, potentially breaking them.

For a video tutorial on the post-processing of 3D-printed spinners, see the Supplementary Information.

### 5.2 Setup of software

The current version of the software can be found on the project’s GitHub repositories. The location of GitHub repositories can be found in Section 3 (“*Design files*”). Follow the instructions given in the repositories to setup the software.

### 5.3 First operation

#### Warning

Before first operation, the device is fully uncalibrated. Do not connect the slider (Section 5.1.6, “*Slider*”) to the servo, otherwise the servo may jump to an unpredictable position and break parts. Make sure that the compressed air regulator (Section 5.1.2, “*Insertion and ejection using compressed air*”) is set to the lowest possible pressure.

#### 5.3.1 First calibration

##### 5.3.1.1 Rotor

On first operation, the holders are not yet numbered. Run a homing by pressing the button A three times on the keypad, and then pressing *. The homing will move the rotor until the optical switch is triggered by the “flag holder” mounted on one of the rotor’s spokes, and will define the “zero-point” of the rotor as the middle of the flag. Assign holder number 1 to the tube which most closely aligns with the slider and spectrometer after that homing. Label the holders, e.g. with stickers, by starting with holder 1 and continue to the right until holder 30. Holder 31 is reserved for the shimming sample and should be labelled accordingly, e.g. with the letter “S”. Finally, holder 32 should always be empty, as the autosampler will use this holder to check whether a sample was already inserted before starting a measurement. After numbering the holders, proceed with the calibration as described in Section 5.4.1, “*Calibration of the rotor*”.

##### 5.3.1.2 Slider

On first operation, the slider’s position is completely unpredictable. Therefore, it must be disconnected from the servo motor. Go to the slider calibration mode (see Section 5.4.2, “*Calibration of the slider*”) and set the current position to 90. Then, connect the slider by pushing the servo’s drive arm onto the servo motor and proceed as described in Section 5.4.2, “*Calibration of the slider*”.

##### 5.3.1.3 Compressed air

On first operation, the air pressure required to eject a sample is not yet known. Make sure that the pressure regulator is set to the lowest possible setting, then slowly increase it to around 2 bar – this should be sufficient to allow calibration of the air servo. Continue as described in Section 5.4.3, “*Calibration of the airflow regulator*”, and then fine-tune the air pressure as described in Section 5.3.2, “*Fine-tuning the air pressure, slider, and rotor placement*”.

#### 5.3.2 Fine-tuning the air pressure, slider, and rotor placement

The autosampler can only operate if 1) the tubes of the rotor are properly aligned with the slider, aluminium baseplate, and spectrometer, and 2) the compressed air flow is strong enough to lift samples to the top of the tower.

To align the rotor’s tubes, turn the rotor so that a holder aligns with the spectrometer hole, and use the “B” button to toggle the slider. Pivot the upper part of the tower and look straight down into the spectrometer through the rotor’s tube. This way, the alignment of the tubes can be observed: If the rotor’s tubes are not properly aligned, a crescent-shaped part of the slider will be visible from above. Use the M3 screws connecting the part “pipe guide” to the part “pipe guide holder” to adjust the position of the rotor’s tube relative to the slider, until the crescent-shaped part is as small as possible. It may be necessary to disconnect the pipe guide and adjust the amount of washers between the pipe guide and pipe guide holder. To test the alignment, add an empty NMR tube with cap and spinner to a holder, rotate the rotor until it aligns with the spectrometer’s hole, and let it drop down by toggling the slider using the B button on the keypad. A tutorial video for the rotor alignment is found in the Supplementary Information (Rotor Alignment Video).

Next, adjust the compressed air. The initial air pressure, which is set on the regulator assembled in Section 5.1.2, “*Insertion and ejection using compressed air*”, is the main way to adjust the air flow through the spectrometer. On first operation, it should be set to minimum. Press the C button to toggle compressed air and slowly increase the air pressure on the regulator until the sample is lifted to the top of the tower. A pressure of 4–5 bar should be more than sufficient; if it is not, do not try to further increase the pressure, but check for leaks. Commonly, leaks can occur in the connection between the flow regulator and spectrometer, and can be sealed using Teflon tape. A tutorial video for adjusting the compressed air flow is found in the Supplementary Information (Pressure Video).

If the rotor is mounted in a position which is too high, air may escape between the slider and rotor, preventing the sample from being lifted. In this case, dismount the rotor and slightly lower the motor flange.

Note that correctly aligning these parameters for *one* holder might not necessarily result in the correct settings for *all* holders. In fact, expect to repeat this step several times, until a reliable setting for all holders is found.

### 5.4 Calibration

**Fig. 45:**
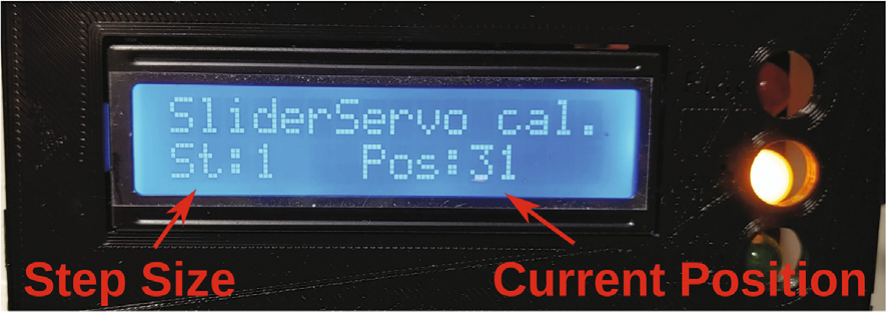
Calibration mode.

All calibrations can be accessed by repeatedly pressing the A button on the keypad. In calibration mode, the green and yellow LEDs will blink and the LCD display will show the values for “step size” and “position” (Fig. 45). The step size can be adjusted using the numbered keys. The * and # keys will then move the corresponding part forward or backward by adding or subtracting the step size from the position. In all calibrations, the goal is to find and save certain positions which must be known to the autosampler during operation. These parameters are constant during normal operation, but can change if certain parts are exchanged. The calibration processes are described in detail in the chapters below, as well as in the tutorial videos (Supplementary Information).

#### 5.4.1 Calibration of the rotor

The zero-point of the rotor is determined by the “flag holder” attached to one of its spokes, which will pass through the corresponding optical endpoint switch at a certain point. The rotor calibration serves to put this zero-point in relation to the individual holder numbers.

Before starting the rotor calibration, run a homing by pressing 3x A, and then * on the keypad. Then, press A until you reach the rotor calibration mode and use the * and # buttons to move the rotor until the slider and spectrometer align with the tube “1″. The step size can be adjusted by using the number buttons. Try using as few steps as possible. Press B to save the value “POS1”.

Recalibration of the rotor is required if the “flag holder” is exchanged or moved to a different rotor spoke.

#### 5.4.2 Calibration of the slider

The servo motor attached to the slider needs to know the start and end positions of the pushing process.

To calibrate the slider, start by finding the most retracted position possible. Use the number buttons to change the step size, and use the * and # buttons to move the slider around. Find a position where the hole of the slider is completely removed from the inside of the rotor’s tubes, but the slider’s endstop flag does not get hit by any of the spokes of the rotor when the rotor is being turned. Press C to save the value “SLIDER_PULL”.

Next, use the * and # buttons to find the position in which the slider fully extends towards the stepper motor. Save the value “SLIDER_PUSH” by pressing B.

Make sure that the servo motor applies enough force to self-right the rotor: Leave calibration mode by pressing D and turn the rotor to a deliberately imperfect position, then press B to toggle the slider. Unless your chosen position is right in the middle between two holders, the slider should be able to apply enough force to fully correct it and align the holder’s tube with the rest of the machine. If this is not the case, you need to further adjust the “SLIDER_PUSH” value.

Recalibration of the slider is required if the slider has been disconnected and reconnected from the servo motor.

#### 5.4.3 Calibration of the airflow regulator

Similar to the slider servo, the air servo needs to know the positions in which it is connecting the air supply to either the vent hole or the spectrometer.

First, exit calibration mode by pressing D. Use the B and C buttons to open the slider and start air flow. Go to air calibration mode and use the * and # buttons to find the position in which all of the air is vented without entering the spectrometer. Save the value “AIR_VENT” by pressing C.

Then, use the * and # buttons to find the position in which all of the air is passed through the spectrometer. Save the value “AIR_PUSH” by pressing B.

Recalibration of the airflow regulator may be required if the “inner part” of the airflow regulator is disconnected and reconnected from the servo motor.

### 5.5 Testing

On the keypad, press A repeatedly until you are offered the option to run a full test, then press *. The full test requires to completely fill all holders (including holder 32) with samples equipped with spinners. It will then try to insert and eject the samples in each position twice. If the test fails, refer to Sections 5.3.2, “*Fine-tuning the air pressure, slider, and rotor placement*”, and 6.4.1, “*Sample stuck inside of the spectrometer*”, to fix the issues.

## 6 Operation instructions

### 6.1 Sample preparation and measurement

For measurement of pure compounds, dissolve them in an appropriate solvent. Reaction mixtures can be measured directly, as the *Spinsolve* spectrometer does not rely on deuterium locking. Make sure that all analytes (substrates, products, internal standard) are fully dissolved. If in doubt, an appropriate solvent (e.g. ethyl acetate) can be added. If the mixture is not homogeneous, solid impurities can be filtered out using a Pasteur pipette filled with a cotton plug and celite.

Add the solution to an NMR tube. Only NMR tubes with a length of 18 cm and a diameter of 5 mm can be used with the autosampler. Longer NMR tubes might break when moved, as they can get caught on the tower. Upon attempting to insert the sample, the broken glass and solution can cause damage to both the autosampler and the spectrometer. On the other hand, shorter tubes may not fully reach into the spectrometer, resulting in poor quality of spectra, and they may be difficult to remove from the autosampler. NMR tubes for measurements under inert atmosphere, e.g. screw-cap or Young tubes, cannot be used with the RotoMate autosampler, unless their total length is 18 cm and the design of the spinner is adapted for this type of NMR tube.

**Fig. 46:**
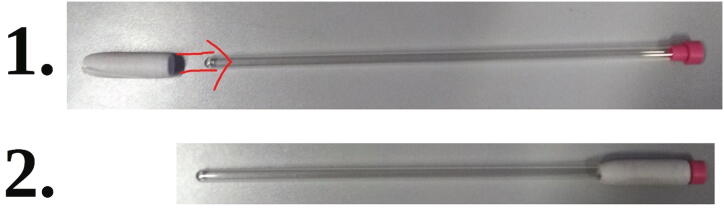
Correct placement of a spinner.

Add a spinner to the NMR tube. Make sure to use a spinner which fits the given NMR tube well, i.e. that the spinner can not easily move on its own, but can still be smoothly moved by hand. Push the spinner onto the NMR tube, until it reaches the cap (Fig. 46).

Then, place the sample into one of the available slots of the autosampler. Do not use holders 31 (marked “S”, reserved for the shimming sample) and 32 (should always be empty).

Make sure that the autosampler is running correctly. In the table of samples, this is indicated by the text “Autosampler is ready to use” or “Autosampler reports measurement is running”.

Enter the desired measurement parameters in the autosampler table. If the spectrometer has not been used for more than 24 h, it is recommended to run a shimming before your actual sample. To ensure a high signal-to-noise ratio, the number of scans must be sufficiently high. Typically, for a sample with a concentration of 0.5 M, 16 scans are sufficient, whereas for concentrations of 0.05 M, at least 128 scans are recommended. For quantitative measurements, it is important to set the repetition time to a sufficiently high value. For optimal accuracy, the repetition time should be set to five times the longest T_1_ relaxation time in the sample.^4^ Optionally, a start time can be specified for each measurement, allowing to run kinetic experiments. It should be noted, however, that samples without a specified start time will have priority over timed samples, so that it is advisable not to run other samples while at the same time conducting a series of timed experiments.

It is possible to add several experiments for the same holder, allowing to measure e.g. a proton and fluorine spectrum of the same sample without the need to eject and re-insert the sample.

Next, start the queue. The autosampler will first run a homing. As a safety mechanism, it will then move to Holder 32 and eject a potentially leftover sample from the spectrometer. This is done to prevent lowering a sample on top of another sample that may be inside the spectrometer. Finally, the rotor will move to the desired holder position, and the sample will be inserted.

### 6.2 Manual control

The autosampler can be manually controlled from the matrix keypad, as described in Table 9.

**Table 9:** Function of the keys on the matrix keypad.

Key	Description
A	Scroll through the basic maintenance functions, e.g.: Go to holder, insert/eject sample, perform homing, calibration, testing.
B	Toggle the slider.
C	Toggle the compressed air flow.
D	Dismiss any messages and errors. Close any menus which are open. If an error occurred and was fixed, this key is used to unlock the autosampler.
*	OK
#	Cancel

Additionally, the autosampler can be controlled using the buttons in the Python GUI. More advanced commands can be sent directly to the autosampler by entering the command into the text field, and then clicking the “Yell!” button. A list of commands is shown in Table 10. The commands can be chained, e.g. “hm20z” will run a homing, move the rotor to position 20, and then make a beeping noise.

**Table 10:** Overview of the “yell” commands which can be sent from the PC to manually control the autosampler.

Command	Description
E	Send error to autosampler. This command will cause the autosampler to cease operation and display an error.
M	Measure a sample. An integer number from 1 to 32 must be added to the command to specify the holder number. Before measuring the sample, the autosampler will go to holder 32 and try to eject a sample, to ensure that there is no NMR tube already in the spectrometer.
N	Measure a sample. An integer number from 1 to 32 must be added to the command to specify the holder number.
R	Return a sample to a specified holder. An integer from 1 to 32 must be added to the command to specify the holder number.
a	Sets the slider to pushing position.
b	Sets the slider to pulling position. Turns off the compressed air, if it is running.
c	Slowly turns on the compressed air flow.
d	Slowly turns off the compressed air flow.
e	Ejects a sample to the current position.
h	Performs a homing.
i	Inserts a sample from the current position.
l	Locks the stepper motor.
m	Move the rotor to a specified holder. An integer from 1 to 32 must be added to the command to specify the holder number. Does not home the rotor, i.e. if the rotor has been moved by hand since the last homing, the results will not be accurate.
r	Resets errors, exits calibration modes, exits measurement mode.
t	Run a full test of the autosampler (Section 5.5, “*Testing*”)
u	Unlocks the stepper motor.
z	Makes a beeping sound using the buzzer.

### 6.3 Automatic processing

Optionally, the autosampler software can use the *ACD NMR Processor* to automatically process ^1^H- and ^19^F NMR spectra, generate JDX and PDF files, and calculate conversions and yields. If not yet done, specify the location of your installation of ACD (e.g. C:/ACDFREE12/) by clicking on the link “conFig. autosampler” and entering it under “ACD folder”.

The autosampler software is able to automatically send commands to the *ACD NMR Processor* via its built-in macro function. If an internal standard is specified, the autosampler software can then read the integrals from the JDX file generated by the software. This can for example be used to automatically determine conversions and yields in an *in-situ* analysis of a reaction mixture, or to determine the purity of a given sample. As an example, the determination of the EtOH content of EtOH/H_2_O mixtures will be used in the following section.

**Fig. 47:**
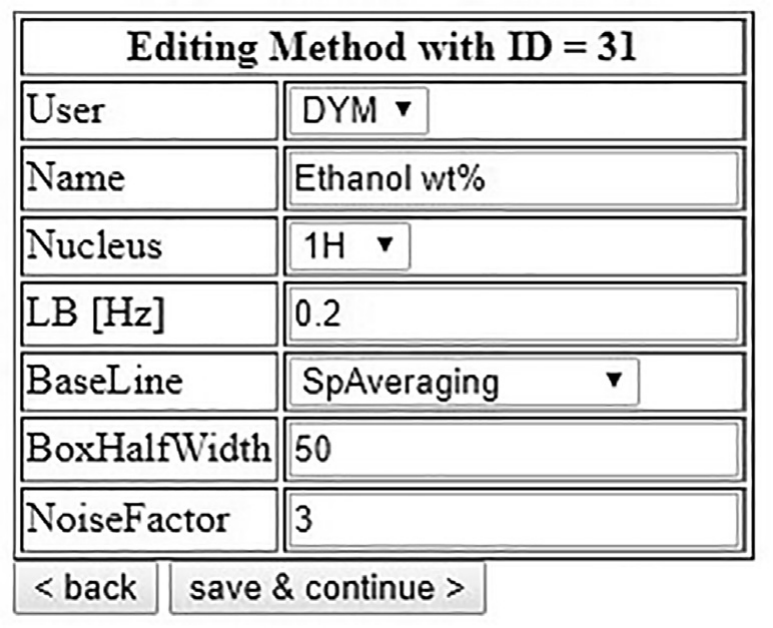
Setup of processing parameters for the determination of the EtOH content of EtOH/H_2_O mixtures.

To set up a processing method, measure a reference spectrum of a sample containing all analytes and internal standard and adjust the parameters in the autosampler software accordingly (Fig. 47). After setting the LB and Baseline parameters in the autosampler’s processing method, click on “Save & continue”.

**Fig. 48:**
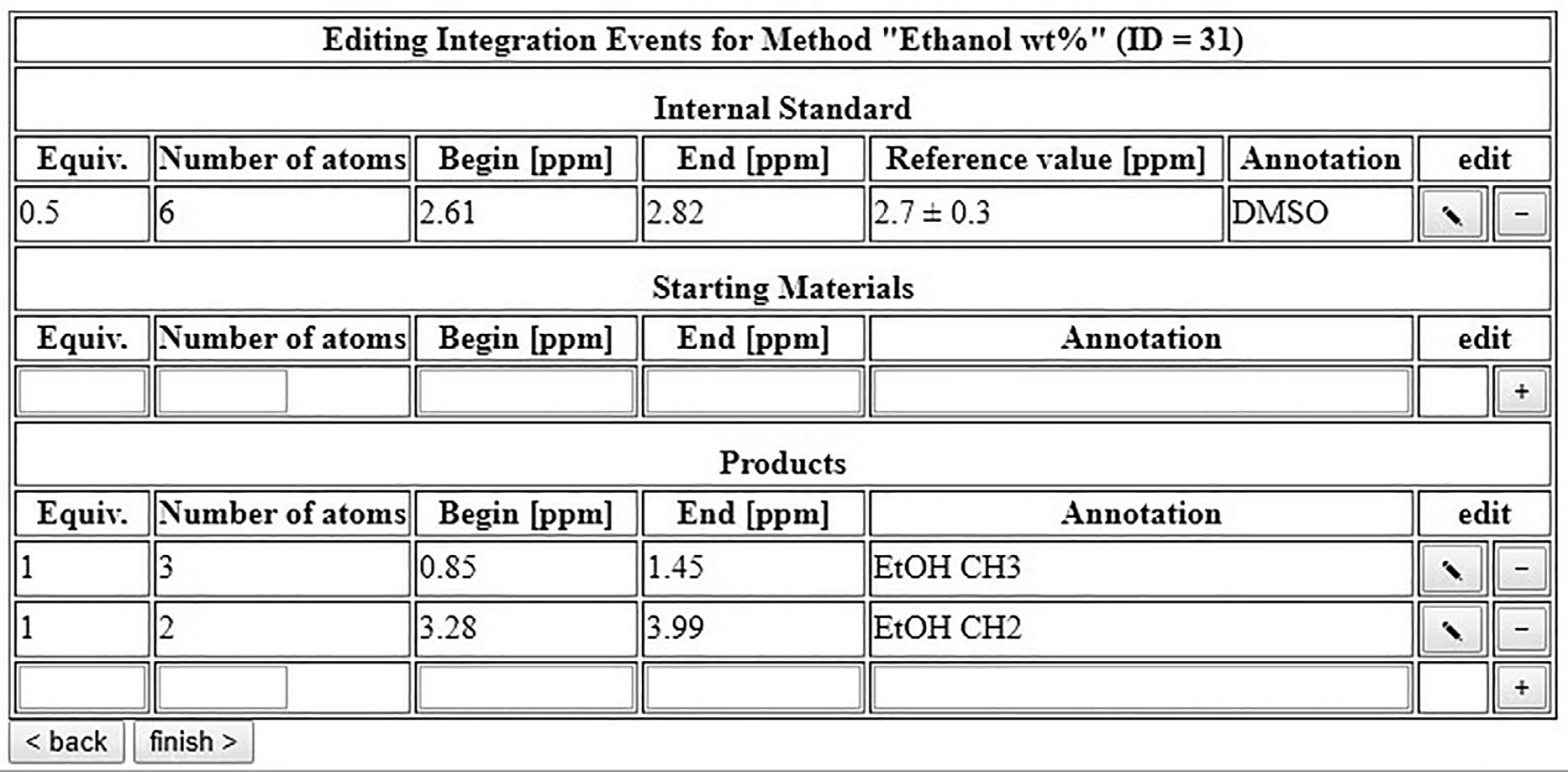
Setup of automatic integration for the determination of ethanol content of an EtOH/H_2_O mixture.

In the next form, automatic integration can be set up (Fig. 48). Use your reference spectrum to find out the chemical shifts of your internal standard and your analytes and enter them into the form. The form requires two additional parameters: “Equiv.” are the equivalents of the given compound relative to the other ones, and “Number of atoms” denotes the number of hydrogen or fluorine atoms per signal. The software will then automatically set the reference value of the spectrum to your internal standard, integrate the areas corresponding to starting materials and products, and calculate conversions and yields. Keep in mind that changing the solvent will result in strong shifts of the signals, so the automatic integration will only give reliable results if the solvent does not change.

A realistic application of a benchtop NMR spectrometer is to continuously monitor the content of an organic material in a medium, for example when checking the amount of an organic contaminant in waste samples, the content of a product synthesized in a student lab, or the ratio of ingredients in a solvent mixture.

**Fig. 49:**
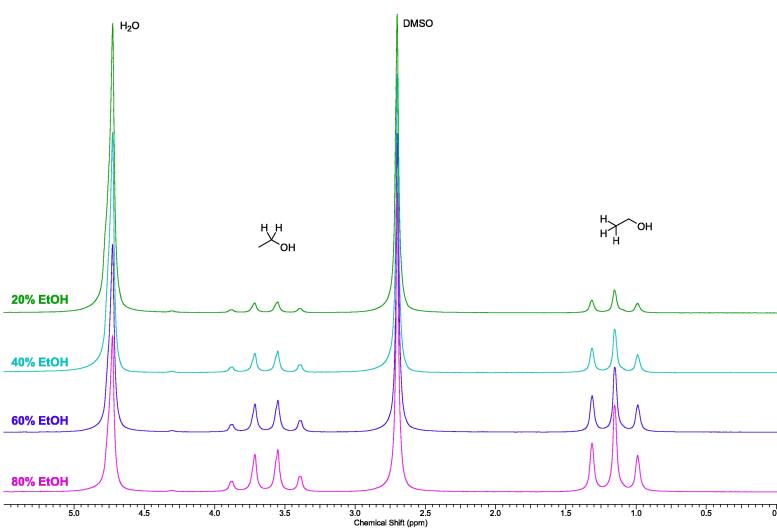
^1^H NMR spectra of the EtOH/H_2_O mixtures.

**Fig. 50:**
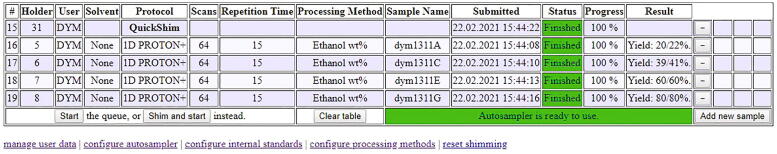
Sample table after the measurement of the EtOH/H_2_O mixtures.

Fig. 49 shows an example series of measurements of mixtures of 20%, 40%, 60% and 80% EtOH in H_2_O. To 1.000 g of the mixtures, 771 µL (10.9 mmol) of dimethylsulfoxide (DMSO) were added as an internal standard. If the sample contained 1.000 g (21.7 mmol) of pure EtOH, the added amount of DMSO would correspond to exactly 0.5 equivalents, which is entered into the processing method accordingly (Fig. 48, fourth row, first column). 10 mg of the resulting mixtures were then diluted with D_2_O to around 600 µL, and the samples were measured using the autosampler. As can be seen from Fig. 50, the automatic integration correctly quantified the amount of EtOH using the CH_3_ and CH_2_ peaks, and directly wrote it into the sample table.

### 6.4 Troubleshooting

#### 6.4.1 Sample stuck inside of the spectrometer

If a sample is stuck in the spectrometer, it can usually be removed as follows: First, the rotor must be dismounted. Then, an empty NMR tube with a cap, on which a piece of double-sided sticky tape is placed, is used to pick out the NMR tube which is stuck. Finally, the rotor is placed back on the autosampler.

Following the removal of the sample, the reason for this issue should be identified. Typically, one of the following problems causes this issue:

The spinner might either not be smooth enough, or the inside walls of the spinner do not align well enough with the outer walls, leading to a skewed position upon inserting the sample. Test if other spinners have the same problem at the same holder position. If they don’t, it is recommended to dispose of the faulty spinner.

The holes of the *Spinsolve* spectrometer, the aluminium baseplate, the main motor holder, the slider, and/or the rotor might not be aligned correctly. Refer to Section 5.1.1, “*Aluminium baseplate with funnel*”, to realign the aluminium baseplate, and Section 5.3.2, “*Fine-tuning the air pressure, slider, and rotor placement*”, to realign the slider and rotor.

#### 6.4.2 Autosampler reports that no sample was detected at the specified position

This error typically occurs if the sample has been placed in the wrong holder, or if the compressed air flow is not sufficient to lift the sample all the way to the sensor in the tower.

First and foremost, check for leaks in the compressed air system. Make sure that only a minimum of air escapes between the carousel and the slider, and that no air escapes between the 3D printed parts of the pneumatic system and the plastic tubes used to guide the air into the spectrometer. If no leaks are found and this issue only occurs only with heavier samples or samples in specific holders, the air flow is not sufficient. The recommended procedure is to increase the air pressure until the stream of air is sufficient to trigger the sensor at the top of the tower even for a rather heavy sample in the least responsive carousel position. Refer to Section 5.3.2, “*Fine-tuning the air pressure, slider, and rotor placement*”, to increase the air flow.

#### 6.4.3 Restarting the autosampler

If the autosampler is not responding, the easiest solution is to restart it. This can be done in the following ways:

On the PC, go to the “Autosampler Satan” software. Under the autosampler control, press the “Disconnect” and then the “Connect” button.

If that did not help, use a non-conductive stick (e.g. wooden cotton swab) to press the “reset” button on the motor shield, which is located right above the hole in the lid of the electronics case.

During the reset, the solenoid valve will open, and the LED lights and LCD display will briefly flash.

#### 6.4.4 Spinner is stuck on the NMR tube

**Fig. 51:**
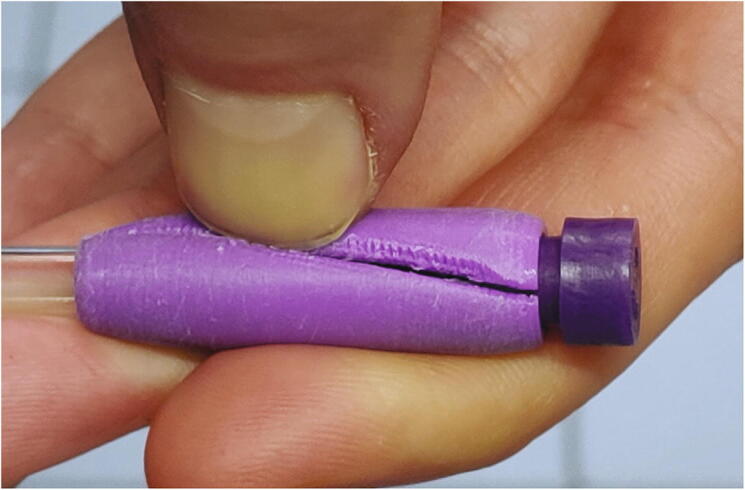
Using a thumbnail to remove a spinner.

If a spinner is fit too tightly around the NMR tube, it might be difficult to remove it. In this case, it is possible to loosen the spinner by pressing a thumbnail into the slit of the spinner (Fig. 51).

## 7 Validation and characterization

The autosampler is able to insert, change, or eject samples in less than 30 s under optimal conditions, making its speed comparable to manual changing of samples (Table 11).

**Table 11:** Times required for adding, changing, or removing samples from the Spinsolve spectrometer.

Operation	Autosampler, best-case^1^	Autosampler, worst-case^2^	Manual
Inserting a sample	23 s	45 s	~20 s
Changing a sample	28 s	51 s	~25 s
Ejecting a sample	14 s	37 s	~10 s

^1^ “Best-case” scenario: Sample in Holder 1 (closest to the zero-point of the rotor), starting position of the rotor so that the rotor does not need to turn during the homing.

^2^ “Worst-case” scenario: Sample in Holder 17 (furthest from the zero-point of the rotor), starting position set so that the rotor must turn for nearly a full revolution during the homing.

However, rather than staying next to the NMR to manually operate it for the entire duration of the measurements, the rotor is charged once with all samples and can then be left unattended until all measurements are finished. The workload for manually inserting and ejecting a sample, entering the measurement parameters in the *Spinsolve* software, and starting a measurement, is around one minute. Measuring 30 samples with 3 min of measurement time plus sample handling would keep the operator busy for around 2 h. During this time, the operator would have to manually process the finished samples. In contrast, using the autosampler, the scientist only spends around 5 s per sample to add a spinner and put the sample into a holder, and around 2 min to add all 30 experiments into the table and start the queue. This adds up to less than 3 min of work without the need to return to the spectrometer until all the measurements are finished. The results of the automated processing, e.g. the content of a selected component in comparison to an internal standard are displayed in the sample table, which can be viewed from any browser.

If several scientists want to use one spectrometer for manual measurements, queues would form with users waiting in line to start their experiments. Using the autosampler, the scientists can add their experiments to the queue, and replace already measured samples with their own sample tubes.

Halbach magnets are known to be sensitive to temperature changes. However, during the entire runtime of the autosampler, the occasional flow of air through the instrument has never changed the temperature to an extent that this would have affected the lock or the shim values.

**Fig. 52:**
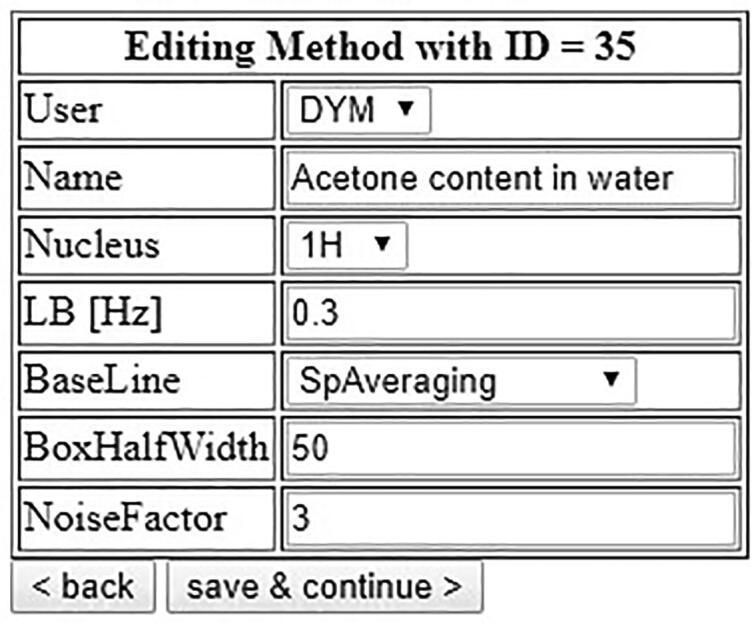
Processing parameters for the determination of acetone content in water.

A realistic application is the use of benchtop-NMR measurements to monitor the content of acetone (or any other organic contaminant) in batches of waste water. This scenario is illustrated in Fig. 54. A defined quantity of 500 mg was taken from 30 batches of material with the help of a pipette. To each sample, exactly 61.2 µL of DMSO were added as an internal standard by an Eppendorf pipette. The experimental details are 8 scans and a repetition time of 15 s. For processing, 0.3 Hz line broadening are applied for the exponential window function, and the baseline correction is applied using ACD’s “spectrum averaging” with the parameters “BoxHalfWidth” set to 50 and “NoiseFactor” set to 3 (Fig. 52).

**Fig. 53:**
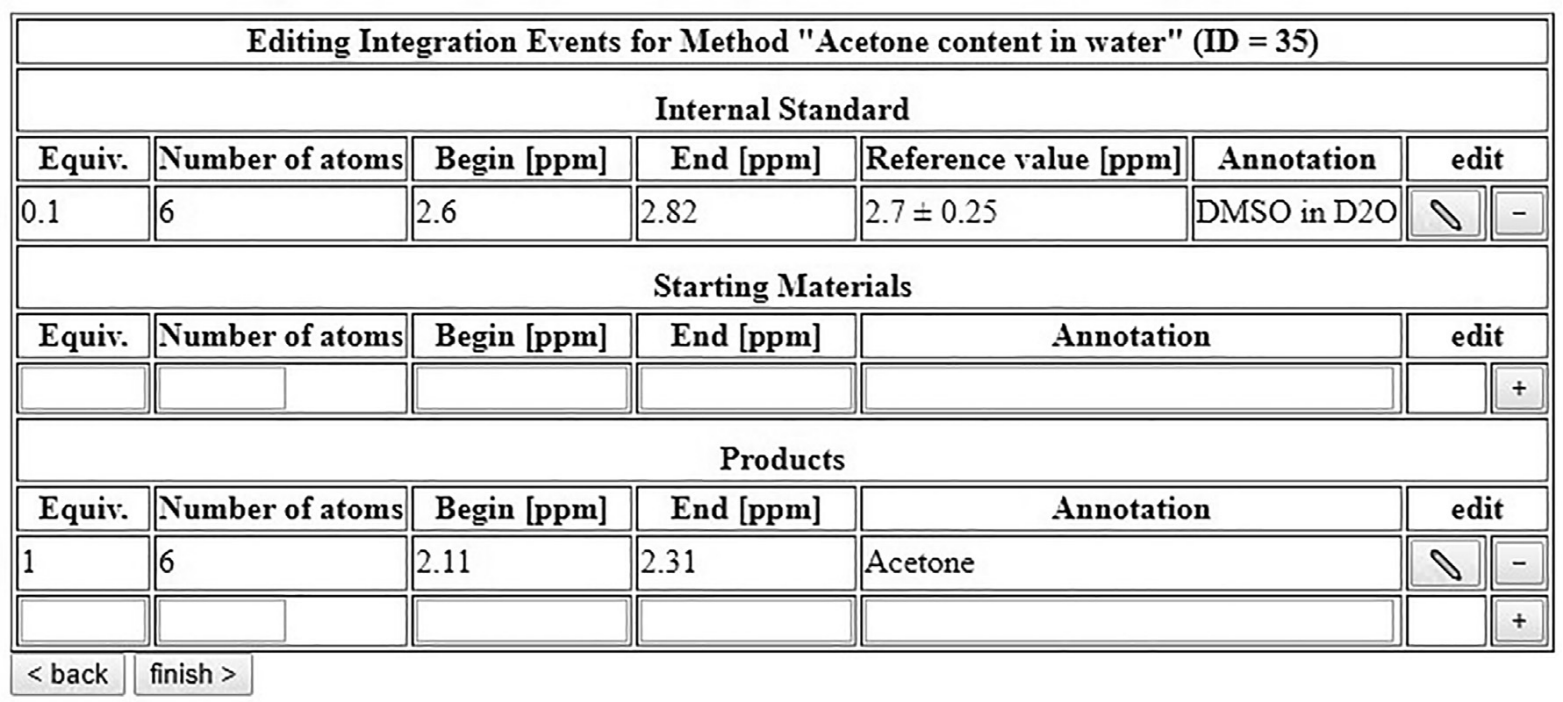
Integration table for the determination of acetone content in water.

Using DMSO (2.70 ppm) as internal standard, the signal of acetone is expected in the region of 2.11–2.31 ppm, while the DMSO is expected at 2.60–2.82 ppm, therefore these regions are pre-set for automatic integration. Both compounds have six protons each, which is entered into the form accordingly. If the acetone content of the sample were 100%, the aliquot of 500 mg would correspond to 8.61 mmol, while the amount of DMSO added to each aliquot is 61.2 µL, which corresponds to 0.861 mmol. Therefore, the equivalents of DMSO are set to 0.1 (See Fig. 53).

**Fig. 54:**
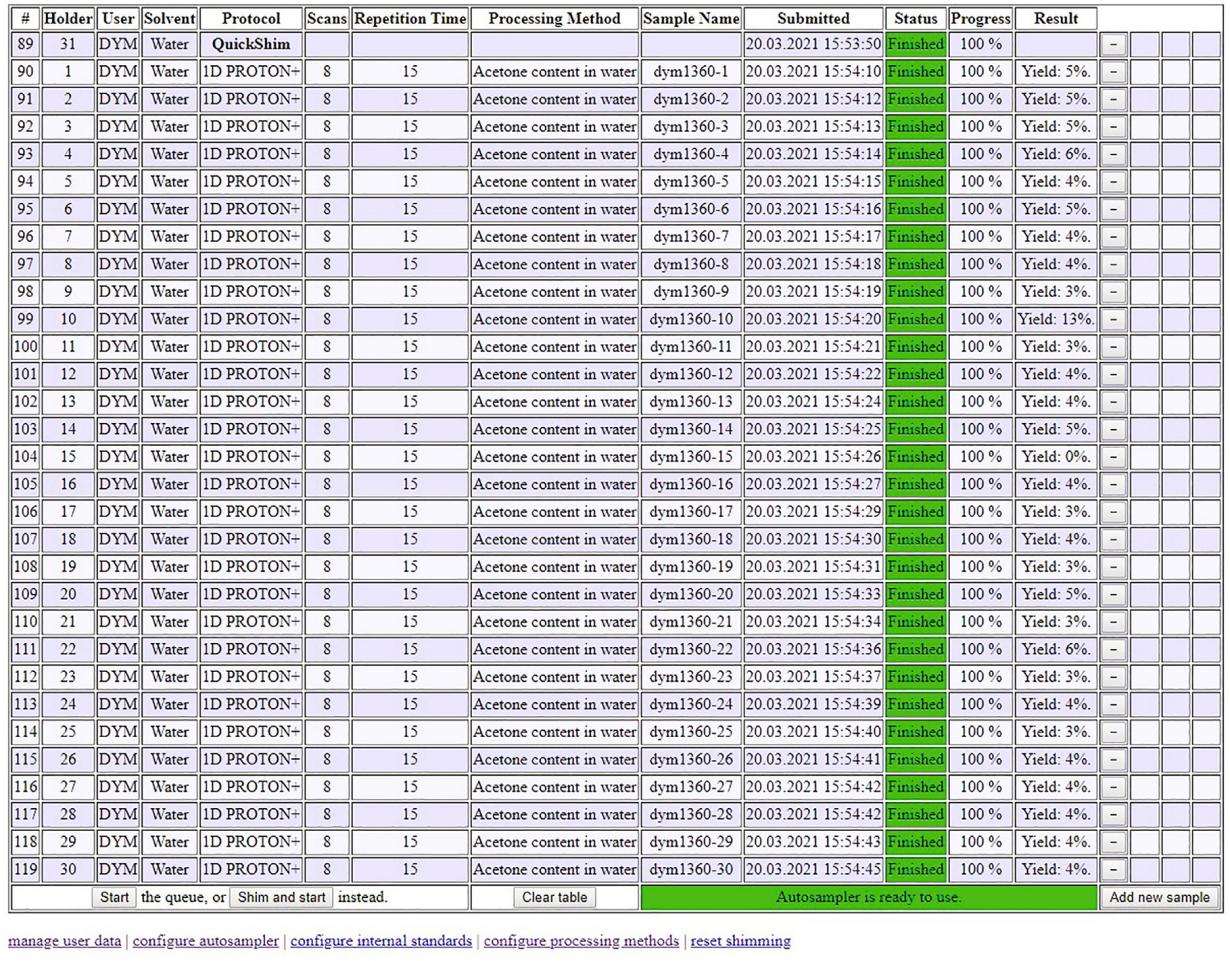
Final sample list of thirty water samples contaminated with acetone.

While the samples are being measured on the NMR equipped with the RotoMate, the operator is alerted in real time that one of the samples, dym1360-10, has significantly more than the 5% acetone content of the others, and requires special attention prior to its disposal (Fig. 54).

Since the sample table is accessible from the browser of all office computers in the local network, it is not even necessary to go to the NMR spectrometer to inspect the results. The operator can use this time to set up a new batch of samples and take care of the waste-water disposal. As soon as the first sample list is finished, the samples in the rotor can be replaced and the next series of samples can be measured.

## CRediT authorship contribution statement

**Marco Dyga:** Software, Conceptualization, Methodology, Validation, Visualization, Writing - original draft, Writing - review & editing. **Christoph Oppel:** Conceptualization, Methodology, Visualization, Writing - review & editing. **Lukas J. Gooßen:** Conceptualization, Methodology, Validation, Writing - original draft, Writing - review & editing, Supervision, Project administration.

## Declaration of Competing Interest

The authors declare that they have no known competing financial interests or personal relationships that could have appeared to influence the work reported in this paper.
