# Supplementary material for: RotoMate: An open-source, 3D printed autosampler for use with benchtop nuclear magnetic resonance spectrometers
Source: HardwareX. 2021 Jun 23;10:e00211. doi: 10.1016/j.ohx.2021.e00211 (PMC9123427; doi:10.1016/j.ohx.2021.e00211)
Supplement: Supplementary data 1 [file mmc1.pdf]

Bill of Materials

All prices were checked from Germany during January 2021. Tools are not included in the calculation of the price.

3D printed parts

All parts were designed using *Autodesk Inventor*. The parts were printed using an *Ultimaker 2+* and the *Cura* slicer. The parts were printed out of polylactide (PLA) from the suppliers *Avistron*, *Verbatim*, or *3DJake*, or from glycol-modified polyethylene terephthalate (PETG) from the supplier *3DJake*. The default print settings are shown in Table 1. For some parts, the settings had to be modified; these changes are shown in Table 2. Parts were printed from PLA unless noted otherwise. Prices are calculated based on the given print settings and a price of 21.99 EUR/kg PLA (*3DJake ecoPLA*, 15.01.2021) and 29.99 EUR/kg PETG (*3DJake PETG*, 28.01.2021).

Table 1: Default print settings.

Description	*Setting*
Nozzle Diameter	0.4 mm
Line Width	0.35 mm
Layer Height	0.25 mm
Wall Line Count	4
Top Layers	5
Bottom Layers	3
Build Plate Adhesion Type	Brim
Brim Width	4 mm
Brim Only on Outside	Yes
Infill Density	25%
Infill Pattern	Cubic Subdivision
Support Placement	Touching Buildplate
Support Overhang Angle	75°

Table 2: List of 3D-printed parts.

Component	*File name (STL)*	*File name (IPT)*	*Qty*	*Print settings*	*Cost per unit – EUR*	*Total cost – EUR*
Tube funnel	Tube_Funnel.stl	Tube Funnel B.ipt	1	No brim, no support, layer height of 0.1 mm	0.20	0.20
Angle	Angle.stl	Stopfen F.ipt	1	Default	0.24	0.24
Plug	Plug.stl	Stopfen F.ipt	1	Default	0.33	0.33
Flow regulator – Inner part	FR_inner.stl	Druckregler A.ipt	1	No brim, no support	0.22	0.22
Flow regulator – Outer part	FR_outer.stl	Druckregler A.ipt	1	Wall Line Count increased to 8	3.52	3.52
Flow regulator – Lid	FR_lid.stl	Druckregler A.ipt	1	No brim, no support	0.09	0.09
Flow regulator – Casing	FR_casing.stl	Druckregler A.ipt	1	Default	1.56	1.56
Flow regulator – Backplate	FR_backplate.stl	Druckregler A.ipt	1	Default	0.29	0.29

Main motor holder	Motorholder.stl	Motorhalterung mit Platte A.ipt	1	Default	1.76	1.76
Guide rail (left)	Guide_Rail_L.stl	Führungsschiene.ipt	1	The side, which will later point towards the slider, must point upwards.	0.07	0.07
Guide rail (right)	Guide_Rail_R.stl	Führungsschiene.ipt	1	The side, which will later point towards the slider, must point upwards.	0.07	0.07
Spring block	Spring_Block.stl	Feder-Klotz.ipt	2	Layer height of 0.125 mm	0.02	0.04
Tower – Shelf	Tower_Shelf.stl	TurmDeckel mit Brett.ipt	1	Default	0.29	0.29
Tower – Lid	Tower_Lid.stl	TurmDeckel mit Brett.ipt	1	Default	0.70	0.70
Tower – Endstop	Tower_Endstop.stl	Turm Wippe.ipt	1	Default, must be printed in **black colour**.	0.07	0.07
Tower – Body	Tower_Body.stl	Turm.ipt	1	No support, brim width of 10 mm	1.74	1.74
Servo holder	Servoholder.stl	PusherServo-Halter.ipt	1	Default	0.20	0.20
Electronics holder – Base plate	EH_Base.stl	Elektronikgehäuse.ipt	1	Default	1.14	1.14
Electronics holder – Clip for shift register	EH_Shiftregister_Clip.stl	Elektronikgehäuse.ipt	2	Default	0.01	0.02
Electronics holder – Keypad holder	EH_Keypad-Holder.stl	Elektronikgehäuse.ipt	1	Default	1.21	1.21
Electronics holder – Display holder	EH_Display-Holder.stl	Elektronikgehäuse.ipt	1	Default	0.46	0.46
Electronics holder – Display holder side panel	EH_Display-Sidepanel.stl	Elektronikgehäuse.ipt	1	Default	0.26	0.26
Electronics holder – Mounting bracket	EH_Mounting-Bracket.stl	Elektronikgehäuse.ipt	1	No brim	0.18	0.18
Electronics Lid	EH_Lid.stl	Elektronikgehäuse.ipt	1	No brim, no support	2.22	2.22
Sliding ring	Sliding_Ring.stl	Motorhalterung mit Platte A.ipt	1	Default	1.45	1.45
Cable duct	Cable_Duct.stl	Motorhalterung mit Platte A.ipt	1	Default	0.18	0.18
Limiter block	Limiter_Block.stl	Stell-Klotz.ipt	1	Layer height of 0.125 mm	0.02	0.02
Slider – Lower body	Slider_Lower.stl	Feder-Schieber B.ipt	1	Layer height of 0.125 mm	0.04	0.04
Slider – Upper body	Slider_Upper.stl	Feder-Schieber B.ipt	1	Layer height of 0.125 mm, **solid infill** (set “Top Layers” to 1000)	0.20	0.20
Slider – Pipe guide holder	Slider_Pipe-Guide-Holder.stl	Feder-Schieber B.ipt	1	Default	0.07	0.07
Slider – Pipe guide	Slider_Pipe-Guide.stl	Feder-Schieber B.ipt	1	Layer height of 0.125 mm, no support	0.02	0.02
Slider – Spacer	Slider_Spacer.stl	Feder-Schieber B.ipt	1	Default	0.01	0.01
Slider – Endstop Flag	Slider_Endstop-Flag.stl	Feder-Schieber B.ipt	1	No brim	0.01	0.01
Pulling	Pulling_Dampener.stl	Lenkungsdaempfer	1	Support Placement: Everywhere	0.04	0.04

dampener		C.ipt				
Drive shaft	Drive_Shaft.stl	Schubstange.ipt	1	Default	0.04	0.04
Rotor – Lower Ring	Rotor_Lower.stl	Rotor 3dDrucker mit Röhren A.ipt	1	Default. Use a different colour than that of the slider.	1.50	1.50
Rotor – Middle Ring	Rotor_Middle.stl	Rotor 3dDrucker mit Röhren A.ipt	1	Default. Use a different colour than that of the slider.	1.58	1.58
Rotor – Upper Ring	Rotor_Upper.stl	Rotor 3dDrucker mit Röhren A.ipt	1	Default. Use a different colour than that of the slider.	0.77	0.77
Flag holder	Flag_Holder.stl	Fähnchenhalter.ipt	1	No brim, Support Placement: Everywhere. Must be printed in **black colour**.	0.07	0.07
Motor flange	Motor_Flange.stl	Motorflansch.ipt	1	Default, **print from PETG**.	0.87	0.87
Clutch ring	Clutch.stl	Exzenter A.ipt	1	Default	0.40	0.40
Spinner	Spinner.stl	Spinner E.ipt	32	Layer Height of 0.125 mm, no support, brim width of 8 mm	0.04	1.41
**Total (1.15 kg PLA):**						**€ 25.56**

Off-the-shelf parts (excluding screws):

Table 3: List of off-the-shelf parts.

Component	*Amount*	*Cost per unit – EUR*	*Total cost – EUR*	*Source of materials*
Aluminium plate (40 cm × 30 cm × 3 mm)	1	13.97	13.97	https://www.alufritze.de/alublech-3-0-mm-aus-almg3.html
PMMA or Polycarbonate pipe, OD=15 mm, ID=11 mm	7 m	36.06	36.06	https://www.plattenshop24.com/rohre-stabe/213-997-rohre.html#/44-farbe-transparent/148-wandstarke-2_mm/152-durchmesser_aussen-15_mm
Mousepad	1	2.00	2.00	
Filter fleece	-	-	9.99	Typically sold as replacement parts for kitchen hoods, vacuum cleaners, etc. https://www.amazon.de/Mikrofilter-Motorfilter-Abluftfilter-Microfilter-zuschneidbar/dp/B086PFYJZ7/
Pressure regulator with oil trap	1	28.95	28.95	https://www.amazon.de/%C3%96labscheider-Wasserabscheider-Wartungseinheit-Teilig-Manometer/dp/B019IZXWIE
Connectors	2	-	9.99	Example, pack of 5: https://www.amazon.de/St%C3%BCck-Steckverschraubung-Schnellsteckverbinder-Push-6mm/dp/B0108HHH3C?th=1 The connector type you need depends on the connectors present on your pressure regulator.
Activated charcoal filter	1	7.99	7.99	https://www.ebay.de/itm/PCF-2-Inline-Filterkartusche-CL10T33-Aktivk-T-G-W-1-4-IG-fuer-WP-2-Ultrafilter-/371711530335?_trksid=p2385738.m4383.l4275.c10
Tubing for pressurized air (6 mm or 1/4")	2 m	2.88 / m	5.76	Adjust length according to your needs. Total price calculated for a length of 2 meters. https://webshop.schachermayer.com/cat/de-AT/product/festo-pun-druckluftschlauch-dm-6-mm-schwarz/108633006
O-Ring, 30×3.5 mm	1	0.98	5.88	Pack of 6: https://www.hug-technik.com/shop/product_info.php?info=p2740
O-Ring, 30×2.0 mm	1	0.70	5.60	Pack of 8: https://www.hug-technik.com/shop/product_info.php?info=p2736
O-Ring, 13×2.5 mm	1	0.82	5.74	Pack of 7: https://www.hug-technik.com/shop/product_info.php?info=p23995
O-Rings, 10×2.5 mm	3	0.39	5.07	Pack of 13: https://www.hug-technik.com/shop/product_info.php?info=p2437
Adhesive PTFE foil	1	0.88	0.88	It might be difficult to find a supplier who is willing to sell this

(without glass fibre), ca 5×10 cm				material in low quantities. https://www.apsoparts.com/ishop/de-CH/Kunststofftechnik/Fertigteile,%20Schutzstopfen,%20Schutznetze/Selbstklebeb%C3%A4nder/PTFE%20Folie/PTFE%20Folie%20selbstklebend,%20Typ%20SPSA%20f%C3%BCr%20SKAP/product/node/14233/product/01.1030.31/article/01.1030.5050.xhtml
Silencer (1/4” thread)	1	6.99	6.99	https://www.conrad.de/de/p/norgren-druckluft-schalldaempfer-m-s2-aussengewinde-1-4-10-bar-kunststoff-1-st-582055.html
Steel pins 30×3 mm	2	7.89	7.89	Pack of 25: https://www.amazon.de/sourcing-map-D%C3%BCbelstift-Zylindrischer-Regalbodentr%C3%A4gerstift/dp/B07YL2XP7D/?th=1
Ball bearings, 3×12×4 mm with V-groove	3	6.39	6.39	Pack of 20: https://www.amazon.de/stücke-V623ZZ-Kugellager-Riemenscheibe-3x12x4mm/dp/B07MDG835Q/
Schneider Slider Memo pen	1	2.35	2.35	https://schneiderpen.com/de_de/kugelschreiber/slider-memo-schwarz-xb-4004675064202/
Servos Carson CS-13	2	30.59	61.18	https://www.haertle.de/index.php?cl=details&cnid=77648da7b1d56ddb7.70369005&anid=82b0e629c5d4d7822a75c50eeebc81d6&listtype=list&
Compression springs, VD-052F	10	1.73	17.30	Pack of 10: https://www.federnshop.com/de/produkte/druckfedern/vd-052f.html
Tension spring, RZ-045E-02I	1	2.83	2.83	https://www.federnshop.com/de/produkte/zugfedern/rz-045e-02i.html
Arduino Uno R3	1	20.00	20.00	https://store.arduino.cc/arduino-uno-rev3
Motor shield, DFRobot DRV8825	1	20.21	20.21	https://botland.store/arduino-shield-motor-controllers/2690-dfrobot-drv8825-2-channel-stepper-motor-driver-45v-25-a-shield-for-arduino.html For the manufacturer’s documentation, see: https://wiki.dfrobot.com/Stepper_Motor_Shield_For_Arduino_DRV8825__SKU_DRI0023
Solenoid valve, MARCO EV130 (12V version)	1	19.99	19.99	https://www.truckstyler-shop.de/Magnetventil-fuer-Drucklufthoerner-12-V For the manufacturer’s documentation, see: https://items.marco.it/en/products/ev130-electric-valve-blister.html
Stepper motor NEMA 23 × 56 mm, 4-wire	1	22.99	22.99	The motors we have used are no longer available. Below, a similar model is linked. https://www.amazon.de/-/en/Quilted-23-Stepper-1-26NM-Conductor-Printer/dp/B06XVC23Z7/ In principle, any 4-wire NEMA 23 stepper motor with a height of 56 mm can be used. However, some low-quality motors do not operate smoothly at low speeds and should be avoided. It is impossible to judge the motor’s quality from the online offer, some trial and error might be necessary.
Optical endstop switch modules, TCST2103	3	1.00	3.00	https://www.ebay.de/itm/Lichtschranke-Optical-Endstop-Switch-TCST2103-fur-Arduino-Raspberry-Pi/272573201403?hash=item3f76a0fbfb:g:ER4AAOSwOgdYtmqT
Shift register module, PCF8574T	1	2.21	2.21	https://www.ebay.de/itm/Arduino-PCF8574-PCF8574T-I2C-8-Bit-IO-GPIO-Expander-Modul-HimbeRSPF-/264817936748
Active buzzer module	1	1.88	1.88	https://www.ebay.de/i/283974309432
LED traffic light module	1	3.99	3.99	https://www.amazon.de/dp/B086TRY65Y?th=1
LCD Display, LCM1602 with I2C bus	1	5.90	5.90	https://www.funduinoshop.com/epages/78096195.sf/de_DE/?ObjectPath=/Shops/78096195/Products/A-5-5
Matrix keypad (4x4)	1	1.00	1.00	https://www.ebay.de/itm/4x4-Matrix-Array-16-Key-Membrane-Keypad-Keyboard-AVR-12V-Arduino-Module/233856411276
Diode (12 V)	1	0.19	0.19	https://www.conrad.de/de/p/diotec-si-gleichrichterdiode-1n4001-do-204al-50-v-1-a-162213.html
Lustre terminals	2	0.05	5.95	Pack of 120: https://wittko.eu/luesterklemmen-25-qmm-12-teilig-10-stangen

DC/DC Converter (12V to 5V), 15W	1	4.85	4.85	Typically sold under automotive parts, since they are used to provide USB power from car batteries. https://www.amazon.de/Ecloud-Shop-Wechselrichter-Spannungswandler-Konverter/dp/B00D1GQA5O
DC plug 2.5×5.5 mm	1	4.62	4.62	https://www.conrad.de/de/p/tru-components-niedervolt-steckverbinder-buchse-einbau-vertikal-5-5-mm-2-5-mm-1-st-1564887.html
Power supply, 12V, 48W, 2.5×5.5 mm jack	1	14.99	14.99	https://www.amazon.de/dp/B001W3W12E/
Heat-shrink tube set	1	4.98	4.98	https://www.amazon.de/ChiliTec-12000058-Chilitec-Schrumpfschlauch-Sortiment-100-teilig/dp/B003H9CJ1Y
Blade receptacle, 6.3 mm	2	0.45	0.90	https://www.conrad.de/de/p/vogt-verbindungstechnik-3903-flachsteckhuelse-steckbreite-6-3-mm-steckdicke-0-8-mm-180-teilisoliert-rot-1-st-736945.html
Electric wire ferrule set	1	15.99	15.99	https://toom.de/p/aderendhuelsen-set-isoliert-05-25-mm/9062691
Set of wires	1	6.99	6.99	https://www.conrad.de/de/p/joy-it-rb-cb2-30-jumper-kabel-raspberry-pi-40x-drahtbruecken-buchse-40x-drahtbruecken-buchse-30-00-cm-bunt-inkl-pins-1182192.html
Set of Dupont Jumper Connectors (optional)	1	9.99	9.99	https://www.amazon.de/haljia-Dupont-Jumper-Header-Stecker-310PCS/dp/B06WWB66WL
**Total**			**€ 413.43**	

Screws, bolts, nuts

Table 4: List of screws, bolts, nuts, and related items.

Qty	*Size*	*Type*	*Standard*	*Cost per unit – EUR*	*Total cost – EUR*	*Source of materials*
13	3×10 mm	Flat-head wood screws	DIN 7505-B	0.08	4.09	Pack of 50: https://www.amazon.de/St%C3%BCck-Spanplattenschrauben-3x10-Halbrundkopf-Holzschrauben/dp/B084GRH5PS May be replaced by any other type of 3×10 mm flat-headed wood screws.
2	M2×6 mm	Cylinder-head bolts	DIN 84	0.10	2.55	Pack of 25: https://www.schraubenshop24.com/Zylinderschrauben-mit-Schlitz-DIN-84-Edelstahl-A2-M-2-6-mm-25-Stk
4	M2×10 mm	Cylinder-head bolts	DIN 84	0.10	2.55	Pack of 25: https://www.schraubenshop24.com/Zylinderschrauben-mit-Schlitz-DIN-84-Edelstahl-A2-M-2-10-mm-25-Stk
4	M2	Nuts	DIN 934	0.03	0.30	Pack of 10: https://rc-schrauben.de/10x-Stahl-Mutter-M2
2	M2	Washers, d=7 mm	DIN 9021	0.08	1.99	Pack of 25: https://www.amazon.de/Unterlegscheiben-DIN-9021-Karosseriescheibe-Edelstahl/dp/B07B4YQY6W
34	M3×10 mm	Countersunk bolts	DIN 965	0.026	2.55	Pack of 100: https://www.schraubenshop24.com/Senkschrauben-DIN-965-48-mit-Kreuzschlitz-H-galv-verzinkt-3-mm-10-mm-100-Stk
4	M3×16 mm	Countersunk bolts	DIN 965	0.026	2.55	Pack of 100: https://www.schraubenshop24.com/Senkschrauben-DIN-965-48-mit-Kreuzschlitz-H-galv-verzinkt-3-mm-16-mm-100-Stk
18	M3×6 mm	Cylinder-head bolts	DIN 84	0.10	2.55	Pack of 25: https://www.schraubenshop24.com/Zylinde

						rschrauben-mit-Schlitz-DIN-84-Edelstahl-A2-M-3-6-mm-25-Stk
15	M3×10 mm	Cylinder-head bolts	DIN 84	0.10	2.55	Pack of 25: https://www.schraubenshop24.com/Zylinderschrauben-mit-Schlitz-DIN-84-Edelstahl-A2-M-3-10-mm-25-Stk
4	M3×10 mm	Cylinder-head bolts	DIN 912	0.018	0.92	Pack of 50: http://schrauben-niro.de/product/de/Schrauben/Innensechskant-Zylinderschrauben-DIN-912-Edelstahl/V2A-A2-Edelstahl/30-mm/Gewindeschrauben-DIN912-M3x10-rd-mm-A2-50-Zylinder-Innensechskant-25-mm.html
4	M3×30 mm	Cylinder-head bolts	DIN 912	0.10	2.55	Pack of 25: https://www.schraubenshop24.com/Zylinderschrauben-mit-Innensechskant-DIN-912-Edelstahl-A2-M-3-30-mm-25-Stk
1	M3	Threaded rod	-	1.50	1.50	https://www.hornbach.de/shop/Gewindestange-1-m-DIN-976-M3-Edelstahl-A2/3831293/artikel.html
7	M3	Nuts	DIN 934	0.03	0.30	Pack of 10: https://rc-schrauben.de/10x-Stahl-Mutter-M3
14	M3	Washers, d=7 mm	DIN 125	0.02	0.28	https://www.schrauben-schneider.eu/epages/SSD.sf/sec1d457bad56/?ObjectPath=/Shops/SSD/Products/200103032
3	M5×16 mm	Cylinder-head bolts	DIN 912	0.044	1.10	Pack of 25: http://schrauben-niro.de/product/de/Schrauben/Innensechskant-Zylinderschrauben-DIN-912-Edelstahl/V2A-A2-Edelstahl/50-mm/Gewindeschrauben-DIN912-M5x16-rd-mm-A2-70-Zylinder-Innensechskant-4-mm.html
1	M5×20 mm	Cylinder-head bolts	DIN 912	0.05	1.25	Pack of 25: http://schrauben-niro.de/product/de/Schrauben/Innensechskant-Zylinderschrauben-DIN-912-Edelstahl/V2A-A2-Edelstahl/50-mm/Gewindeschrauben-DIN912-M5x20-rd-mm-A2-70-Zylinder-Innensechskant-4-mm.html
4	M5×70 mm	Countersunk bolts	DIN 965	0.10	4.87	Pack of 50: https://www.schraubenshop24.com/Senkschrauben-DIN-965-48-mit-Kreuzschlitz-H-galv-verzinkt-5-mm-70-mm-50-Stk
10	M5×100 mm	Countersunk bolts	DIN 965	0.78	7.84	Pack of 10: https://www.amazon.de/-/en/PROTECH-Phillips-Countersunk-Screws-Stainless/dp/B07VG7GKZG/
10	M5	Nuts	DIN 934	0.03	0.30	Pack of 10: https://rc-schrauben.de/10x-Stahl-Mutter-M5
12	M5	Washers, d=15 mm	DIN 9021	0.07	0.84	https://www.schrauben-schneider.eu/epages/SSD.sf/de_DE/?ObjectPath=/Shops/SSD/Products/200203053
18	M3	Threaded heat inserts	-	27.99	27.99	Pack of 270 pieces “Ruthex Threaded Inserts”, M2/M3/M4/M5: https://www.amazon.de/gp/product/B08K1BVGN9/
1	M5	Threaded heat insert	-			

Total:	€ 71.42

Grand total

Subsection	*Cost – EUR*
3D printed parts	25.56
Off-the-shelf parts	413.43
Screws, bolts, nuts	71.42
**Grand total:**	**€ 510.41**

Tools

Tool	*Tool*	*Tool*
Drill	Set of metric allen keys	Soldering iron
2 mm drill bit	Flathead screwdriver	Cutter knife
2.5 mm drill bit	Philips screwdrivers (PH0 and PH2)	Saw
4.2 mm drill bit	M3 and M5 taps	Hot glue gun
5 mm drill bit	Scissors	Side cutter
12 mm drill bit	Heat gun	Crimping tool
Rubber mallet	Set of metric wrenches	Cable stripper (optional)
Adjustable hand reamer	Pipe wrench	
