# Supplementary figures and images for: RotoMate: An open-source, 3D printed autosampler for use with benchtop nuclear magnetic resonance spectrometers
Source: HardwareX. 2021 Jun 23;10:e00211. doi: 10.1016/j.ohx.2021.e00211 (PMC9123427; doi:10.1016/j.ohx.2021.e00211)

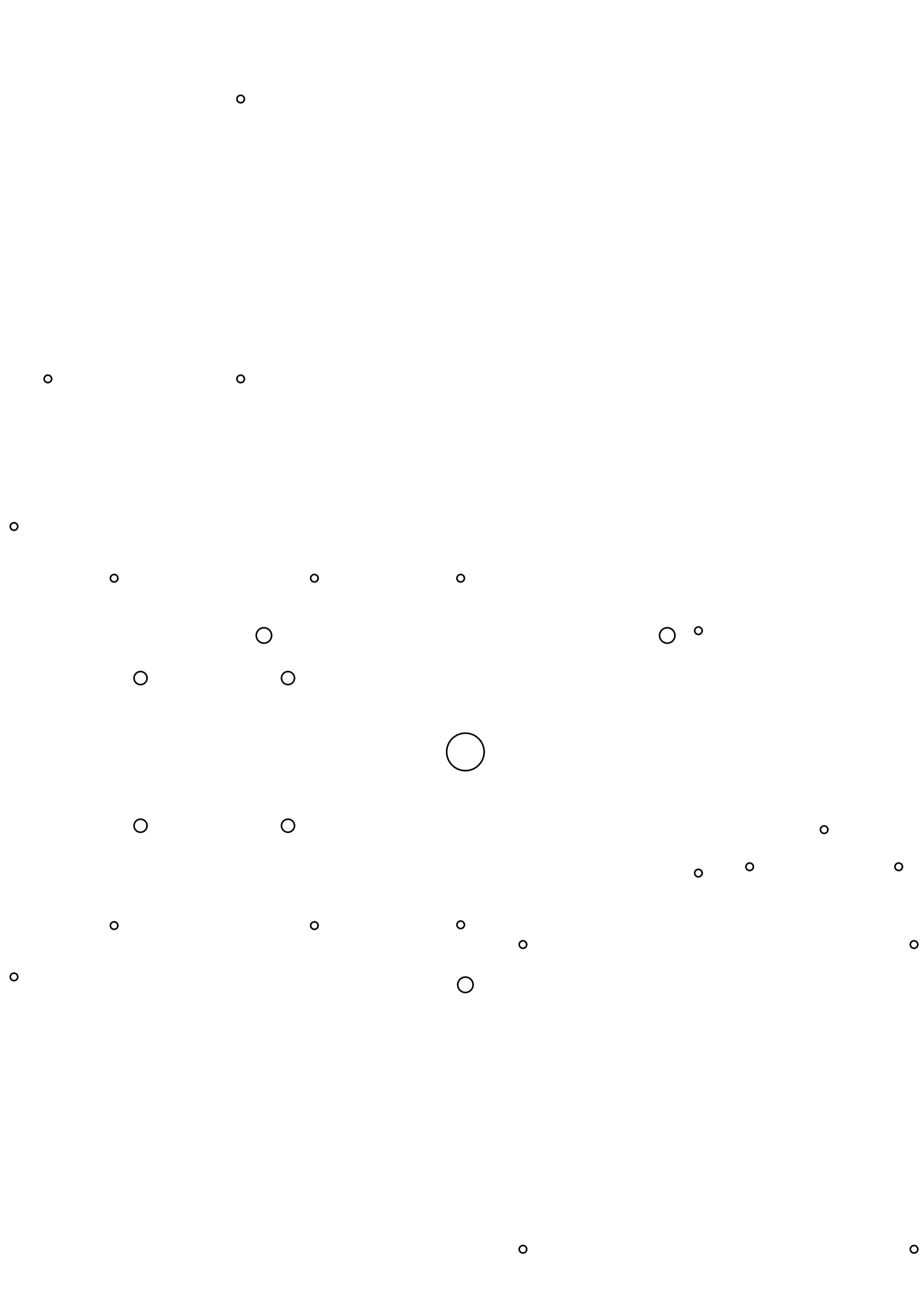

Supplement: Supplementary data 2 [file mmc2.pdf]

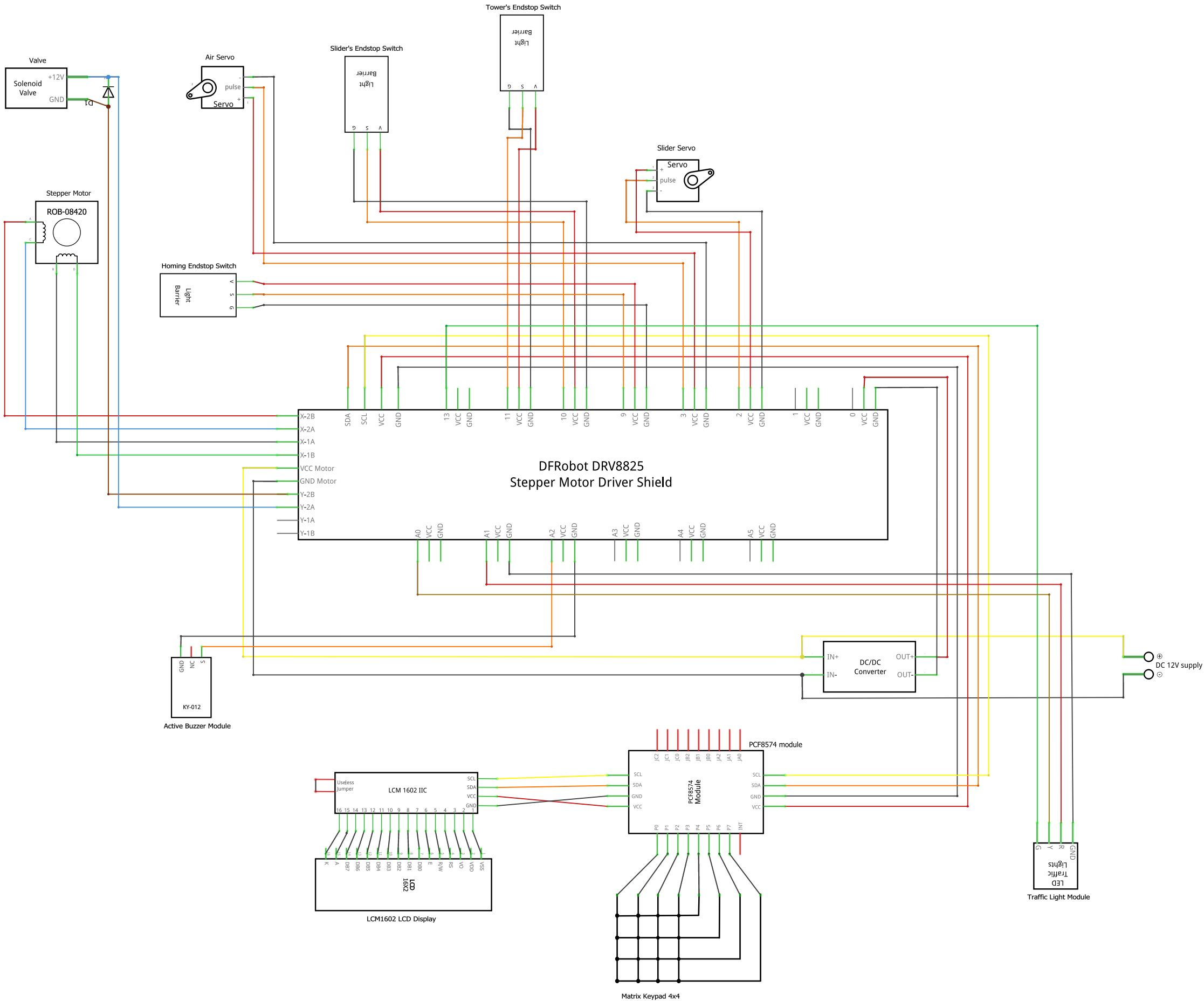

Supplement: Supplementary data 4 [file mmc4.pdf]
