# Supplementary material for: RotoMate: An open-source, 3D printed autosampler for use with benchtop nuclear magnetic resonance spectrometers
Source: HardwareX. 2021 Jun 23;10:e00211. doi: 10.1016/j.ohx.2021.e00211 (PMC9123427; doi:10.1016/j.ohx.2021.e00211)
Supplement: Supplementary data 3 [file mmc3.pdf]

Manual Control
Calibration

Toggle Slider

Toggle air flow

Dismiss Error
Exit
